# Supplementary figures and images for: Rspo3-mediated metabolic liver zonation regulates systemic glucose metabolism and body mass in mice
Source: PLoS Biol. 2025 Jan 24;23(1):e3002955. doi: 10.1371/journal.pbio.3002955 (PMC11759367; doi:10.1371/journal.pbio.3002955)

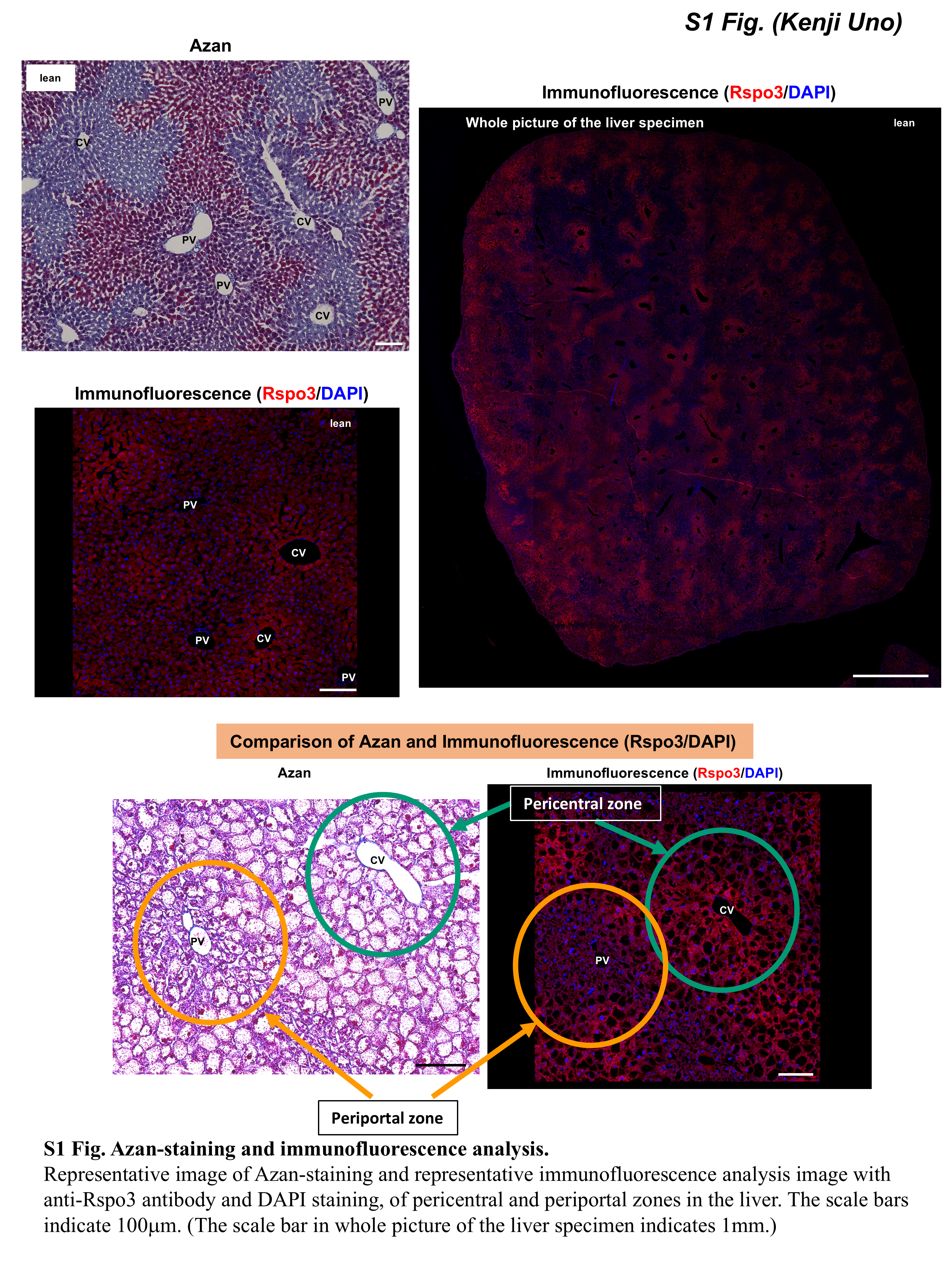

Supplement: S1 Fig — Representative image of Azan-staining and representative immunofluorescence analysis image with anti-Rspo3 antibody and DAPI staining, of pericentral and periportal zones in the liver. The scale bars indicate 100 μm. (The scale bar in whole picture of the liver specimen indicates 1 mm.) (TIF) [file pbio.3002955.s001.tif]

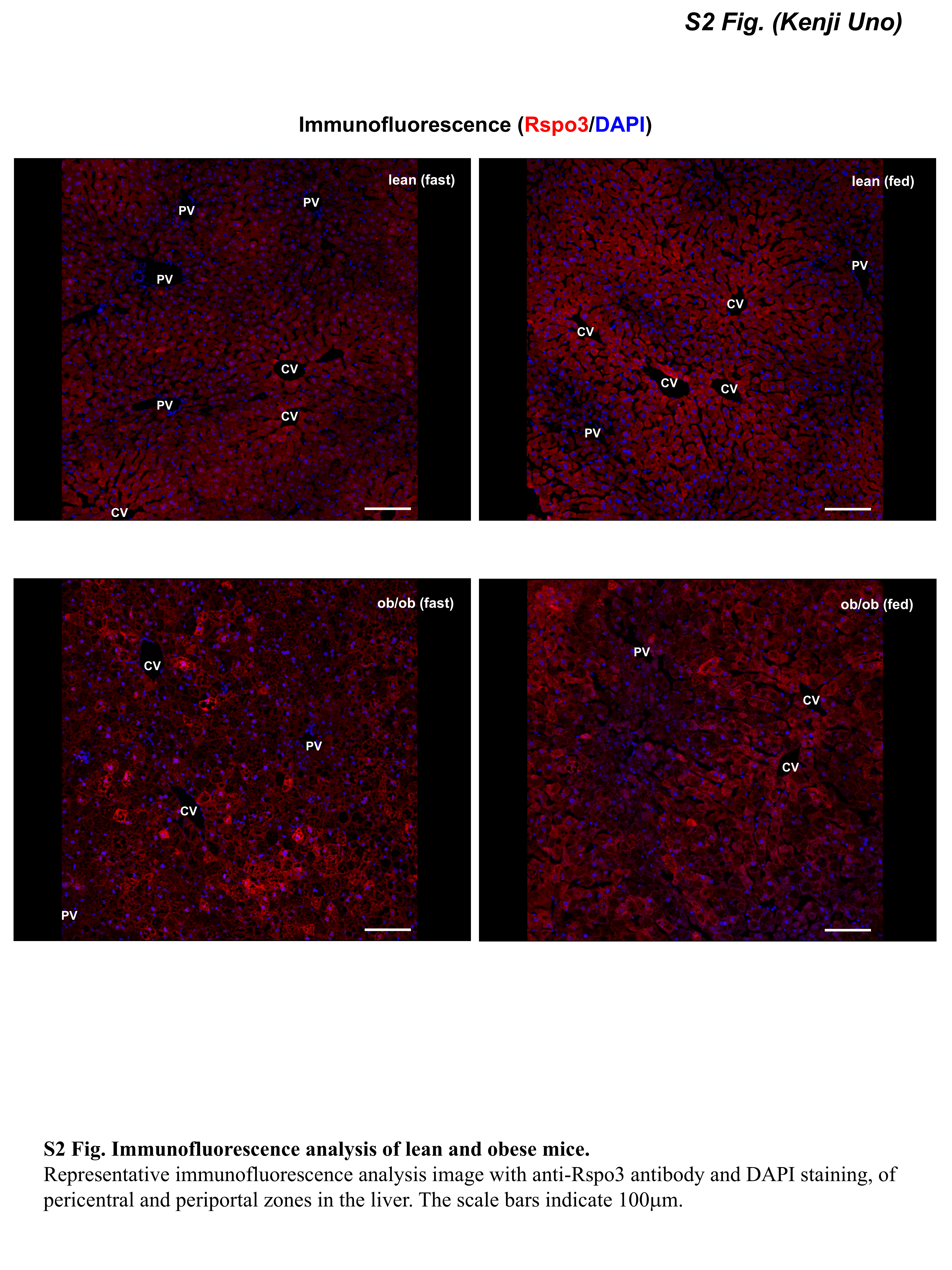

Supplement: S2 Fig — Representative immunofluorescence analysis image with anti-Rspo3 antibody and DAPI staining of pericentral and periportal zones in the liver. The scale bars indicate 100 μm. (TIF) [file pbio.3002955.s002.tif]

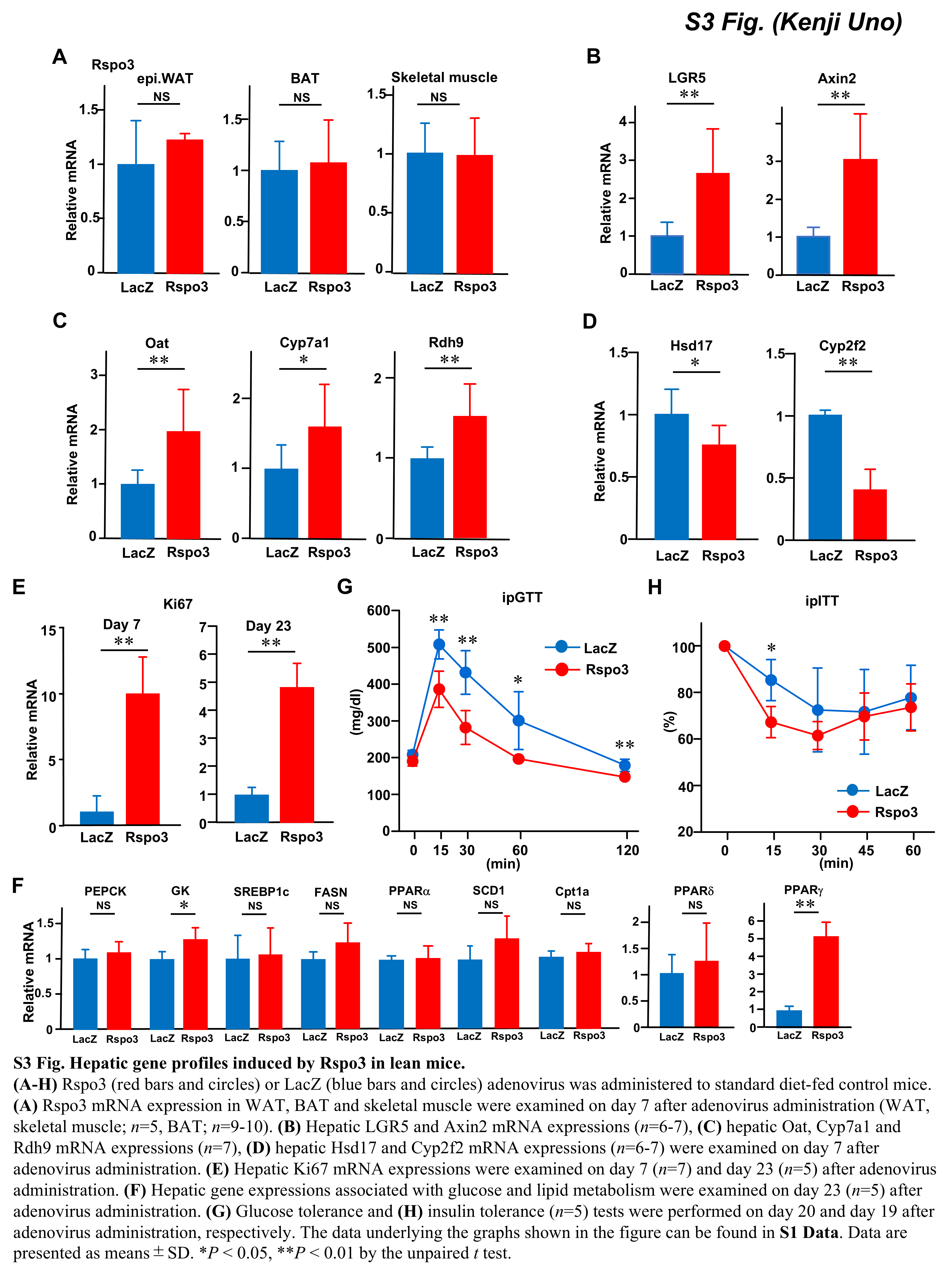

Supplement: S3 Fig — (A–H) Rspo3 (red bars and circles) or LacZ (blue bars and circles) adenovirus was administered to standard diet-fed control mice. (A) Rspo3 mRNA expression in WAT, BAT, and skeletal muscle were examined on day 7 after adenovirus administration (WAT, skeletal muscle; n = 5, BAT; n = 9–10). (B) Hepatic LGR5 and Axin2 mRNA expressions (n = 6–7), (C) hepatic Oat, Cyp7a1, and Rdh9 mRNA expressions (n = 7), (D) hepatic Hsd17 and Cyp2f2 mRNA expressions (n = 6–7) were examined on day 7 after adenovirus administration. (E) Hepatic Ki67 mRNA expressions were examined on day 7 (n = 7) and day 23 (n = 5) after adenovirus administration. (F) Hepatic gene expressions associated with glucose and lipid metabolism were examined on day 23 (n = 5) after adenovirus administration. (G) Glucose tolerance and (H) insulin tolerance (n = 5) tests were performed on day 20 and day 19 after adenovirus administration, respectively. The data underlying the graphs shown in the figure can be found in S1 Data. Data are presented as means ± SD. *P < 0.05, **P < 0.01 by the unpaired t test. (TIF) [file pbio.3002955.s003.tif]

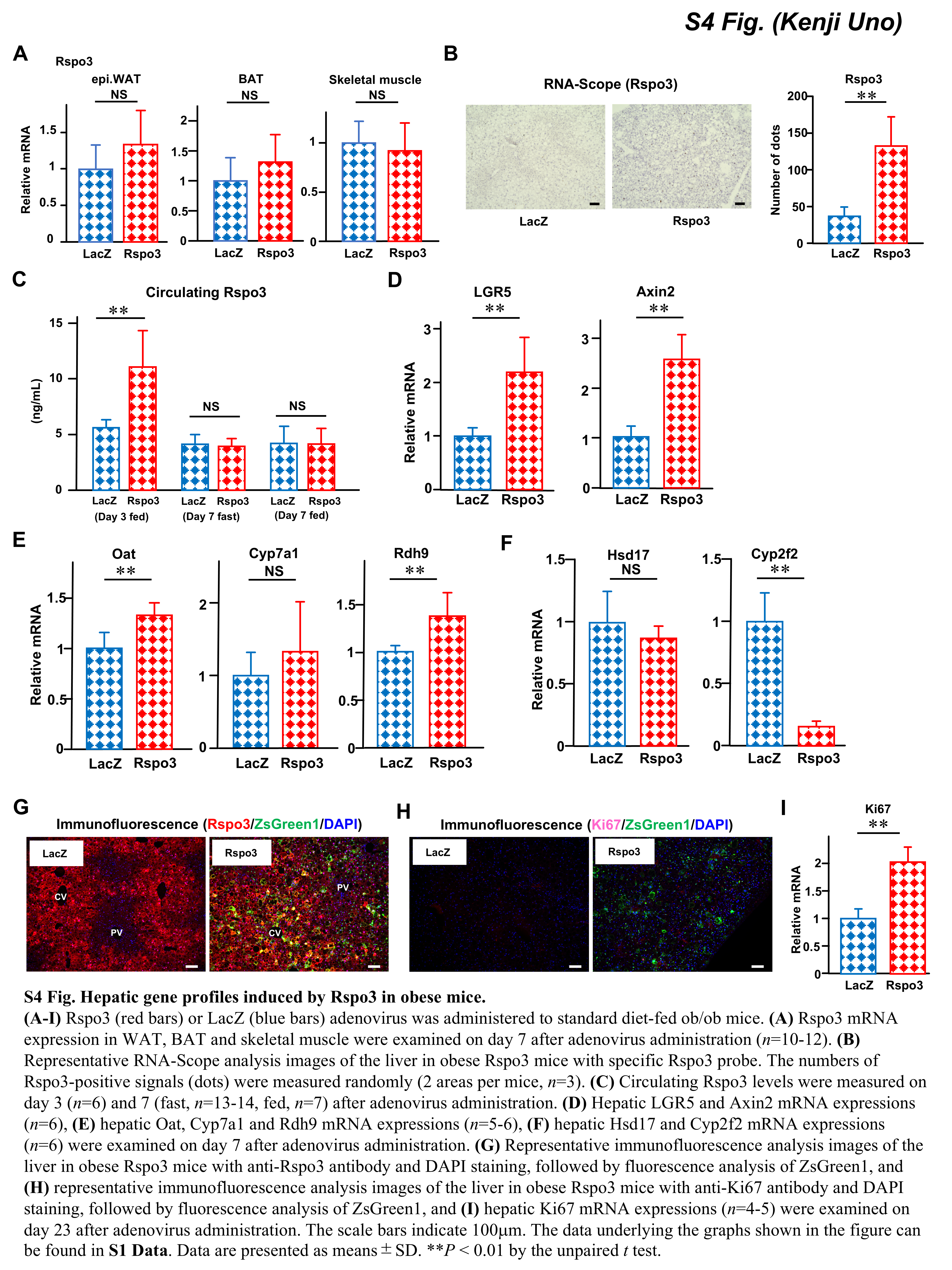

Supplement: S4 Fig — (A–I) Rspo3 (red bars) or LacZ (blue bars) adenovirus was administered to standard diet-fed ob/ob mice. (A) Rspo3 mRNA expression in WAT, BAT, and skeletal muscle were examined on day 7 after adenovirus administration (n = 10–12). (B) Representative RNA-Scope analysis images of the liver in obese Rspo3 mice with specific Rspo3 probe. The numbers of Rspo3-positive signals (dots) were measured randomly (2 areas per mice, n = 3). (C) Circulating Rspo3 levels were measured on day 3 (n = 6) and 7 (fast, n = 13–14, fed, n = 7) after adenovirus administration. (D) Hepatic LGR5 and Axin2 mRNA expressions (n = 6); (E) hepatic Oat, Cyp7a1, and Rdh9 mRNA expressions (n = 5–6); (F) hepatic Hsd17 and Cyp2f2 mRNA expressions (n = 6) were examined on day 7 after adenovirus administration. (G) Representative immunofluorescence analysis images of the liver in obese Rspo3 mice with anti-Rspo3 antibody and DAPI staining, followed by fluorescence analysis of ZsGreen1, and (H) representative immunofluorescence analysis images of the liver in obese Rspo3 mice with anti-Ki67 antibody and DAPI staining, followed by fluorescence analysis of ZsGreen1, and (I) hepatic Ki67 mRNA expressions (n = 4–5) were examined on day 23 after adenovirus administration. The scale bars indicate 100 μm. The data underlying the graphs shown in the figure can be found in S1 Data. Data are presented as means ± SD. **P < 0.01 by the unpaired t test. (TIF) [file pbio.3002955.s004.tif]

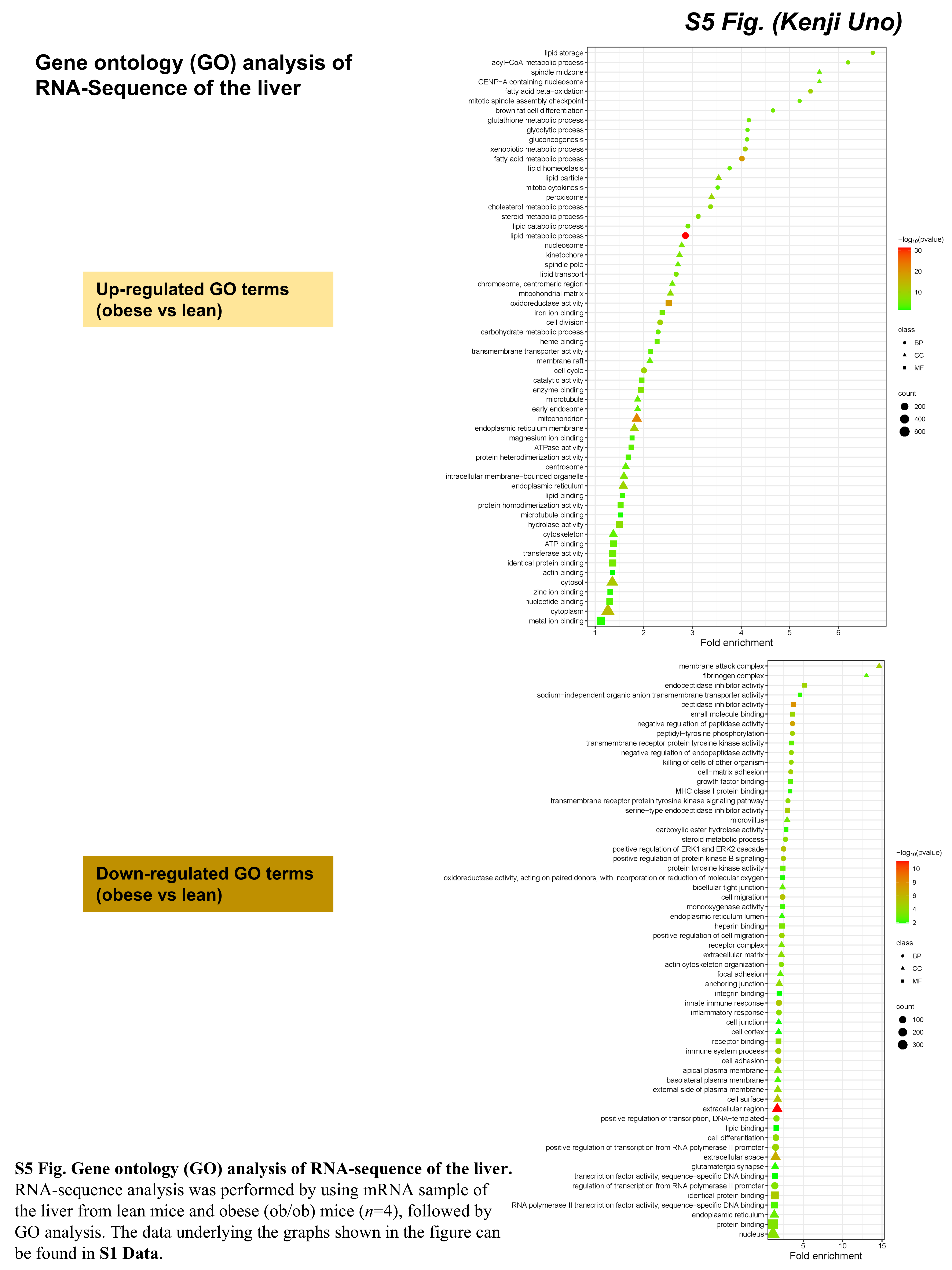

Supplement: S5 Fig — RNA-sequence analysis was performed by using mRNA sample of the liver from lean mice and obese (ob/ob) mice (n = 4), followed by GO analysis. The data underlying the graphs shown in the figure can be found in S1 Data. (TIF) [file pbio.3002955.s005.tif]

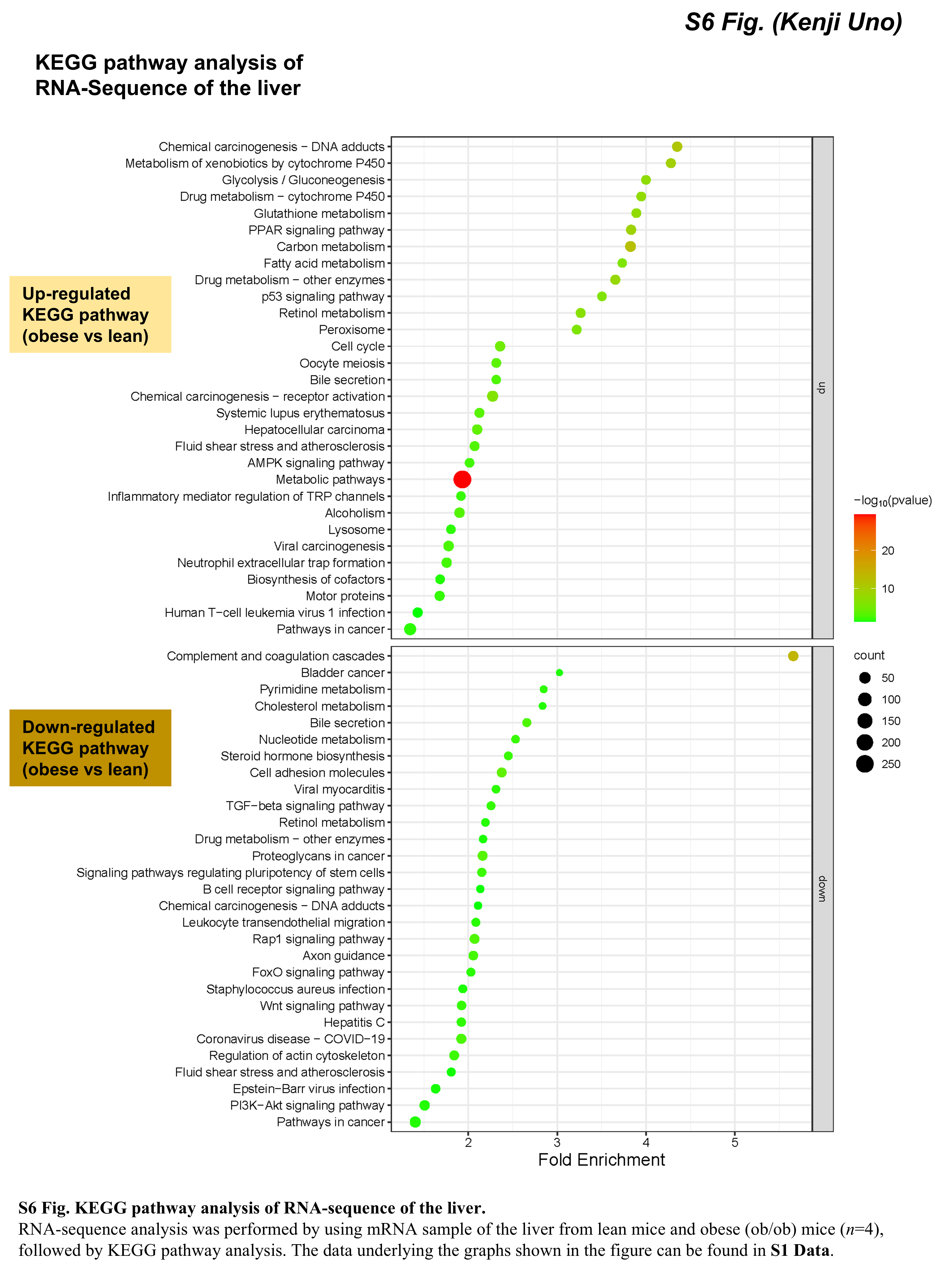

Supplement: S6 Fig — RNA-sequence analysis was performed by using mRNA sample of the liver from lean mice and obese (ob/ob) mice (n = 4), followed by KEGG pathway analysis. The data underlying the graphs shown in the figure can be found in S1 Data. (TIF) [file pbio.3002955.s006.tif]

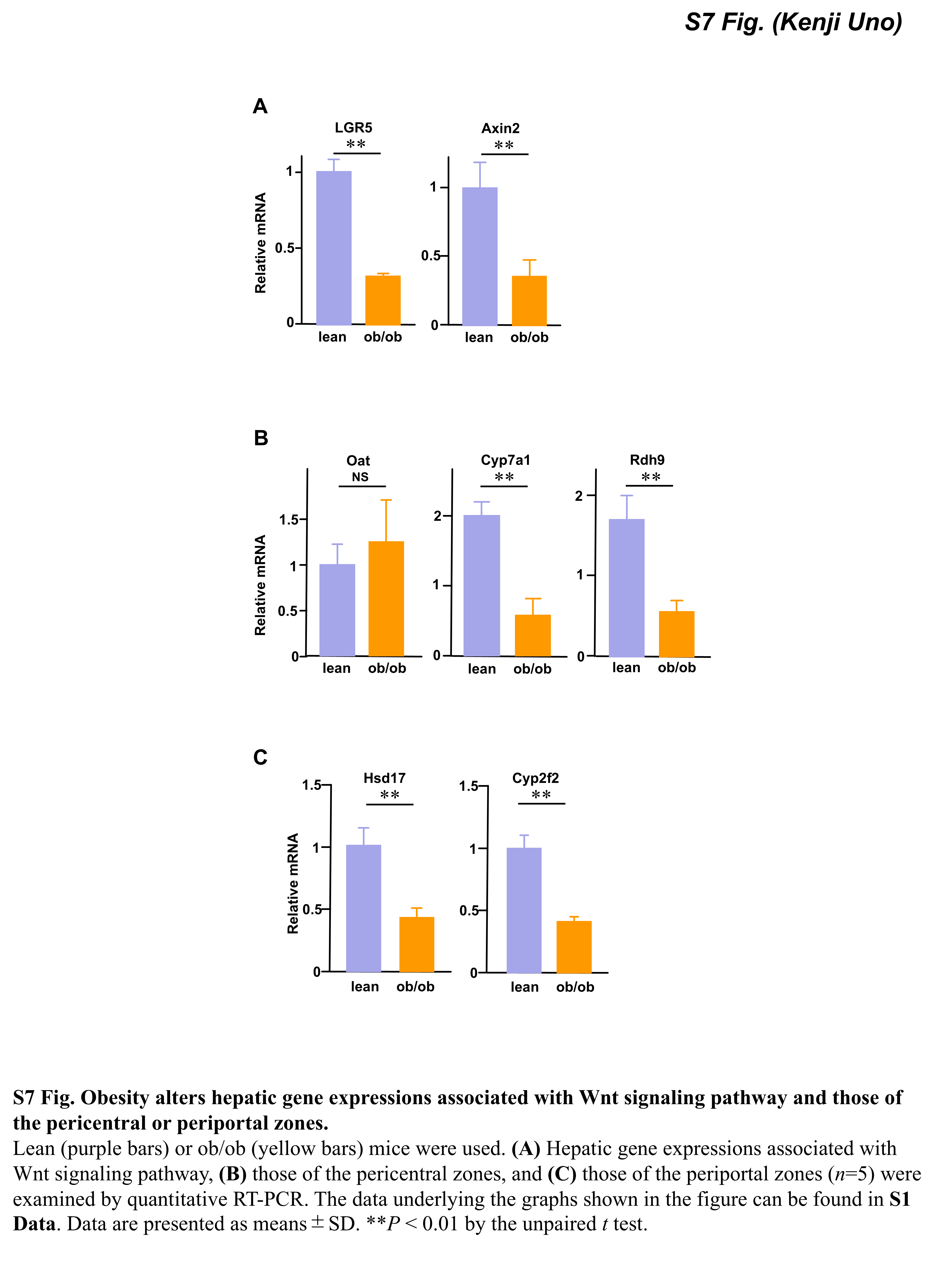

Supplement: S7 Fig — Lean (purple bars) or ob/ob (yellow bars) mice were used. (A) Hepatic gene expressions associated with Wnt signaling pathway, (B) those of the pericentral zones, and (C) those of the periportal zones (n = 5) were examined by quantitative RT-PCR. The data underlying the graphs shown in the figure can be found in S1 Data. Data are presented as means ± SD. **P < 0.01 by the unpaired t test. (TIF) [file pbio.3002955.s007.tif]

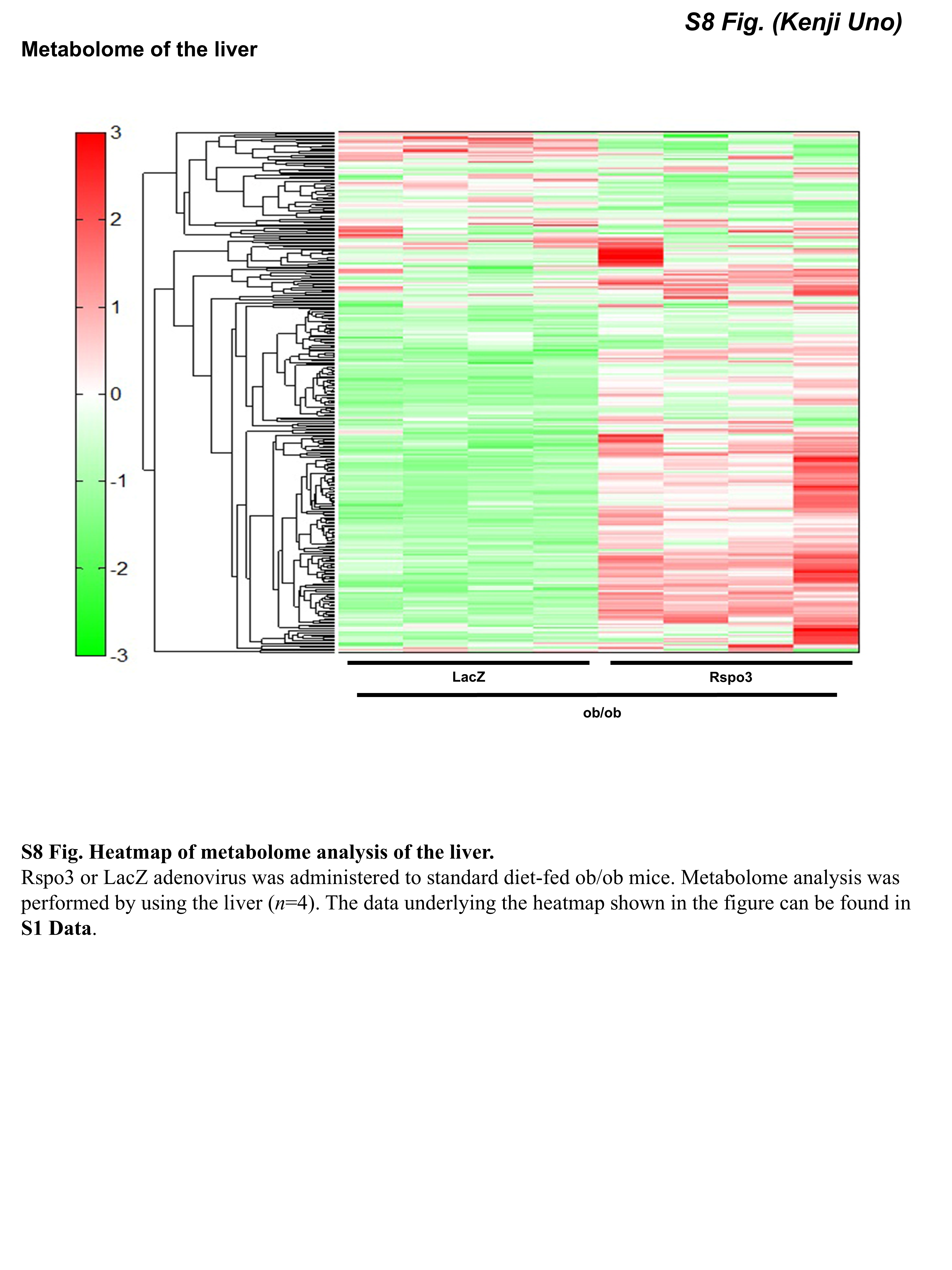

Supplement: S8 Fig — Rspo3 or LacZ adenovirus was administered to standard diet-fed ob/ob mice. Metabolome analysis was performed by using the liver (n = 4). The data underlying the heatmap shown in the figure can be found in S1 Data. (TIF) [file pbio.3002955.s008.tif]

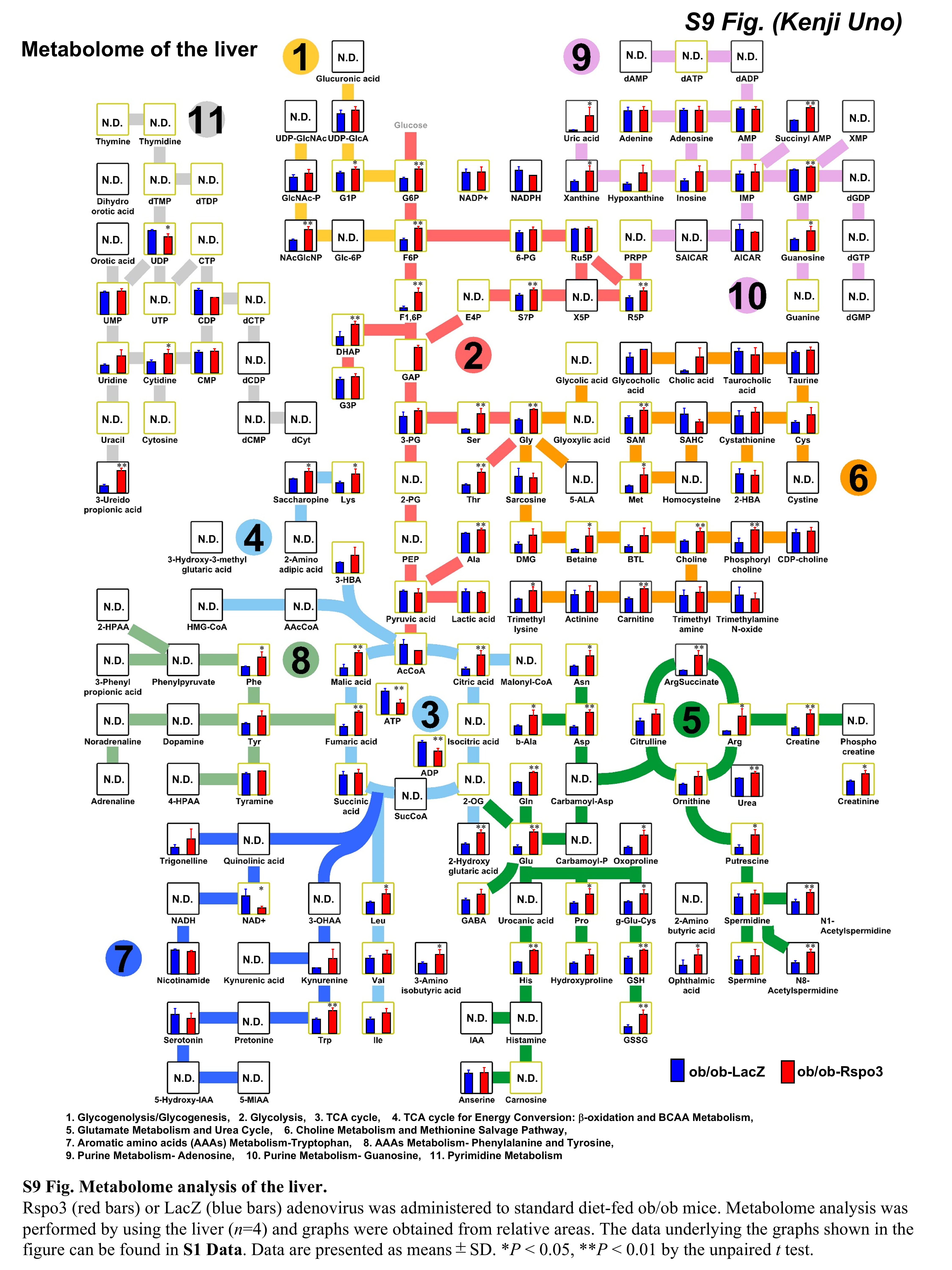

Supplement: S9 Fig — Rspo3 (red bars) or LacZ (blue bars) adenovirus was administered to standard diet-fed ob/ob mice. Metabolome analysis was performed by using the liver (n = 4) and graphs were obtained from relative areas. The data underlying the graphs shown in the figure can be found in S1 Data. Data are presented as means ± SD. *P < 0.05, **P < 0.01 by the unpaired t test. (TIF) [file pbio.3002955.s009.tif]

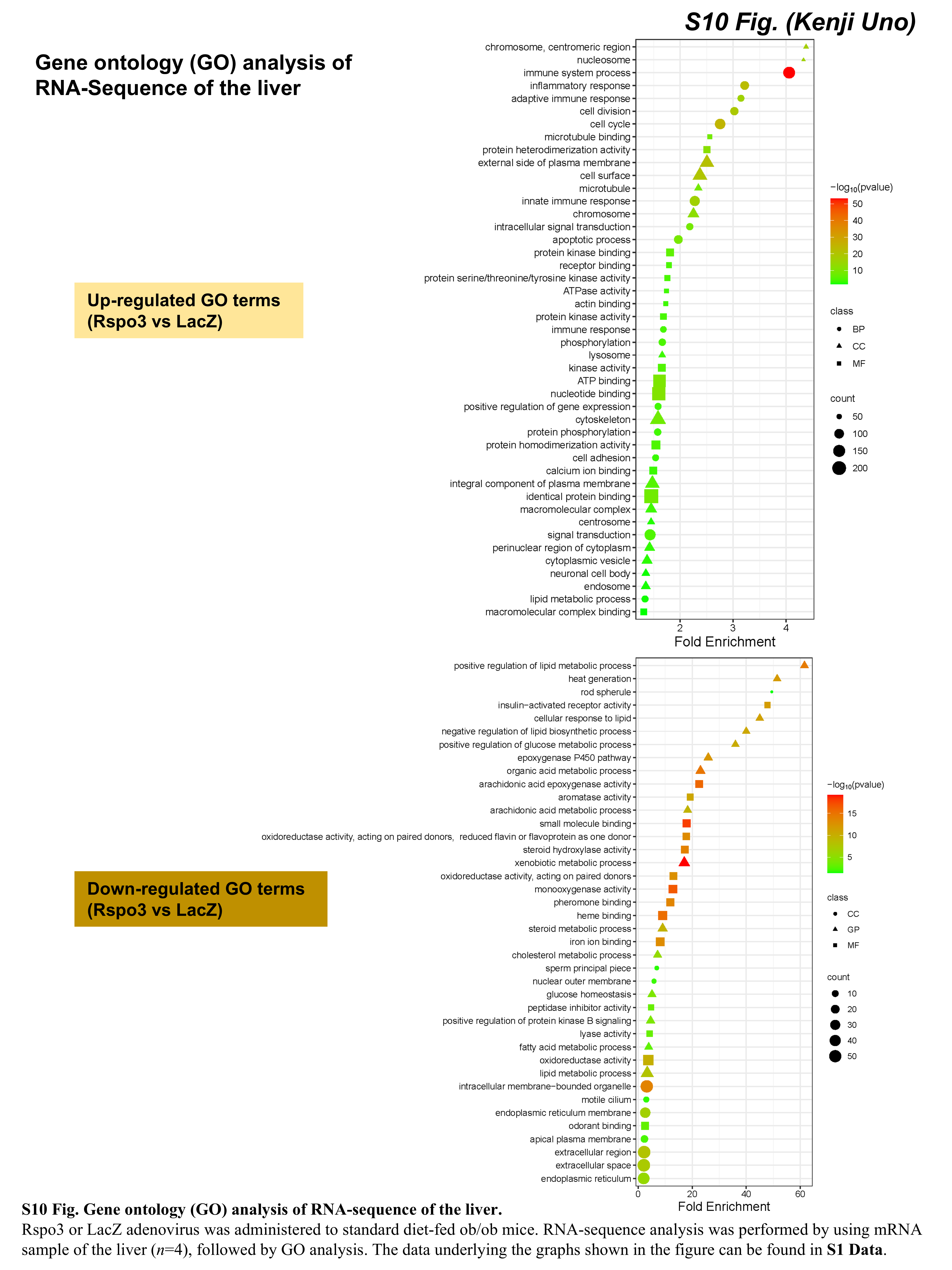

Supplement: S10 Fig — Rspo3 or LacZ adenovirus was administered to standard diet-fed ob/ob mice. RNA-sequence analysis was performed by using mRNA sample of the liver (n = 4), followed by GO analysis. The data underlying the graphs shown in the figure can be found in S1 Data. (TIF) [file pbio.3002955.s010.tif]

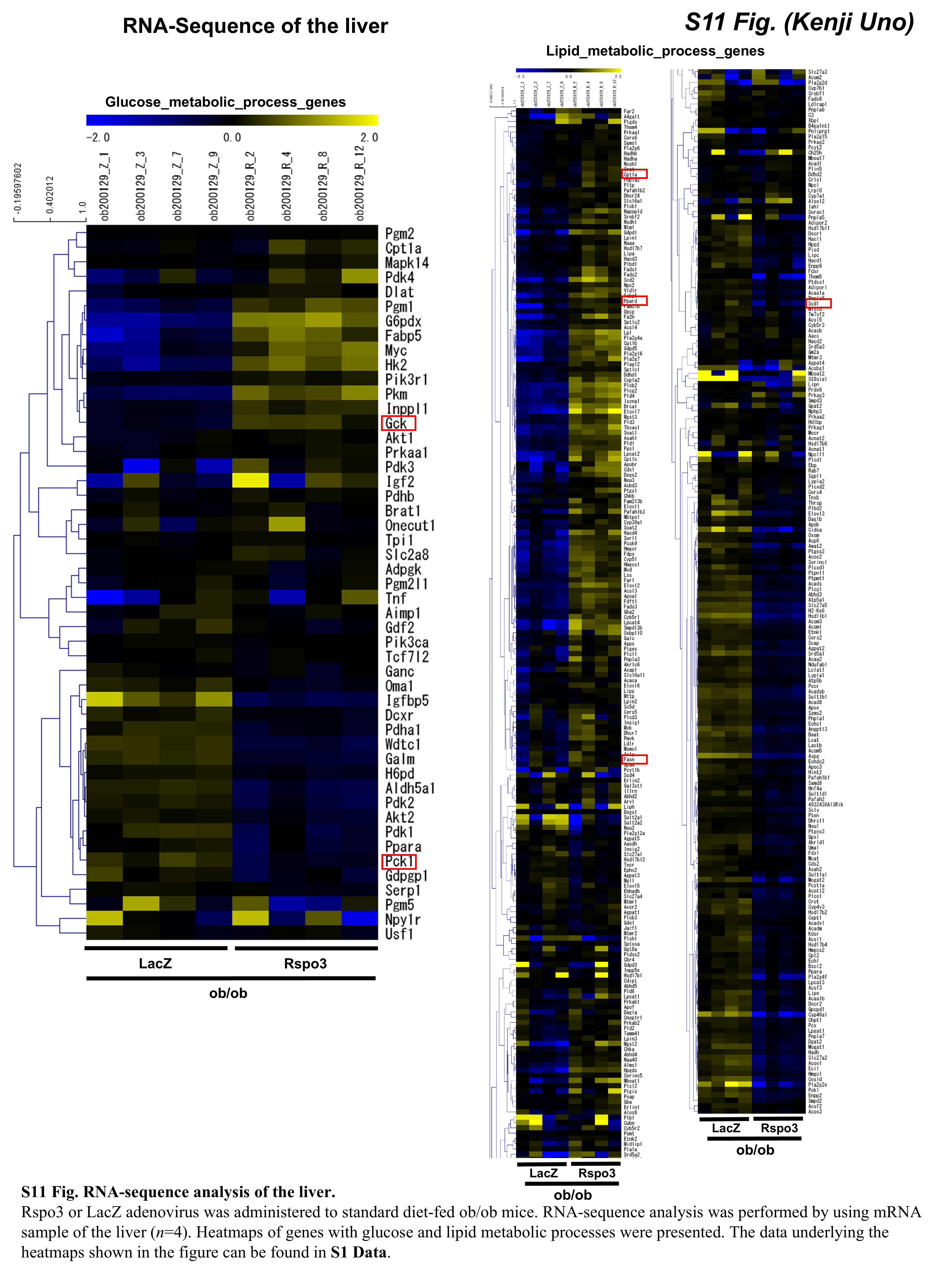

Supplement: S11 Fig — Rspo3 or LacZ adenovirus was administered to standard diet-fed ob/ob mice. RNA-sequence analysis was performed by using mRNA sample of the liver (n = 4). Heatmaps of genes with glucose and lipid metabolic processes were presented. The data underlying the heatmaps shown in the figure can be found in S1 Data. (TIF) [file pbio.3002955.s011.tif]

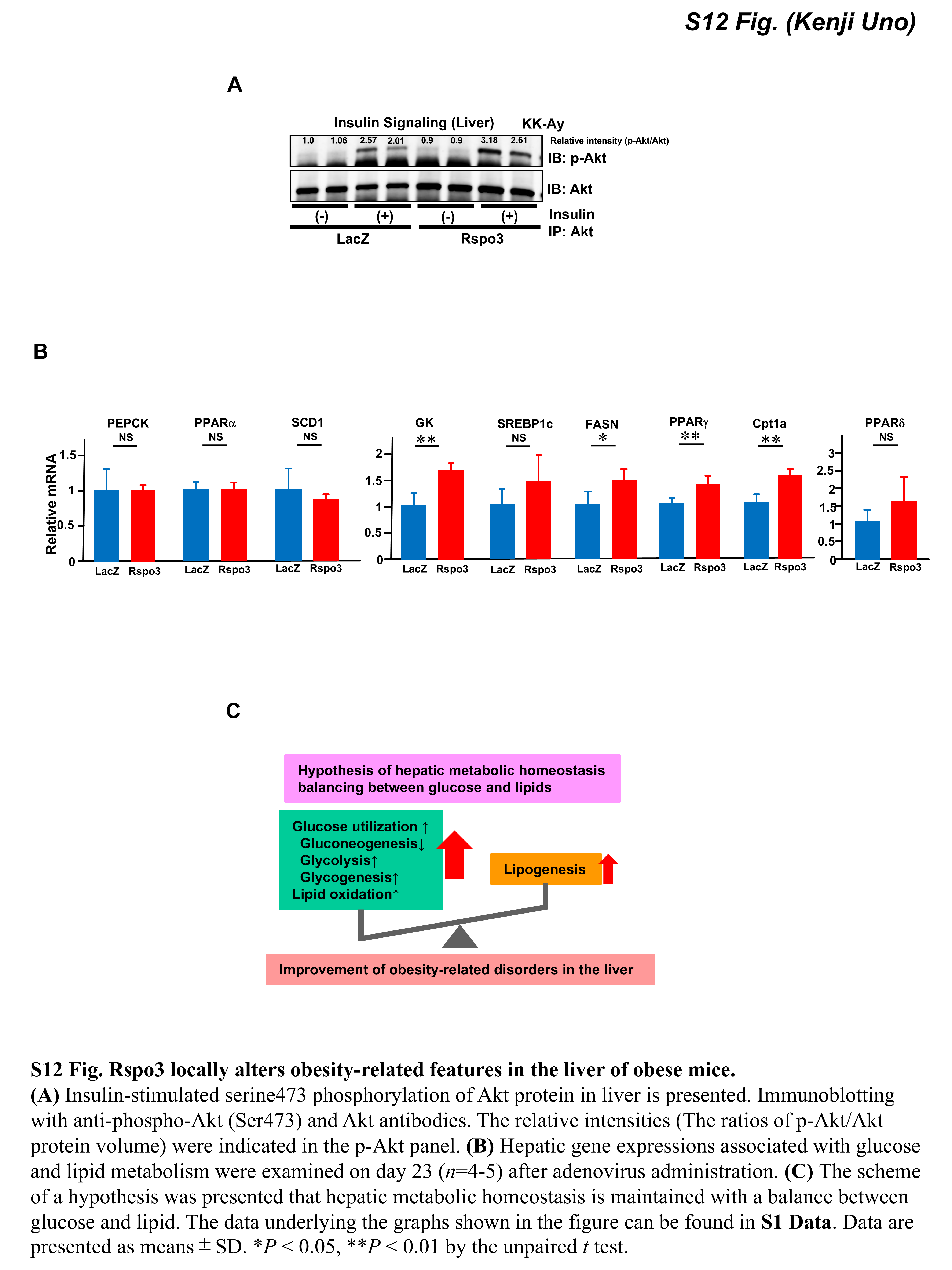

Supplement: S12 Fig — (A) Insulin-stimulated serine473 phosphorylation of Akt protein in liver is presented. Immunoblotting with anti-phospho-Akt (Ser473) and Akt antibodies. The relative intensities (the ratios of p-Akt/Akt protein volume) were indicated in the p-Akt panel. (B) Hepatic gene expressions associated with glucose and lipid metabolism were examined on day 23 (n = 4–5) after adenovirus administration. (C) The scheme of a hypothesis was presented that hepatic metabolic homeostasis is maintained with a balance between glucose and lipid. The data underlying the graphs shown in the figure can be found in S1 Data. Data are presented as means ± SD. *P < 0.05, **P < 0.01 by the unpaired t test. (TIF) [file pbio.3002955.s012.tif]

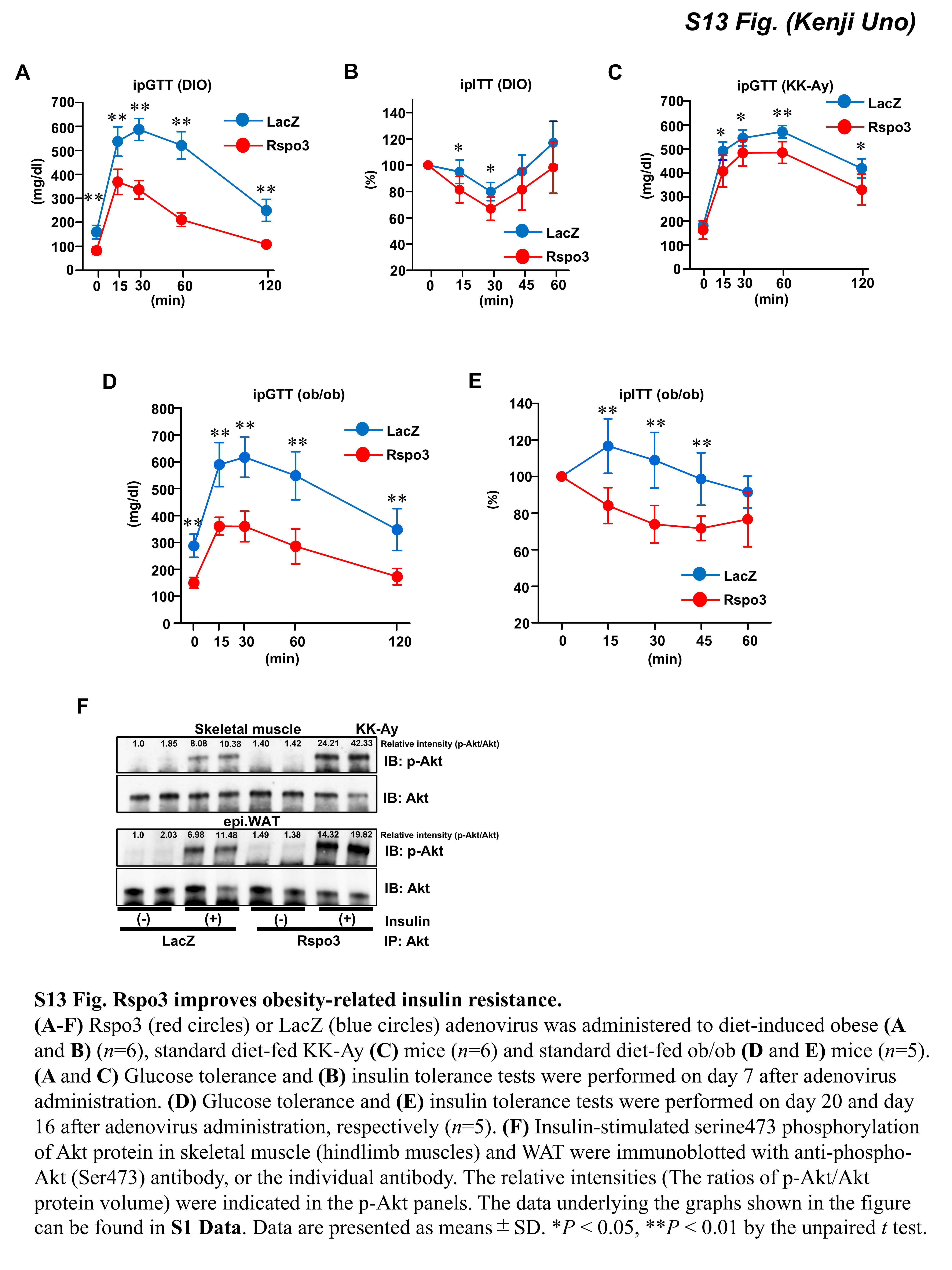

Supplement: S13 Fig — (A–F) Rspo3 (red circles) or LacZ (blue circles) adenovirus was administered to diet-induced obese (A and B) (n = 6), standard diet-fed KK-Ay (C) mice (n = 6) and standard diet-fed ob/ob (D and E) mice (n = 5). (A and C) Glucose tolerance and (B) insulin tolerance tests were performed on day 7 after adenovirus administration. (D) Glucose tolerance and (E) insulin tolerance tests were performed on day 20 and day 16 after adenovirus administration, respectively (n = 5). (F) Insulin-stimulated serine473 phosphorylation of Akt protein in skeletal muscle (hindlimb muscles) and WAT were immunoblotted with anti-phospho-Akt (Ser473) antibody, or the individual antibody. The relative intensities (the ratios of p-Akt/Akt protein volume) were indicated in the p-Akt panels. The data underlying the graphs shown in the figure can be found in S1 Data. Data are presented as means ± SD. *P < 0.05, **P < 0.01 by the unpaired t test. (TIF) [file pbio.3002955.s013.tif]

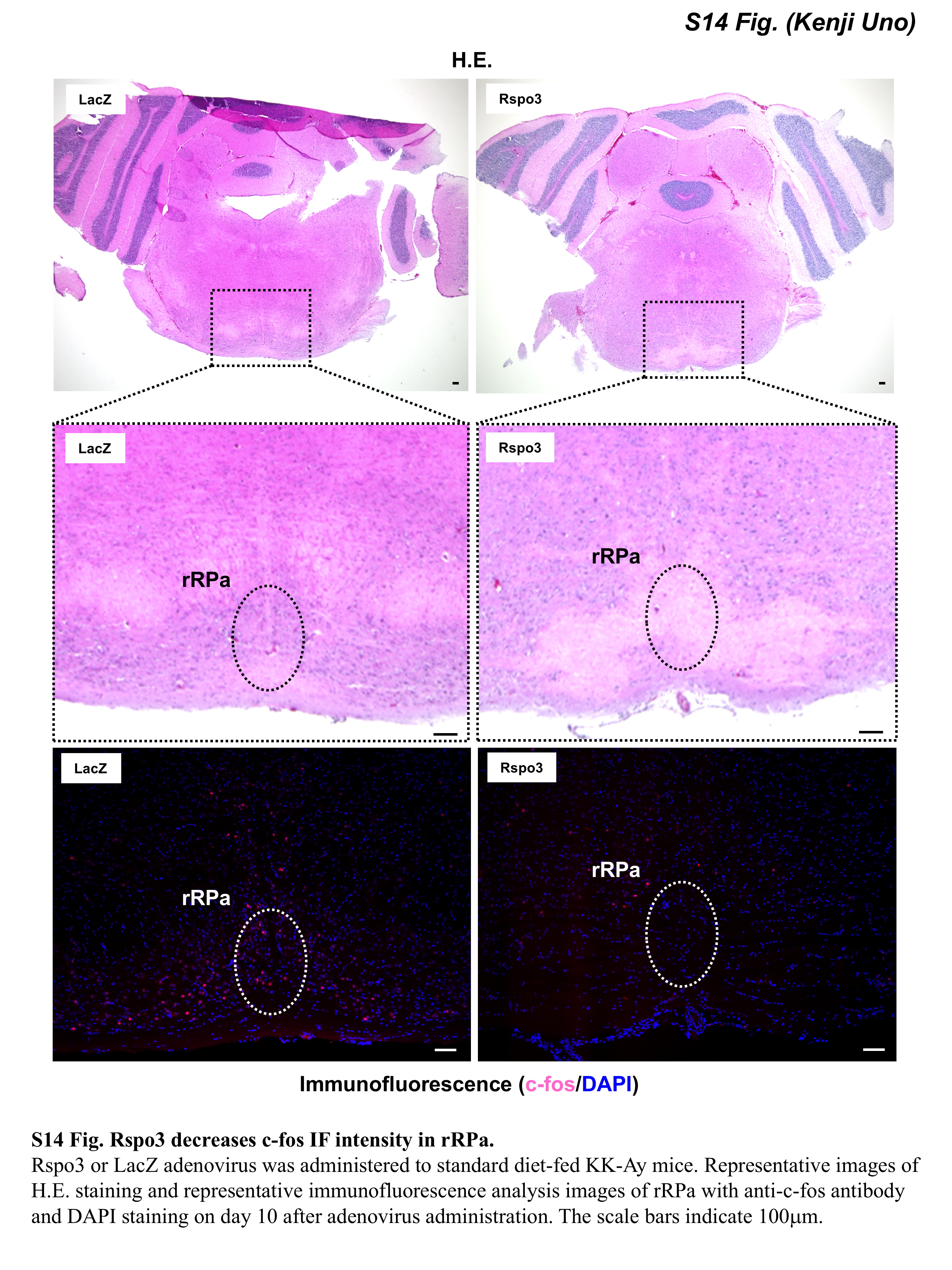

Supplement: S14 Fig — Rspo3 or LacZ adenovirus was administered to standard diet-fed KK-Ay mice. Representative images of HE staining and representative immunofluorescence analysis images of rRPa with anti-c-fos antibody and DAPI staining on day 10 after adenovirus administration. The scale bars indicate 100 μm. (TIF) [file pbio.3002955.s014.tif]

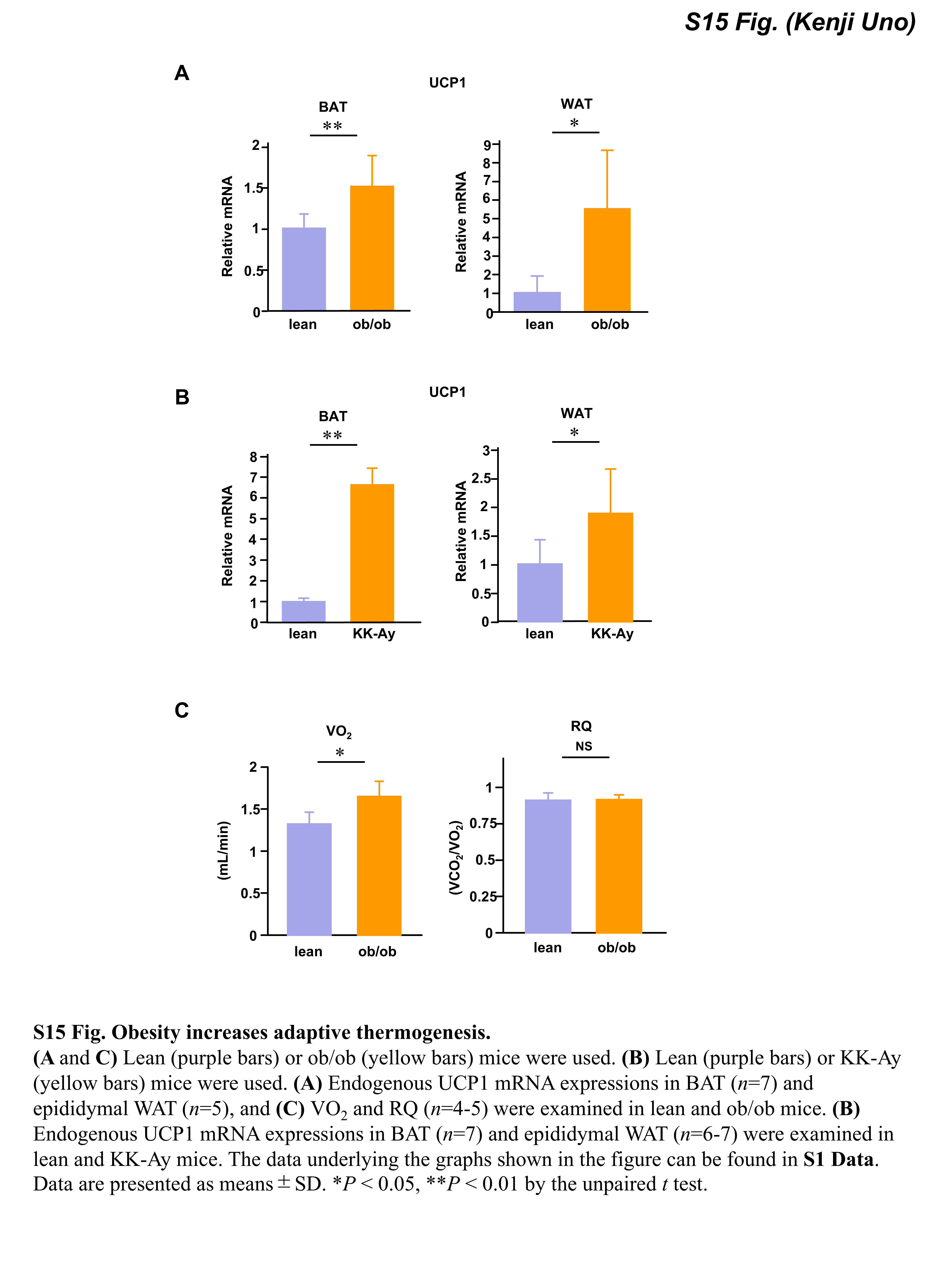

Supplement: S15 Fig — (A and C) Lean (purple bars) or ob/ob (yellow bars) mice were used. (B) Lean (purple bars) or KK-Ay (yellow bars) mice were used. (A) Endogenous UCP1 mRNA expressions in BAT (n = 7) and epididymal WAT (n = 5), and (C) VO2 and RQ (n = 4–5) were examined in lean and ob/ob mice. (B) Endogenous UCP1 mRNA expressions in BAT (n = 7) and epididymal WAT (n = 6–7) were examined in lean and KK-Ay mice. The data underlying the graphs shown in the figure can be found in S1 Data. Data are presented as means ± SD. *P < 0.05, **P < 0.01 by the unpaired t test. (TIF) [file pbio.3002955.s015.tif]

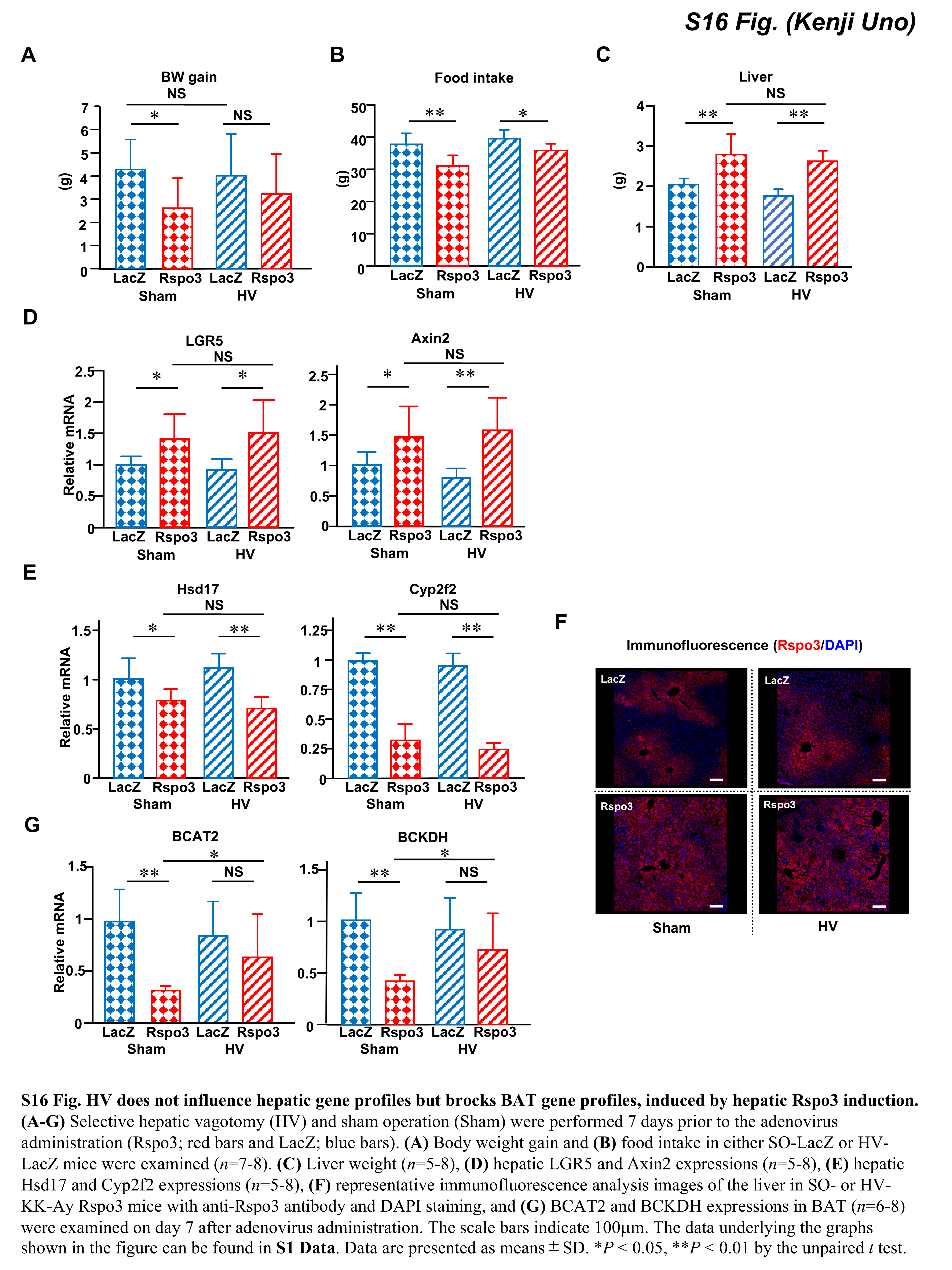

Supplement: S16 Fig — (A–G) Selective hepatic vagotomy (HV) and sham operation (Sham) were performed 7 days prior to the adenovirus administration (Rspo3; red bars and LacZ; blue bars). (A) Body weight gain and (B) food intake in either SO-LacZ or HV-LacZ mice were examined (n = 7–8). (C) Liver weight (n = 5–8), (D) hepatic LGR5 and Axin2 expressions (n = 5–8), (E) hepatic Hsd17 and Cyp2f2 expressions (n = 5–8), (F) representative immunofluorescence analysis images of the liver in SO- or HV-KK-Ay Rspo3 mice with anti-Rspo3 antibody and DAPI staining, and (G) BCAT2 and BCKDH expressions in BAT (n = 6–8) were examined on day 7 after adenovirus administration. The scale bars indicate 100 μm. The data underlying the graphs shown in the figure can be found in S1 Data. Data are presented as means ± SD. *P < 0.05, **P < 0.01 by the unpaired t test. (TIF) [file pbio.3002955.s016.tif]

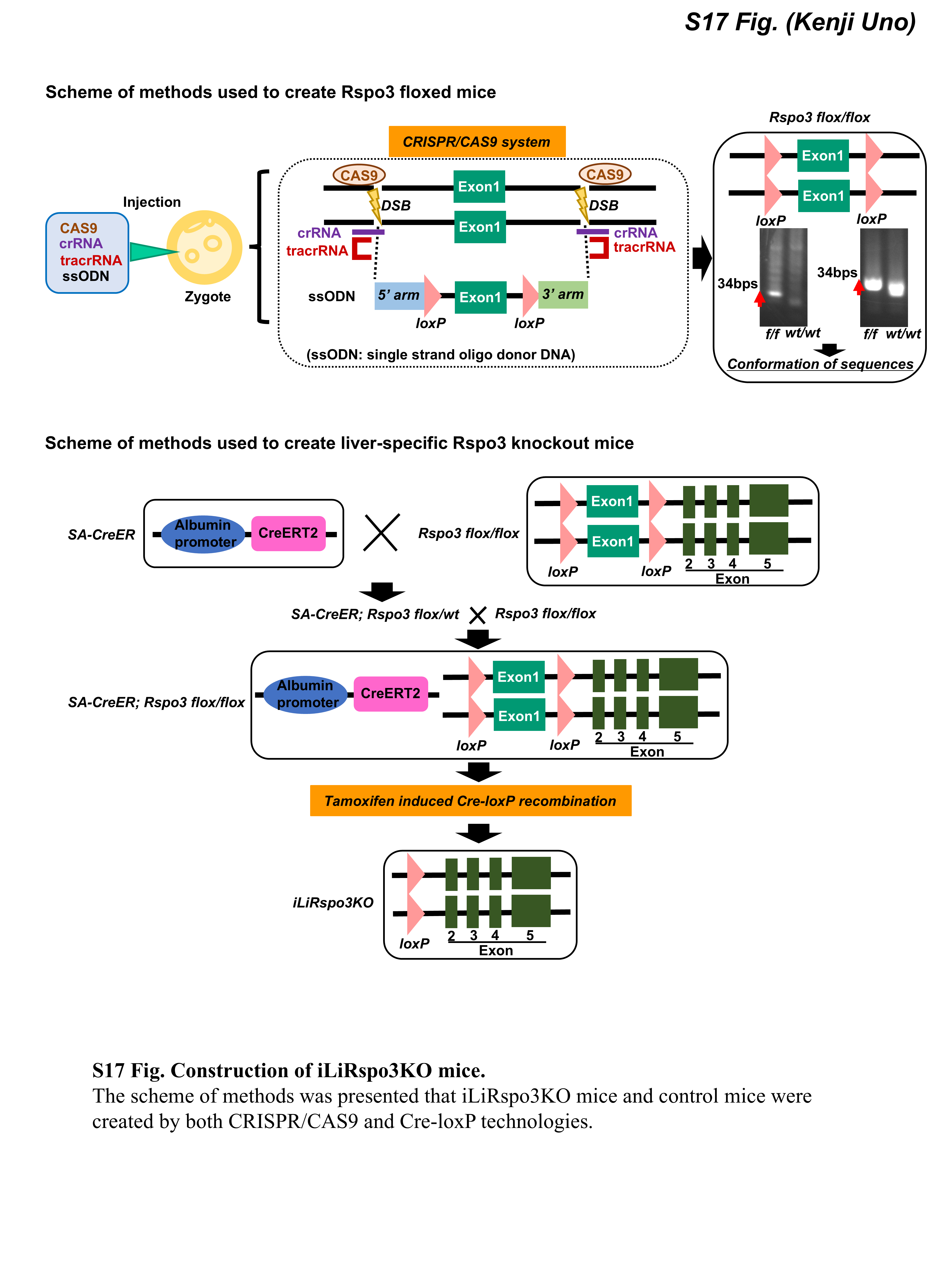

Supplement: S17 Fig — The scheme of methods was presented that iLiRspo3KO mice and control mice were created by both CRISPR/CAS9 and Cre-loxP technologies. (TIF) [file pbio.3002955.s017.tif]

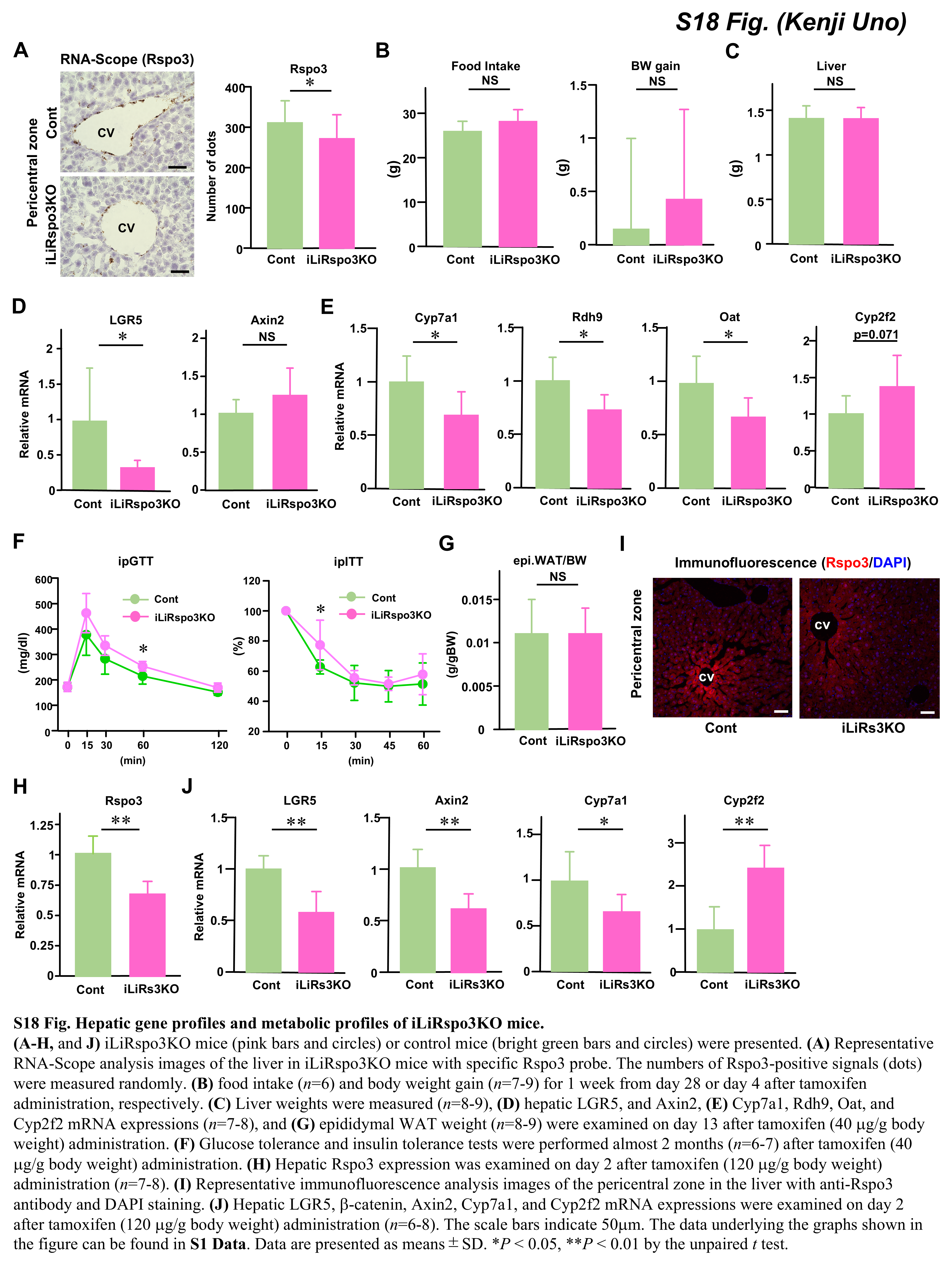

Supplement: S18 Fig — (A–H and J) iLiRspo3KO mice (pink bars and circles) or control mice (bright green bars and circles) were presented. (A) Representative RNA-Scope analysis images of the liver in iLiRspo3KO mice with specific Rspo3 probe. The numbers of Rspo3-positive signals (dots) were measured randomly. (B) Food intake (n = 6) and body weight gain (n = 7–9) for 1 week from day 28 or day 4 after tamoxifen administration, respectively. (C) Liver weights were measured (n = 8–9), (D) hepatic LGR5, and Axin2, (E) Cyp7a1, Rdh9, Oat, and Cyp2f2 mRNA expressions (n = 7–8), and (G) epididymal WAT weight (n = 8–9) were examined on day 13 after tamoxifen (40 μg/g body weight) administration. (F) Glucose tolerance and insulin tolerance tests were performed almost 2 months (n = 6–7) after tamoxifen (40 μg/g body weight) administration. (H) Hepatic Rspo3 expression was examined on day 2 after tamoxifen (120 μg/g body weight) administration (n = 7–8). (I) Representative immunofluorescence analysis images of the pericentral zone in the liver with anti-Rspo3 antibody and DAPI staining. (J) Hepatic LGR5, β-catenin, Axin2, Cyp7a1, and Cyp2f2 mRNA expressions were examined on day 2 after tamoxifen (120 μg/g body weight) administration (n = 6–8). The scale bars indicate 50 μm. The data underlying the graphs shown in the figure can be found in S1 Data. Data are presented as means ± SD. *P < 0.05, **P < 0.01 by the unpaired t test. (TIF) [file pbio.3002955.s018.tif]

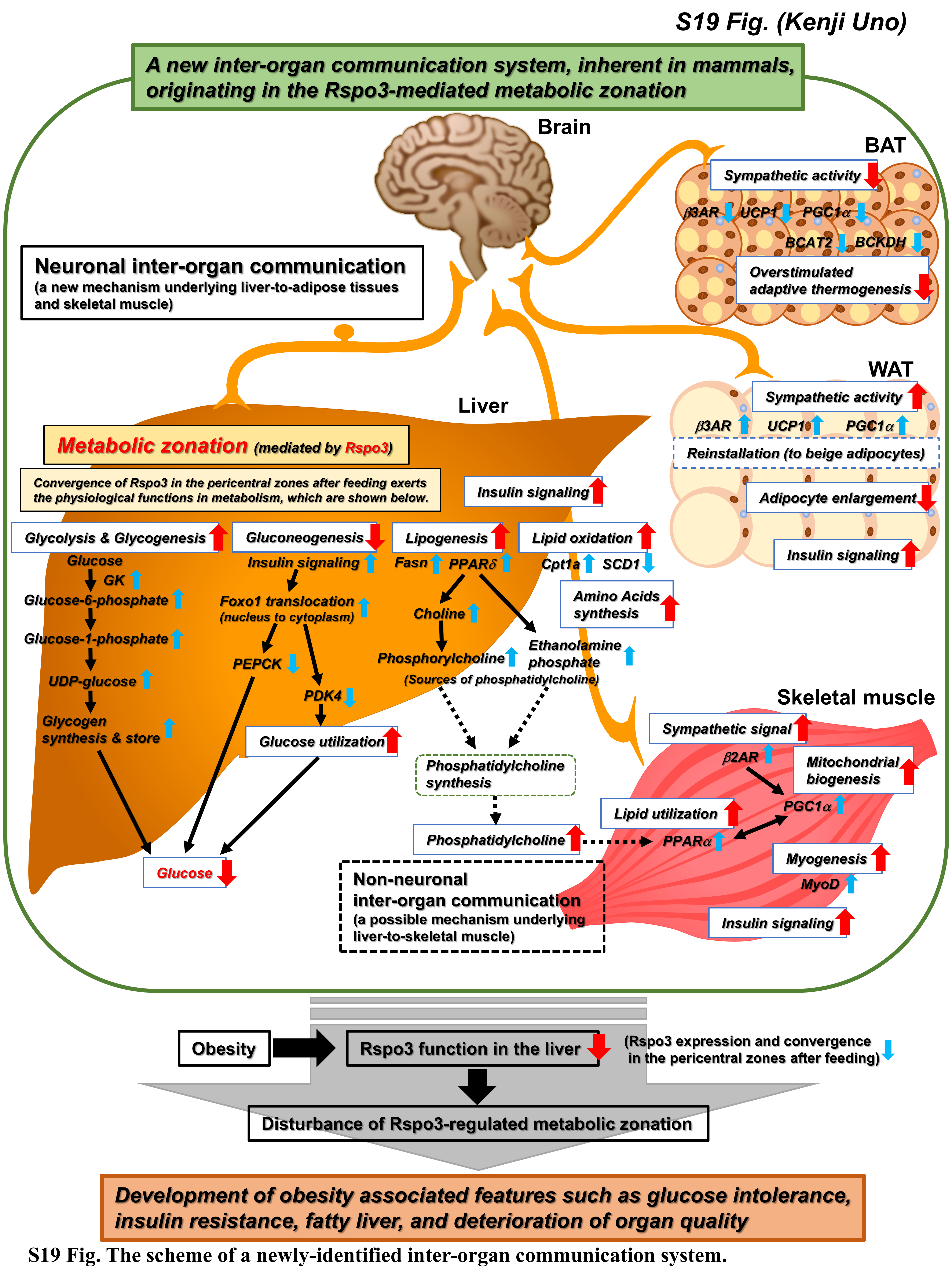

Supplement: S19 Fig — (TIF) [file pbio.3002955.s019.tif]

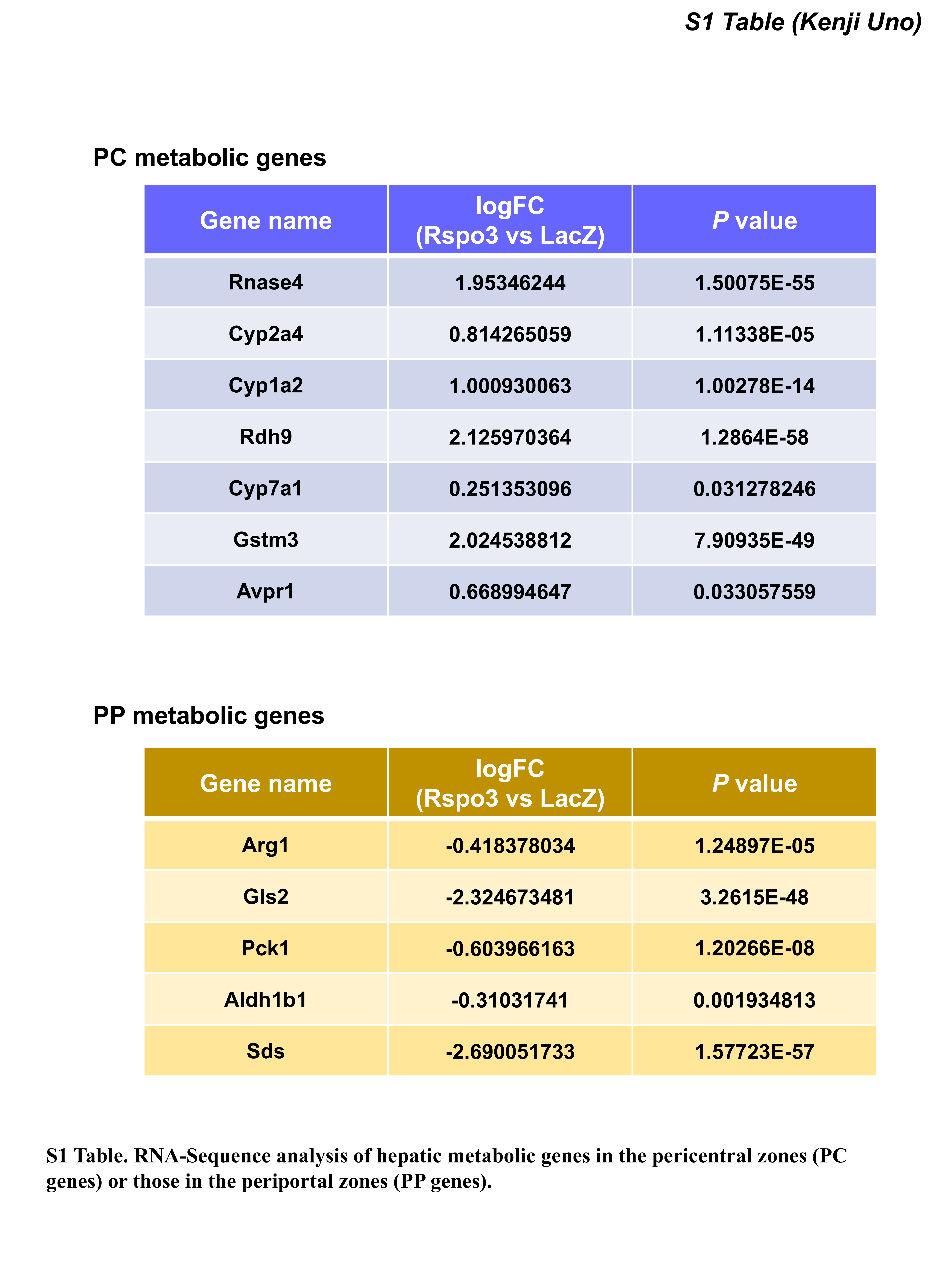

Supplement: S1 Table — (TIF) [file pbio.3002955.s020.tif]

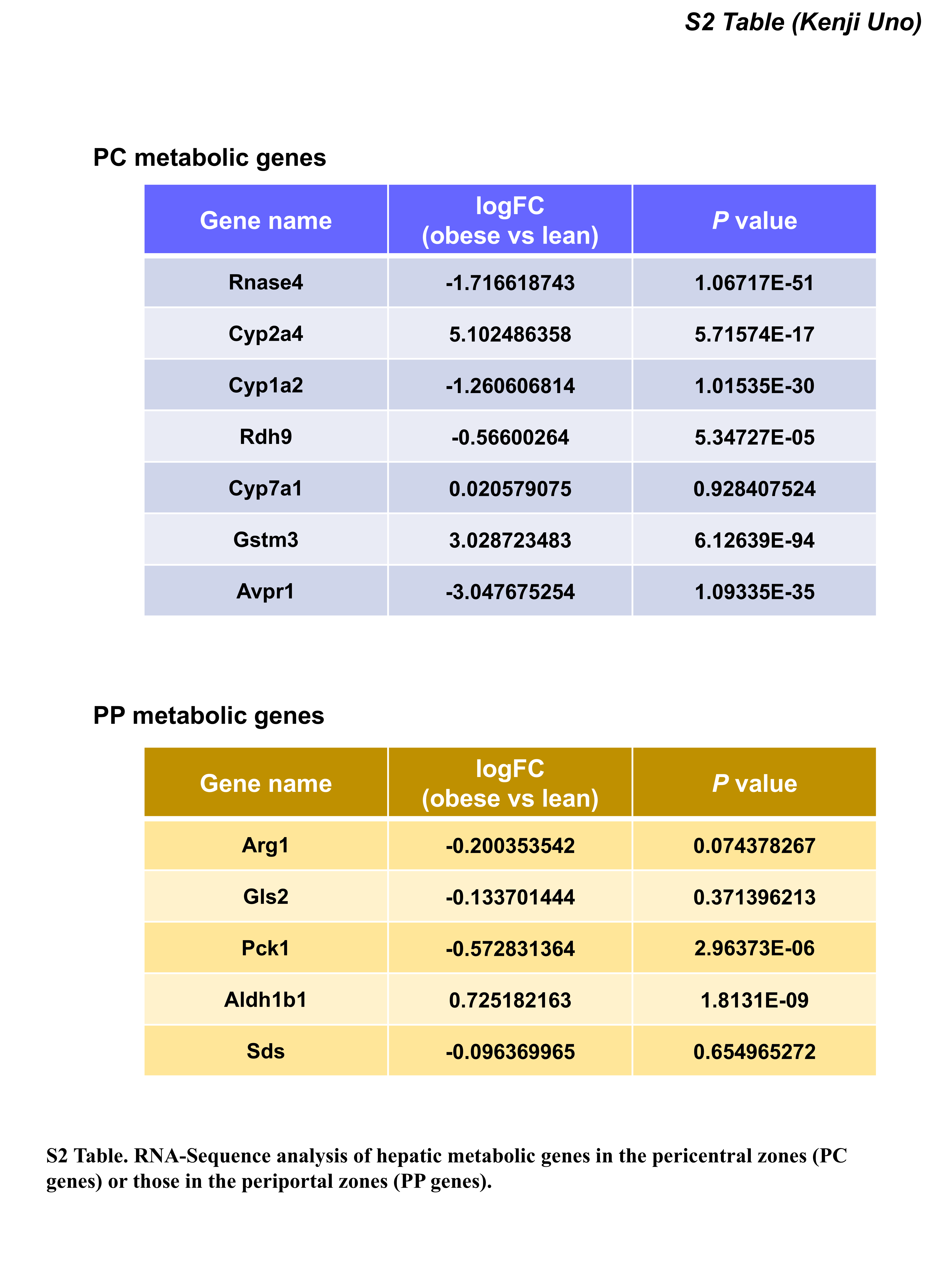

Supplement: S2 Table — (TIF) [file pbio.3002955.s021.tif]
