# Supplementary material for: Rspo3-mediated metabolic liver zonation regulates systemic glucose metabolism and body mass in mice
Source: PLoS Biol. 2025 Jan 24;23(1):e3002955. doi: 10.1371/journal.pbio.3002955 (PMC11759367; doi:10.1371/journal.pbio.3002955)
Supplement: S1 Raw Images — (PDF) [file pbio.3002955.s022.pdf]

Fig 1B

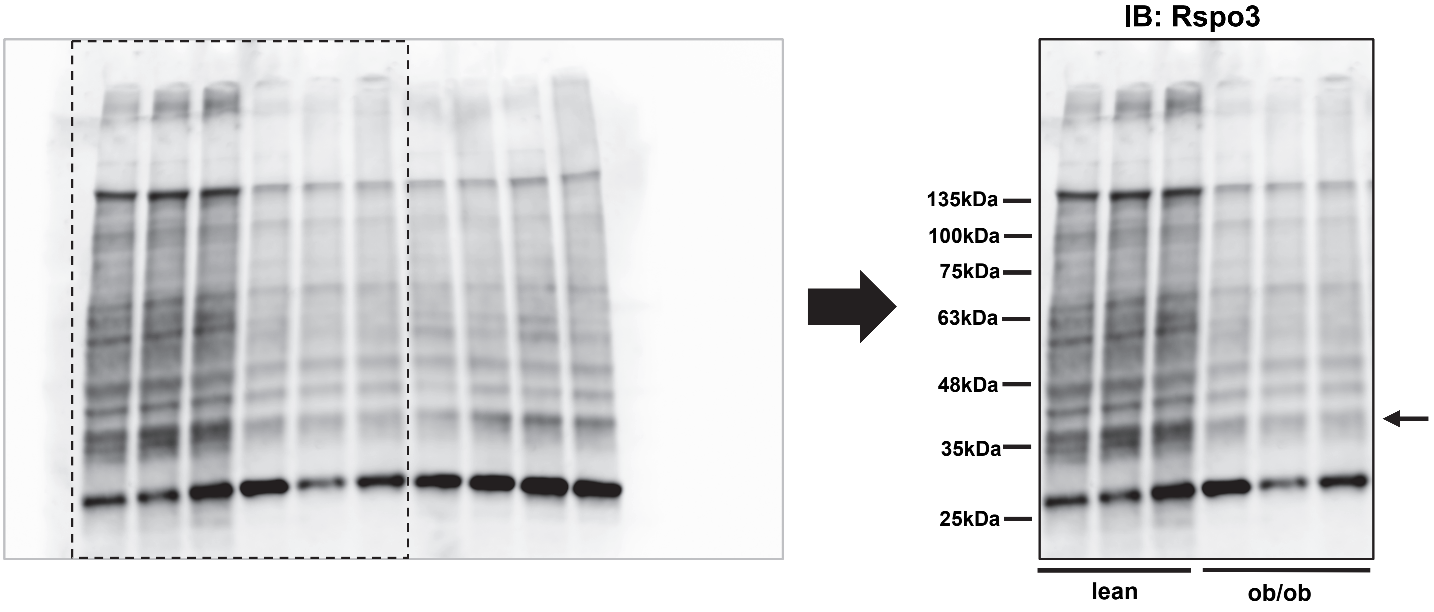

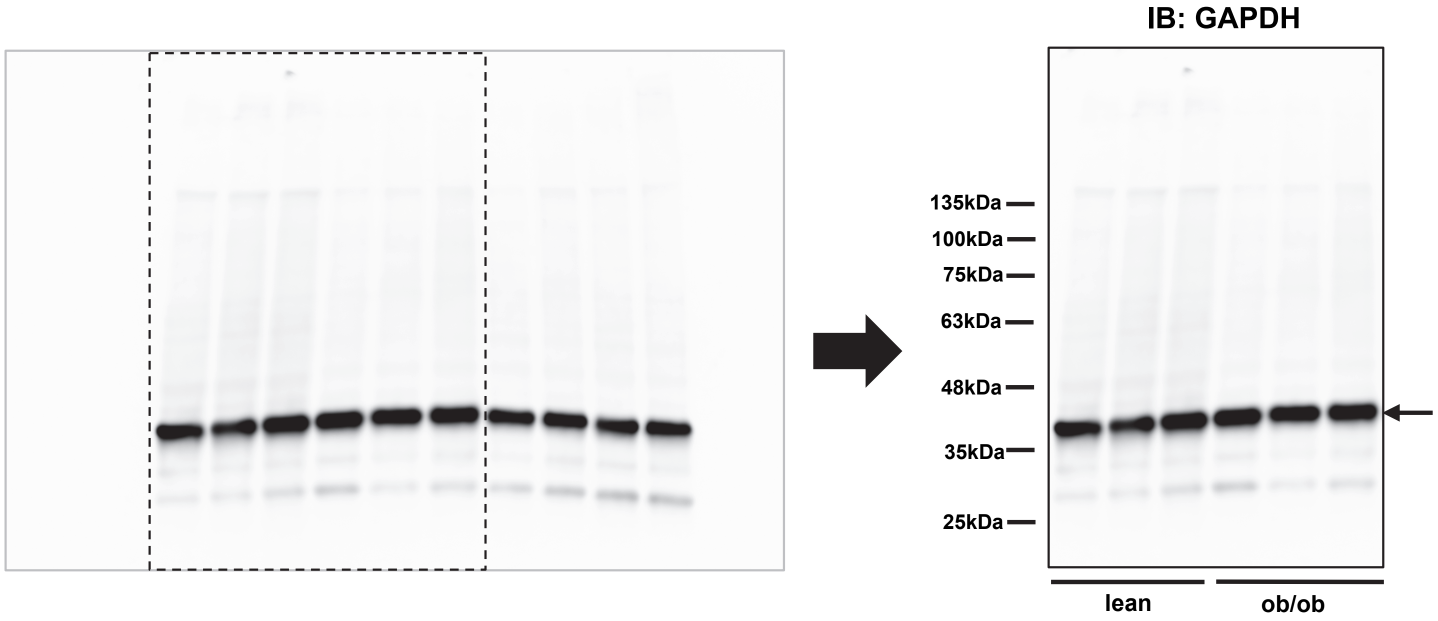

Supplemental Western Blot 1. Full western blots in Fig 1B

Fig 2A

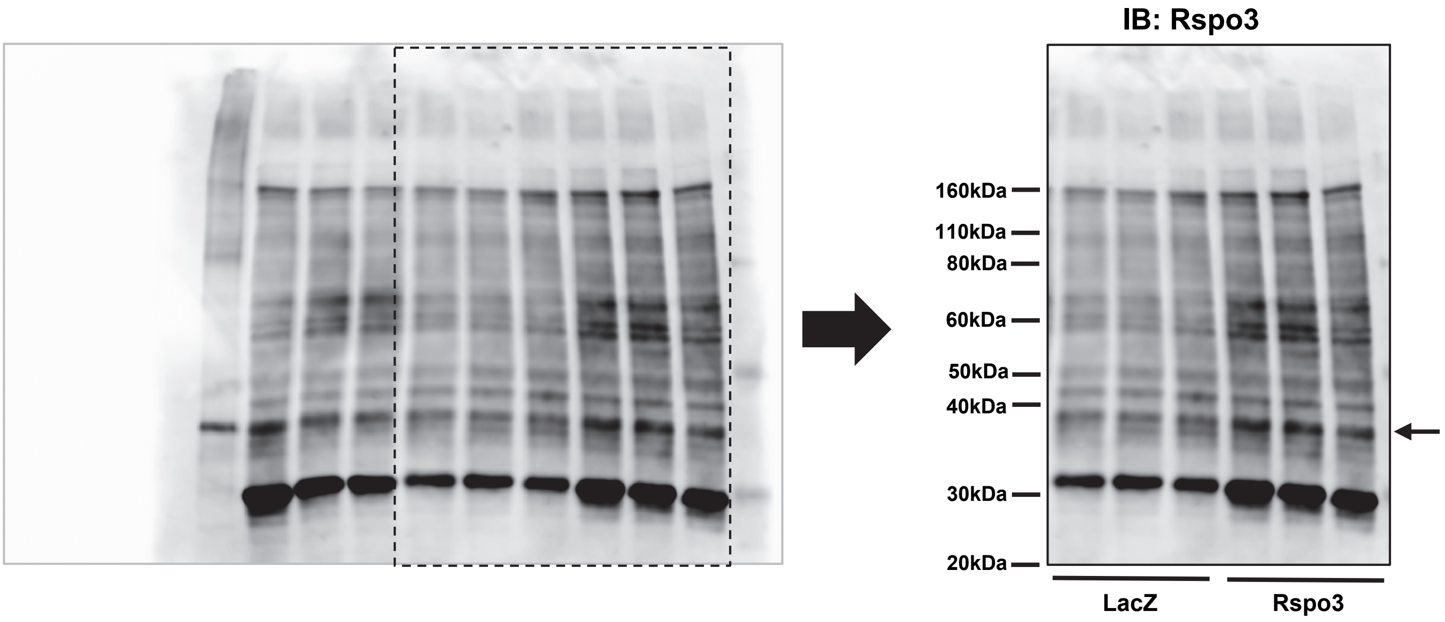

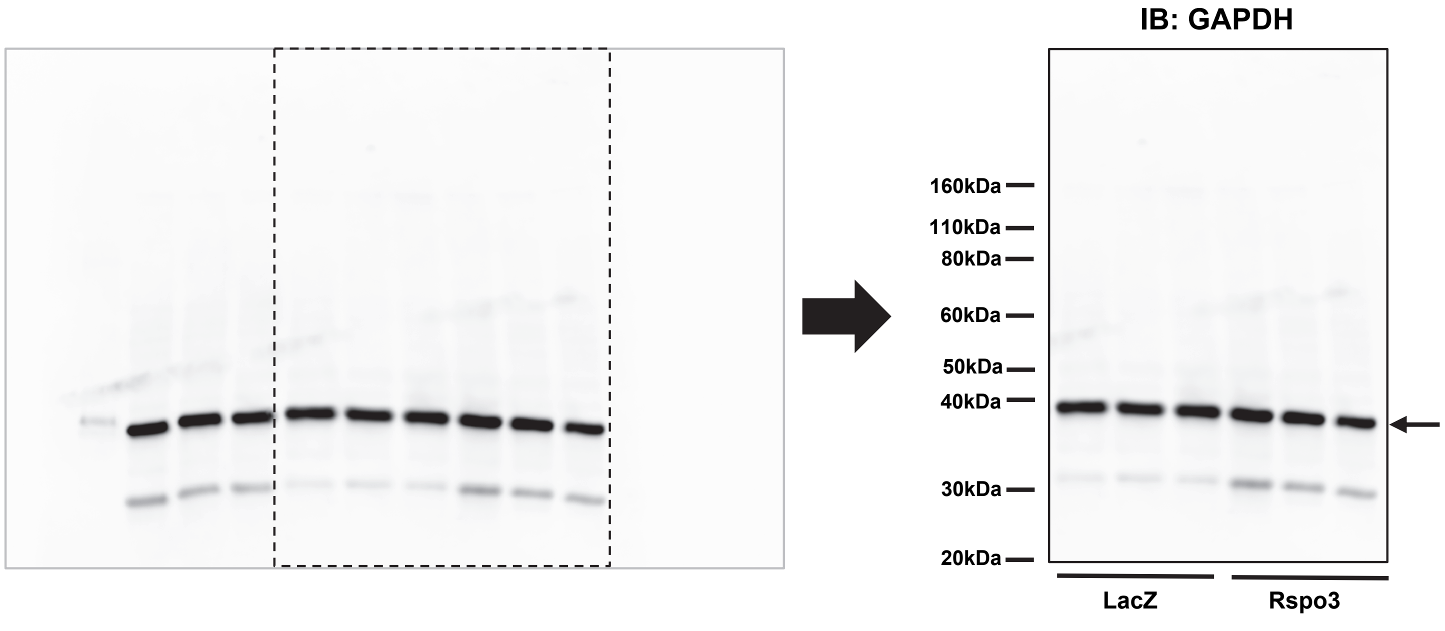

Supplemental Western Blot 2. Full western blots in Fig 2A

Fig 3A

SWB 3. (Kenji Uno)

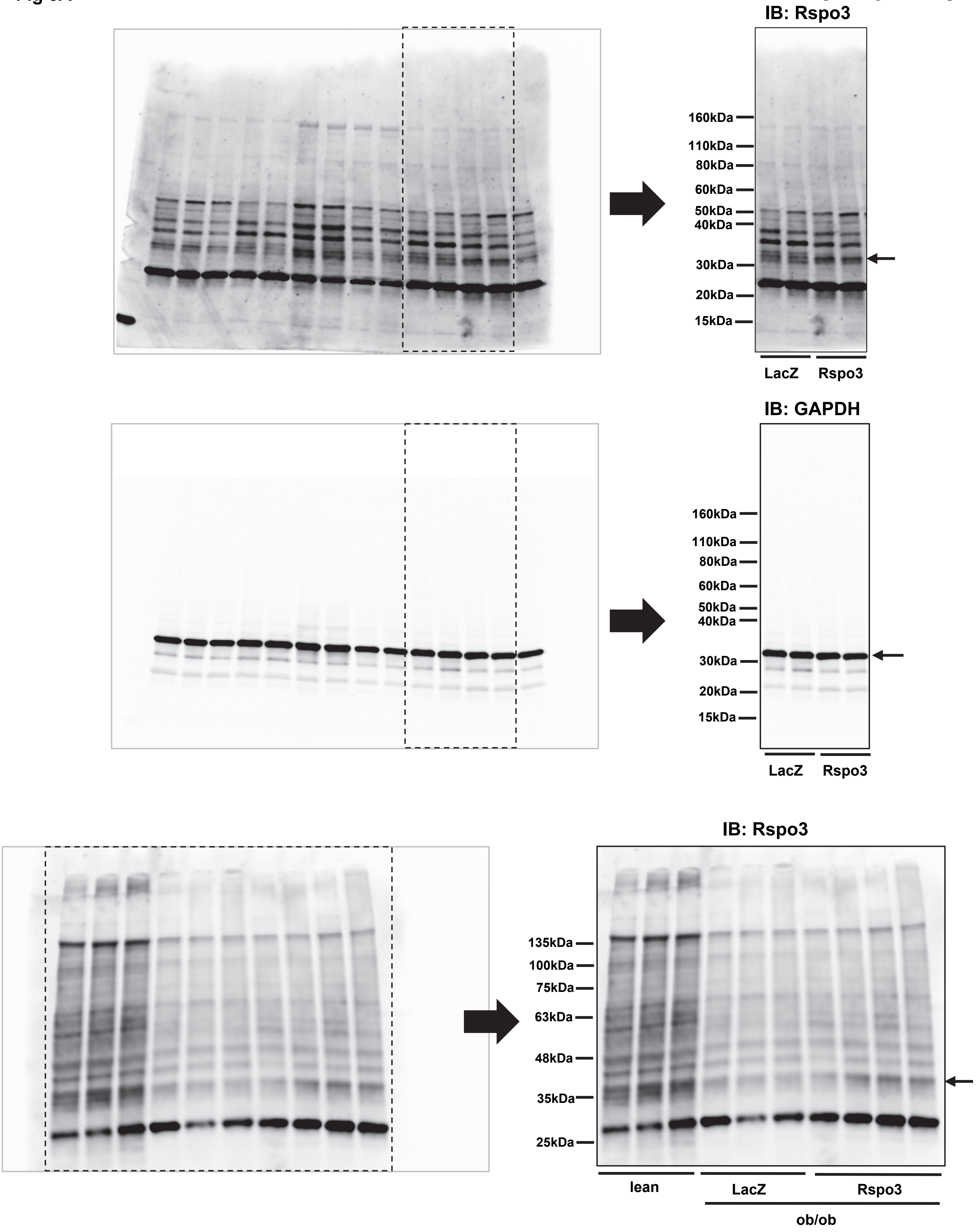

Supplemental Western Blot 3. Full western blots in Fig 3A

SWB 4. (Kenji Uno)

Fig 3A

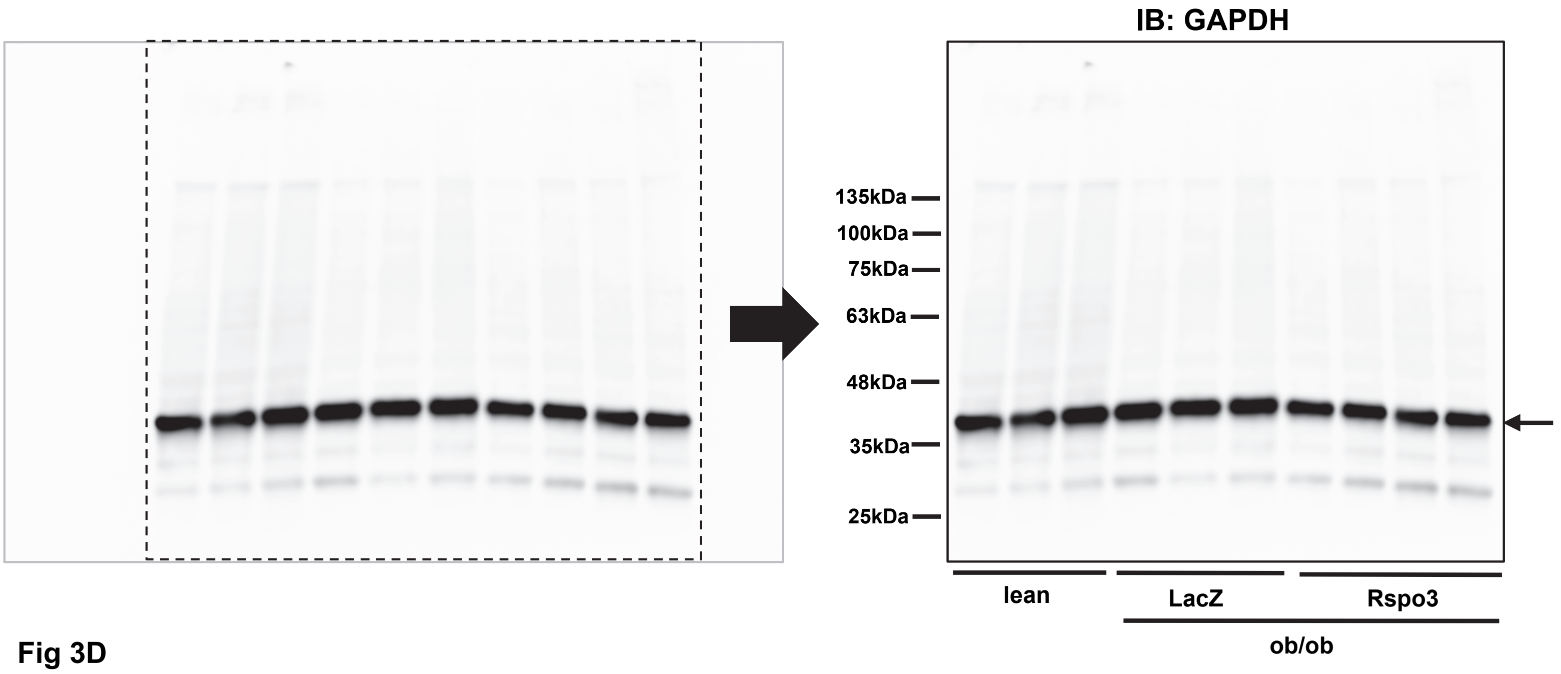

Fig 3D

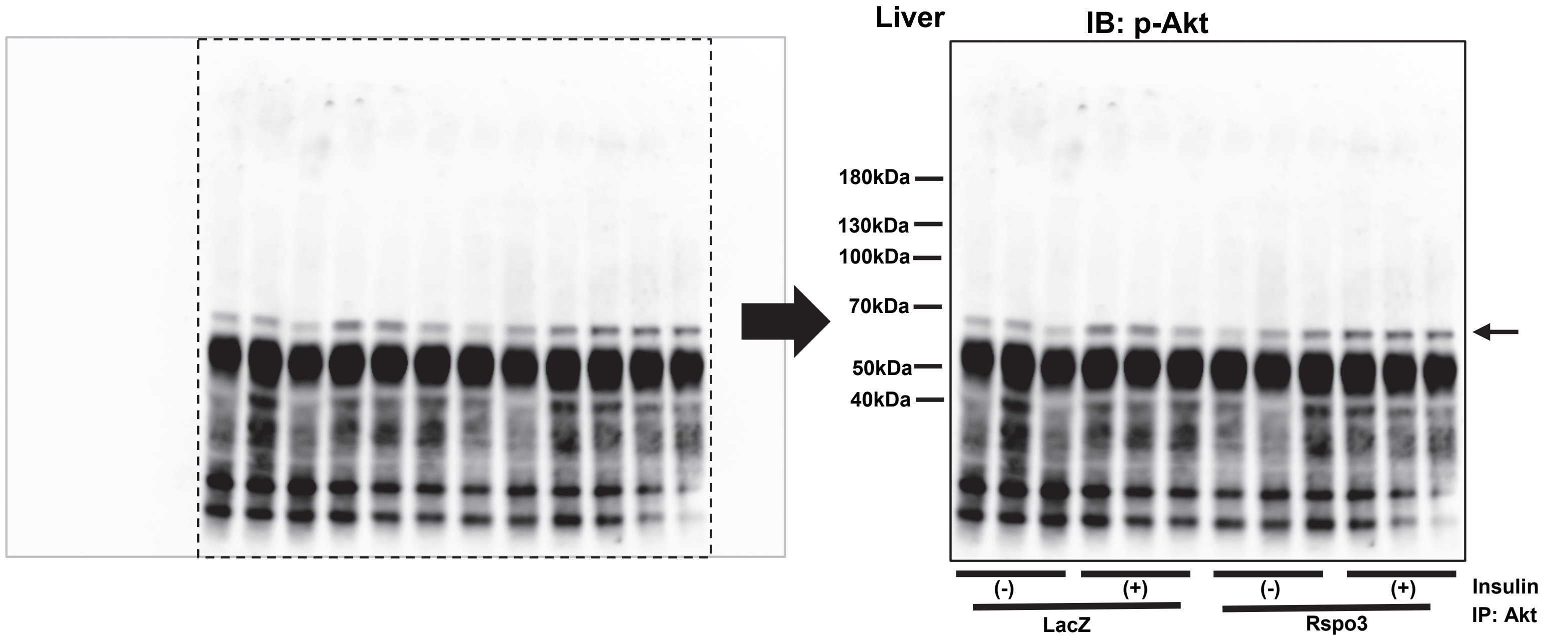

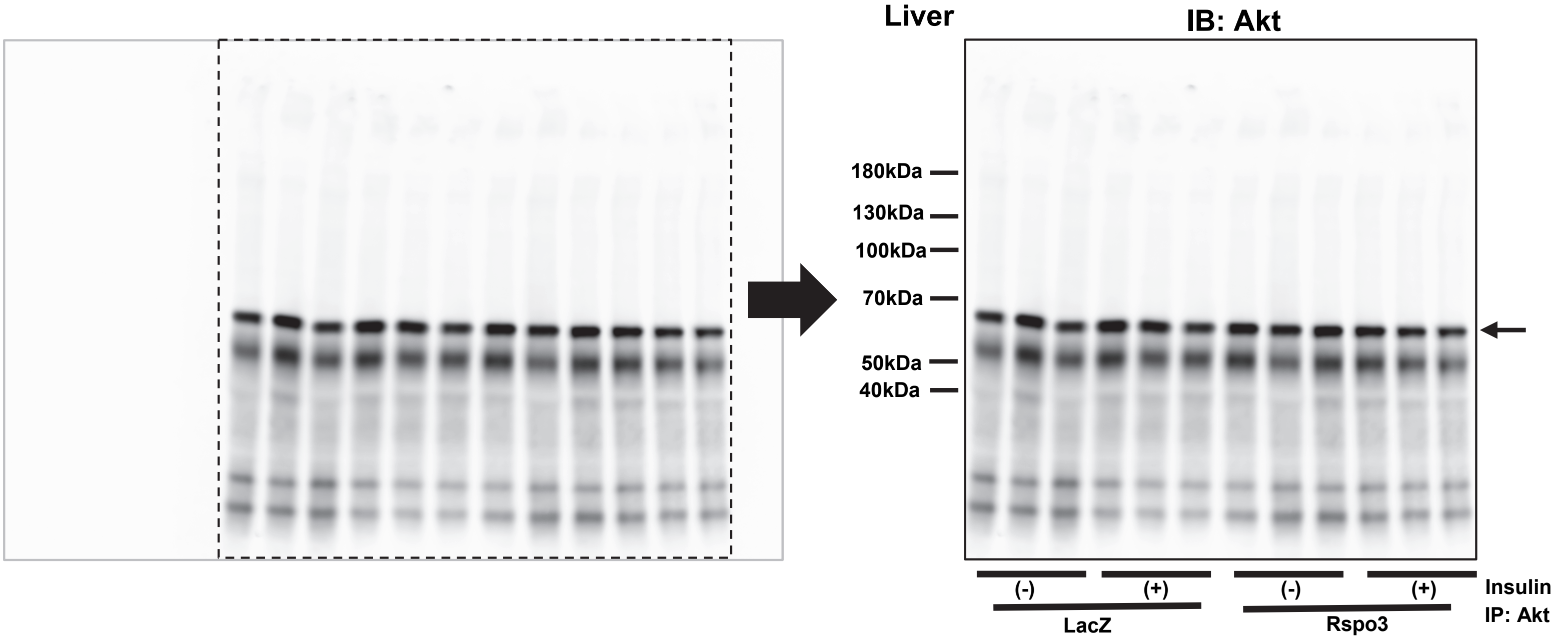

Supplemental Western Blot 4. Full western blots in Fig 3A and 3D

Fig 3E

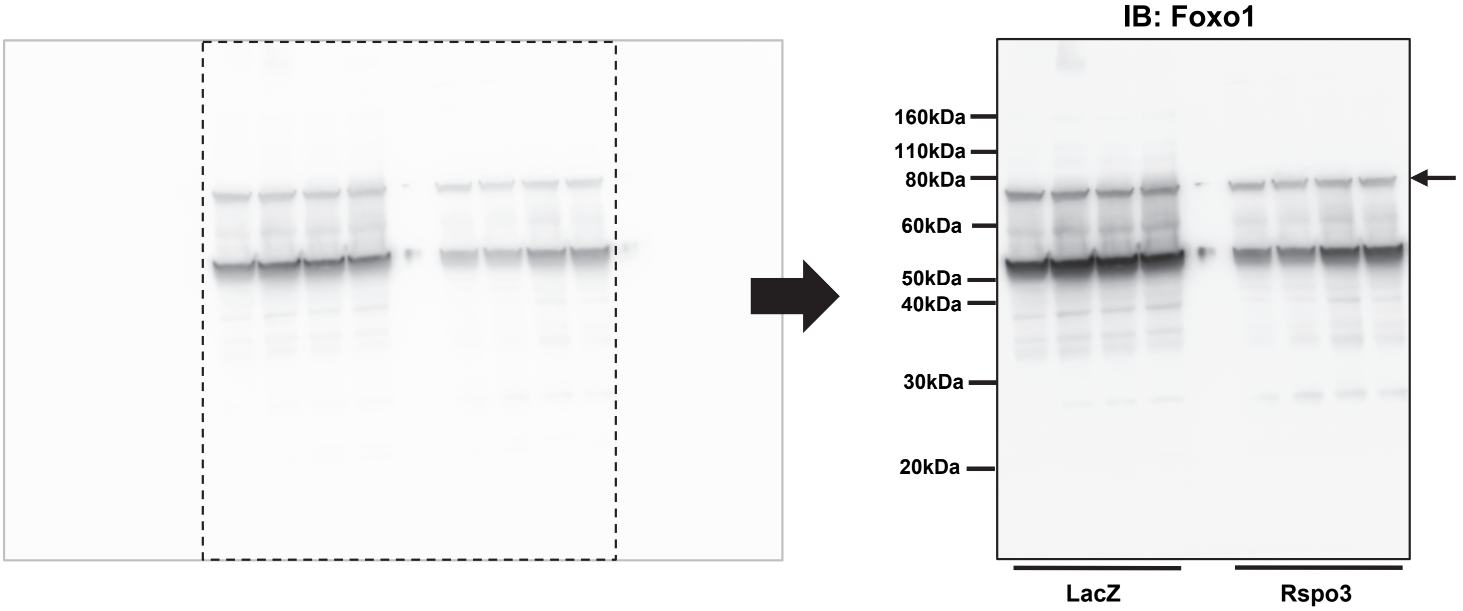

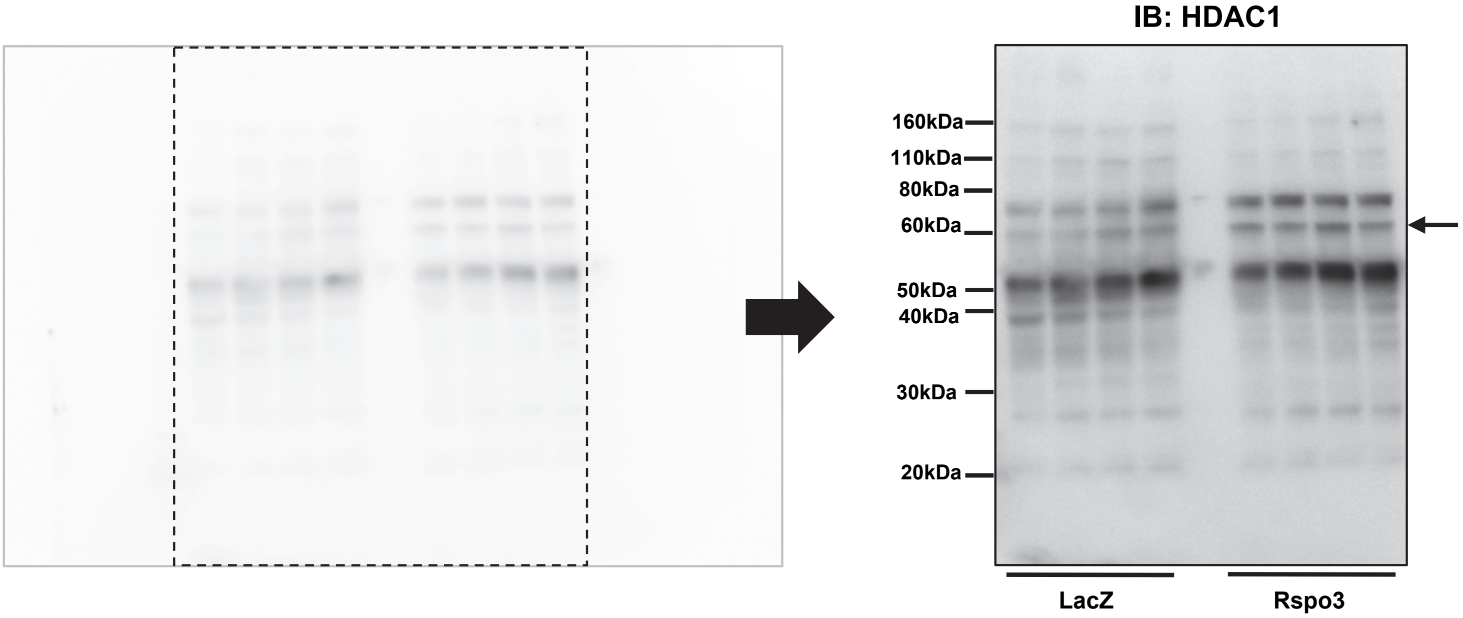

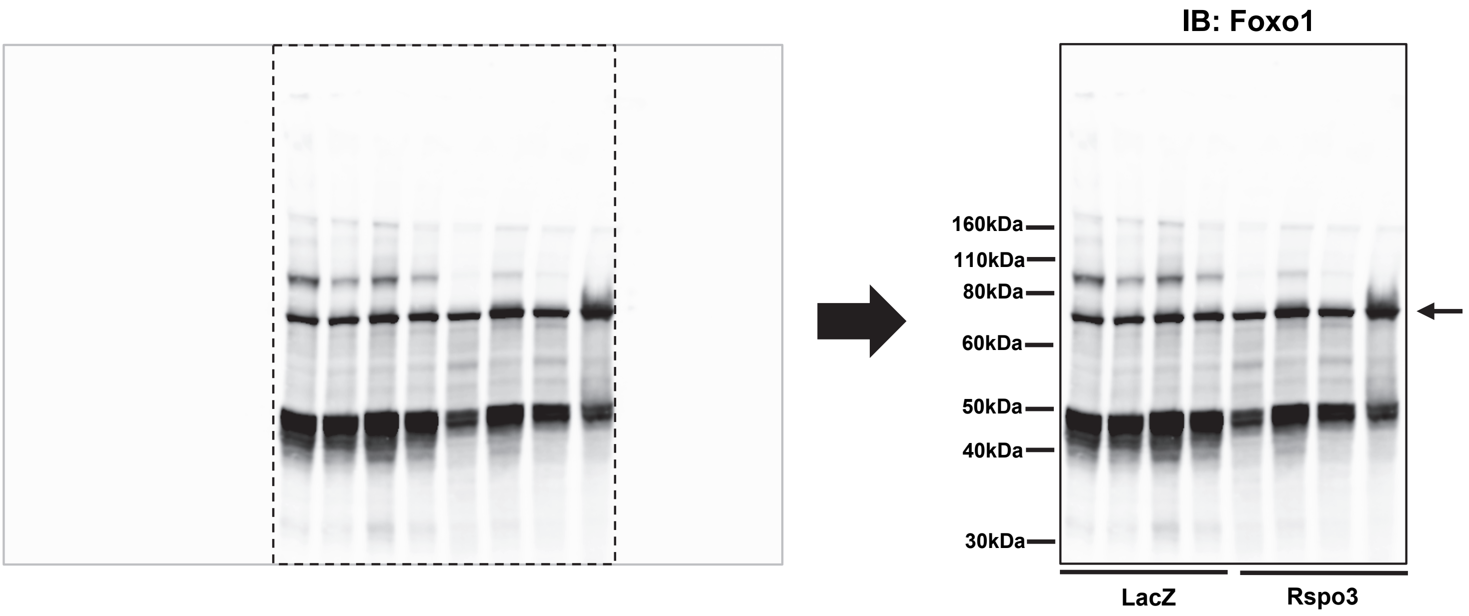

Supplemental Western Blot 5. Full western blots in Fig 3E

SWB 6. (Kenji Uno)

Fig 3E

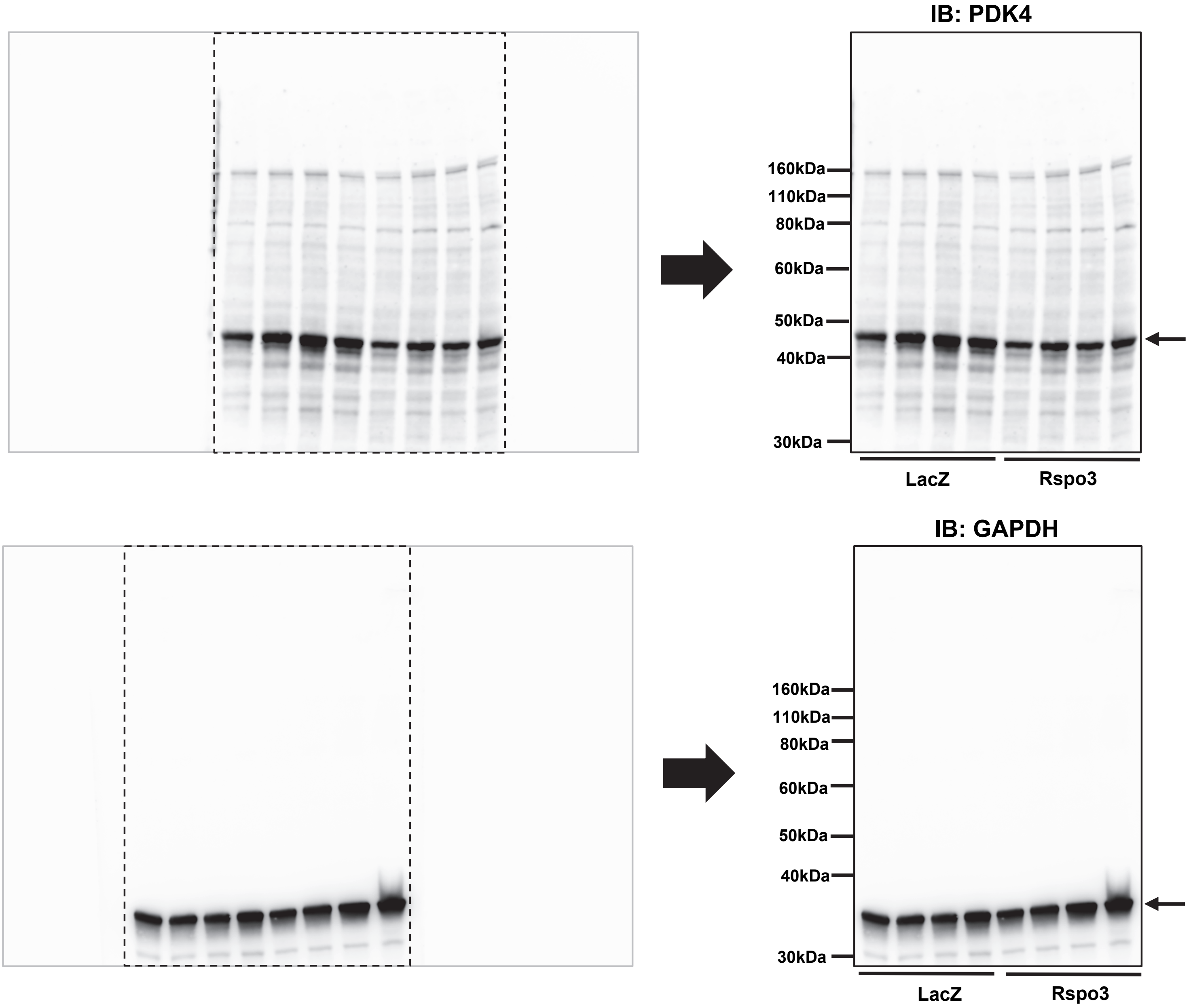

Fig 3K

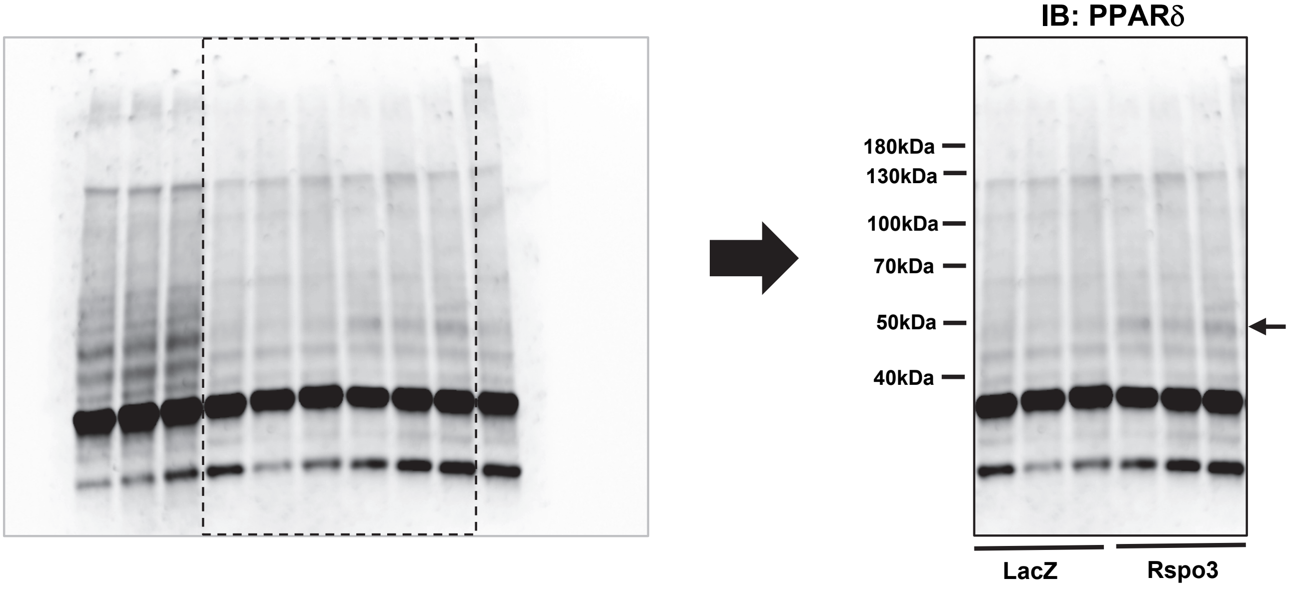

Supplemental Western Blot 6. Full western blots in Fig 3E and 3K

Fig 3K

SWB 7. (Kenji Uno)

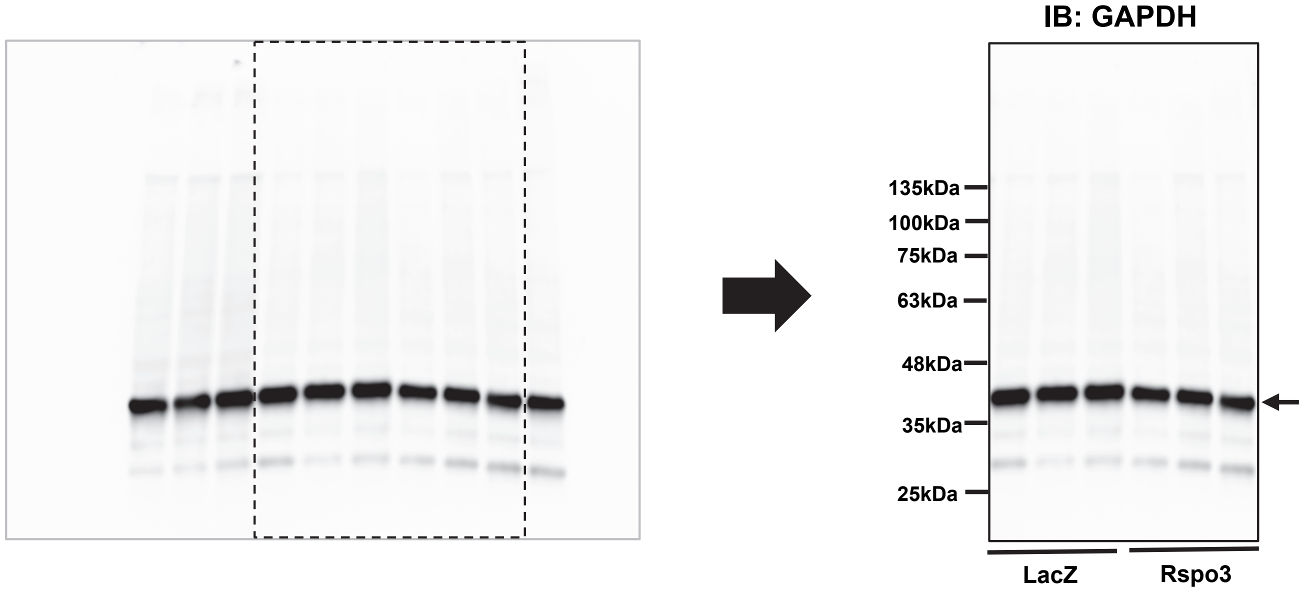

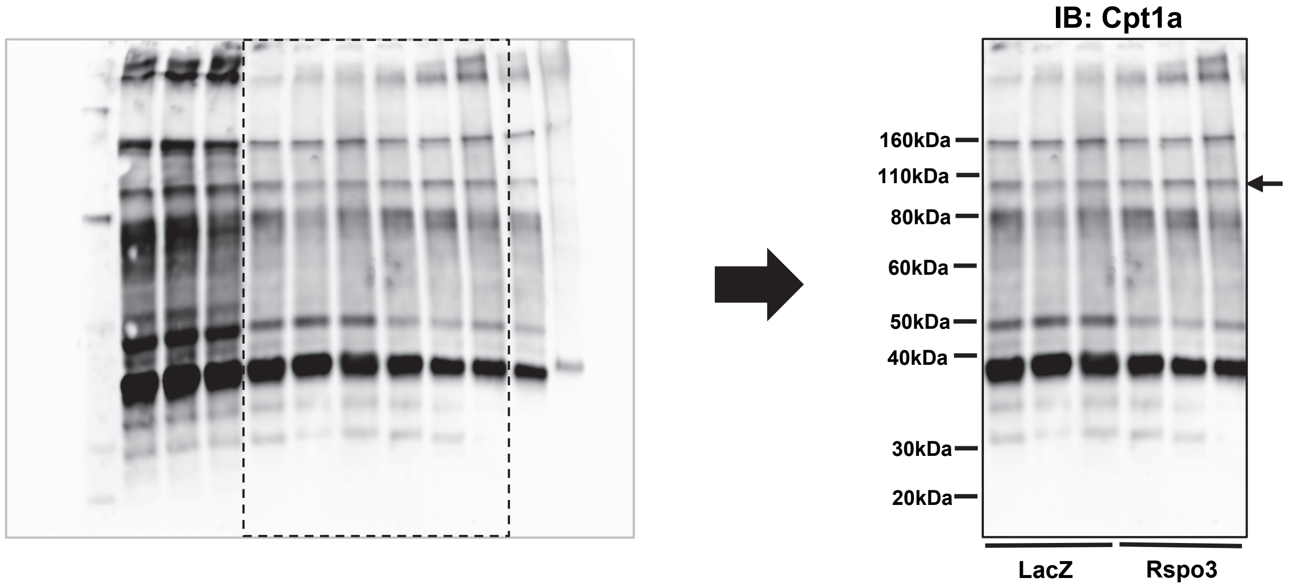

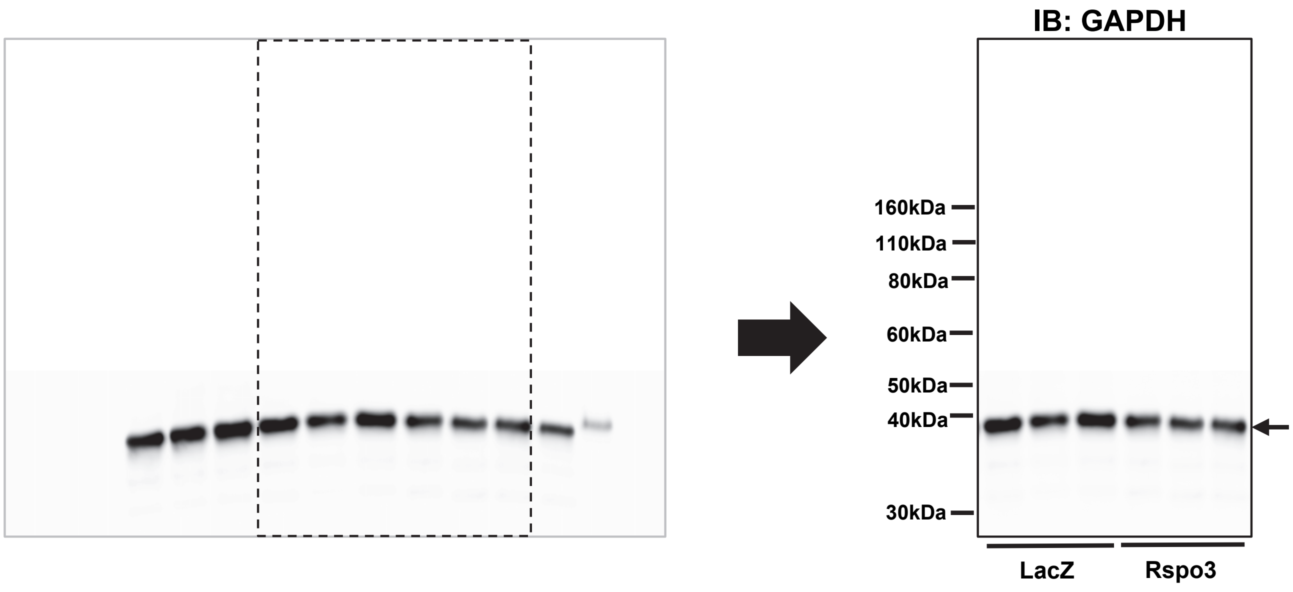

Supplemental Western Blot 7. Full western blots in Fig 3K

Fig 4C

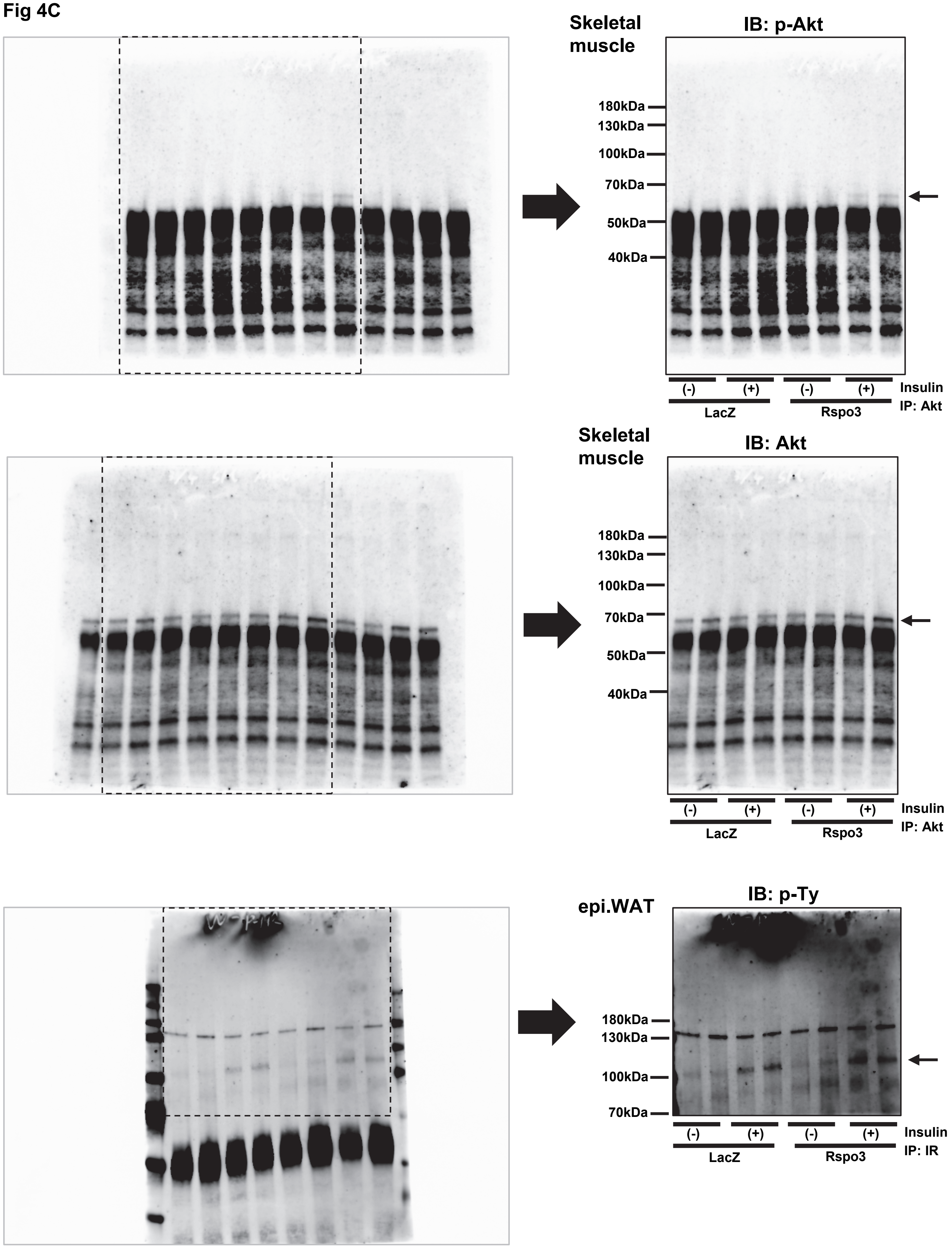

Supplemental Western Blot 8. Full western blots in Fig 4C

Fig 4C

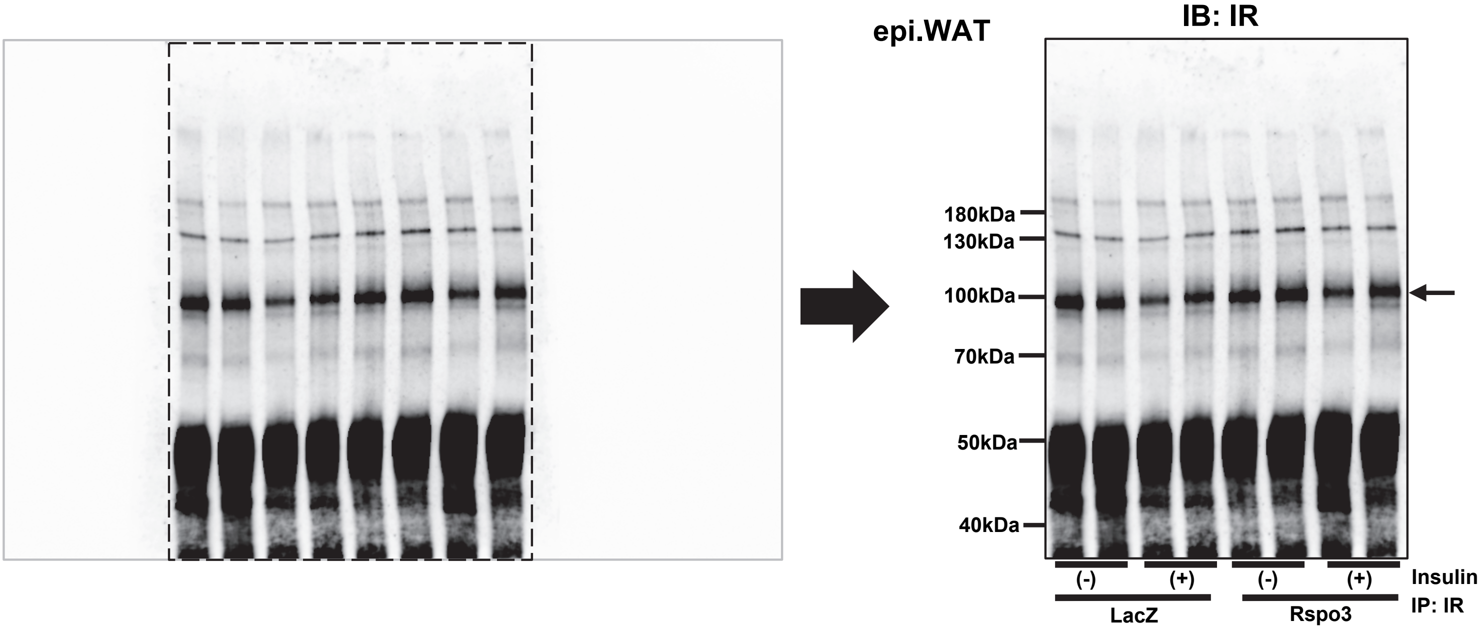

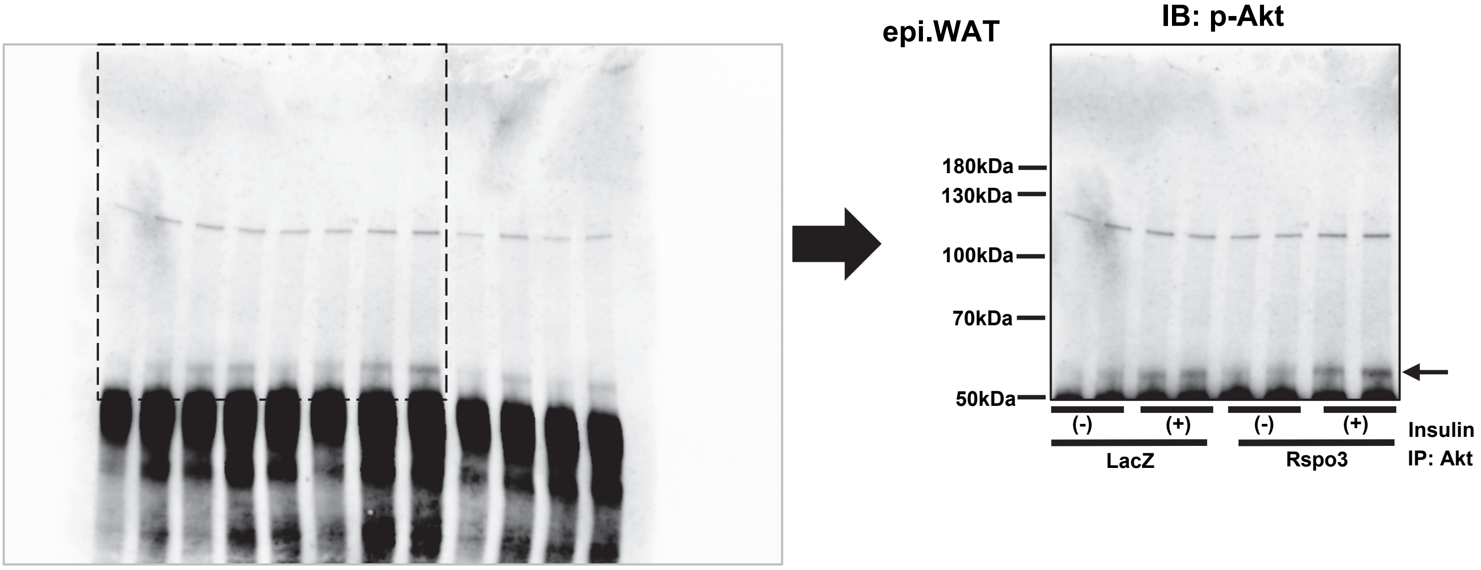

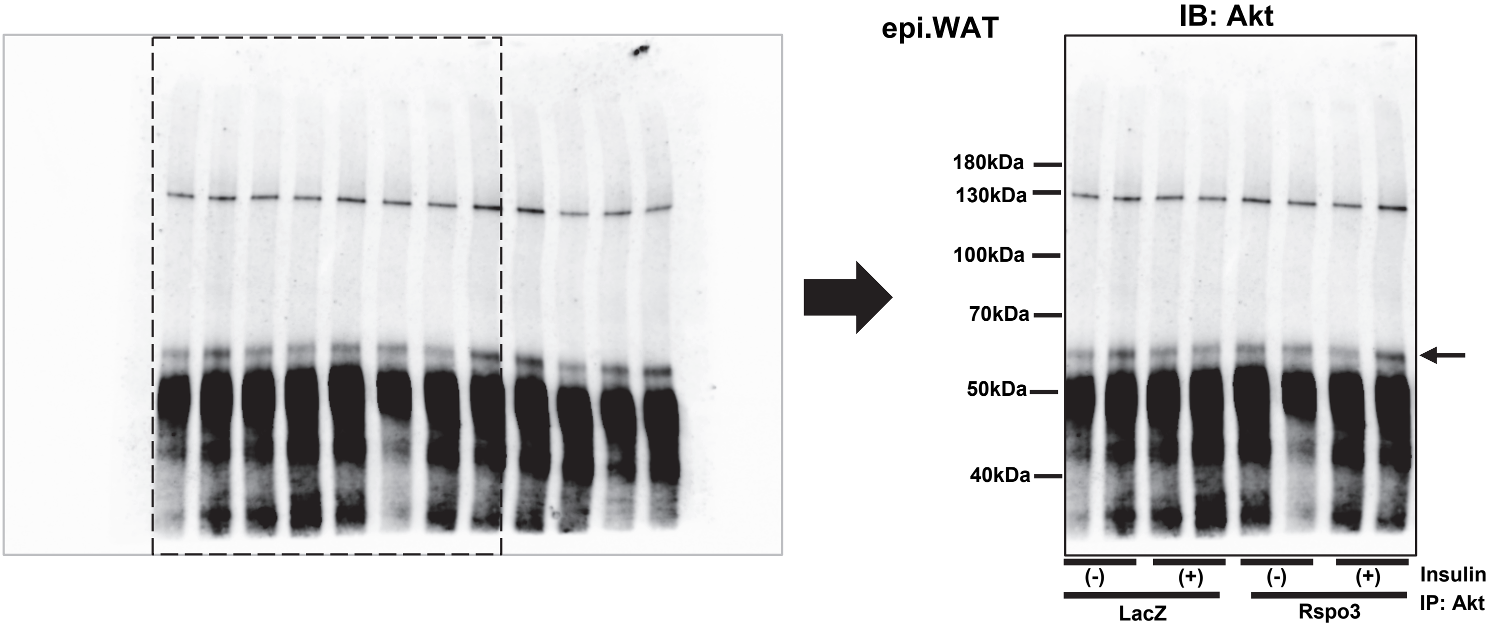

Supplemental Western Blot 9. Full western blots in Fig 4C

Fig 6A

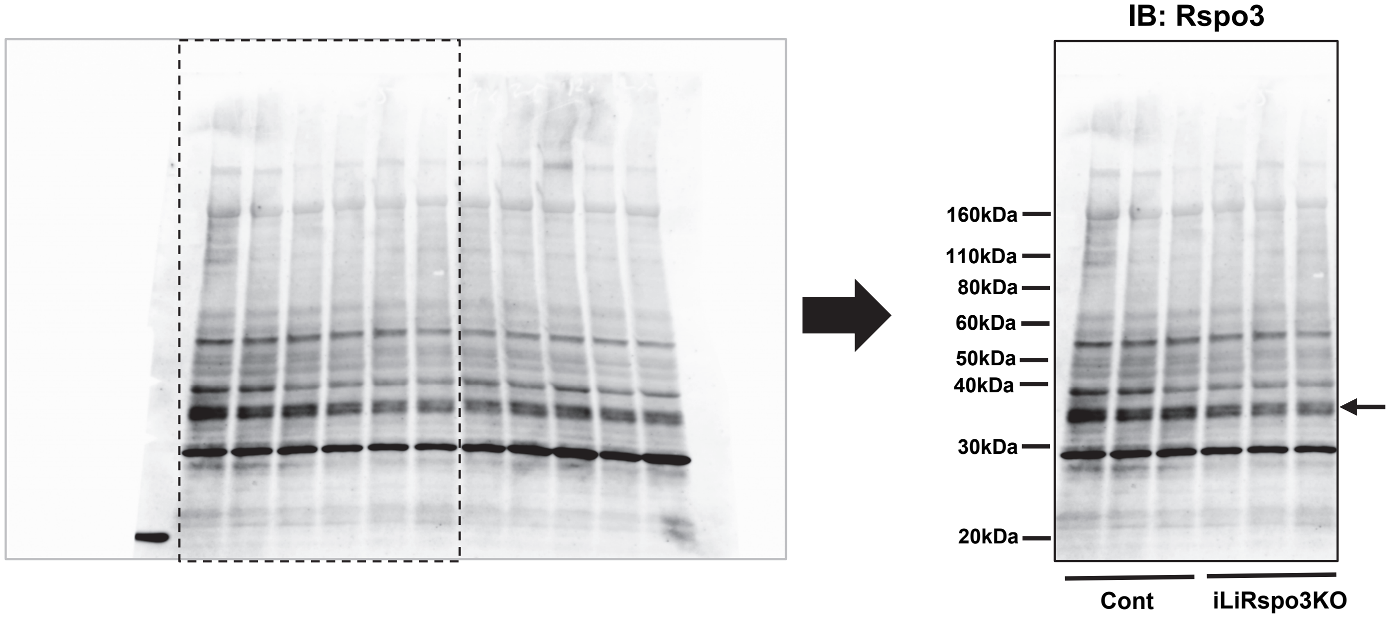

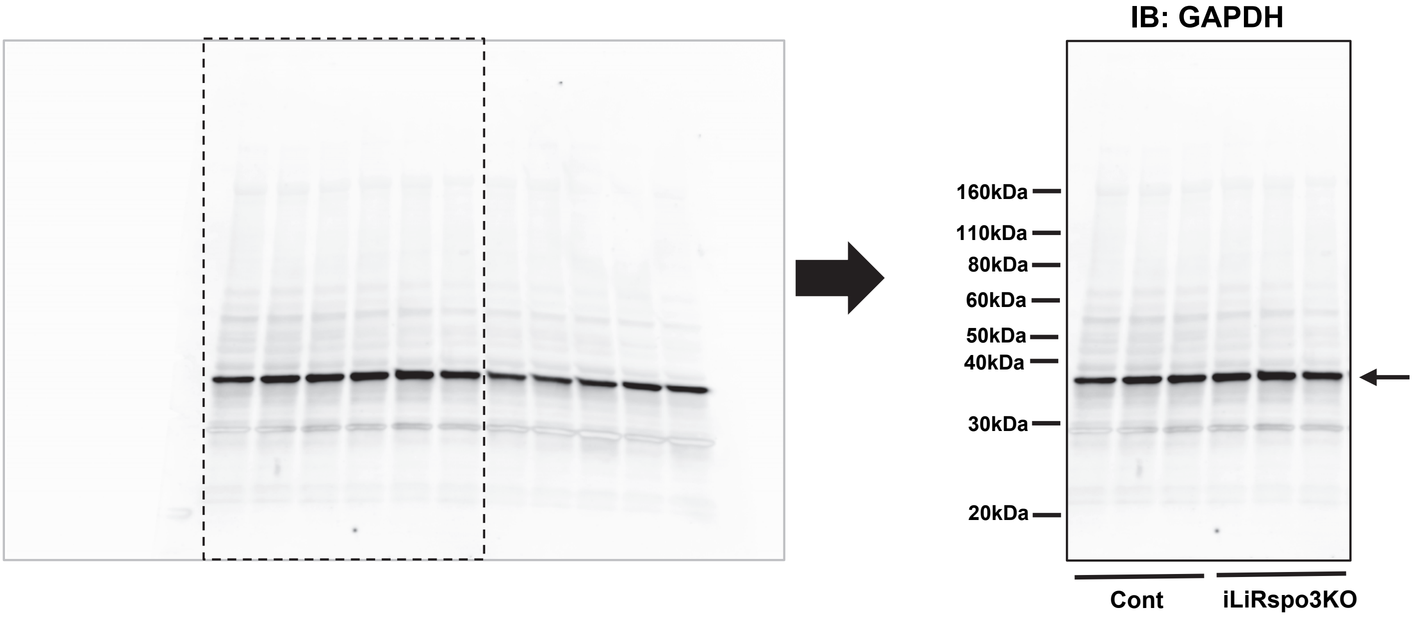

Supplemental Western Blot 10. Full western blots in Fig 6A

Fig 6E

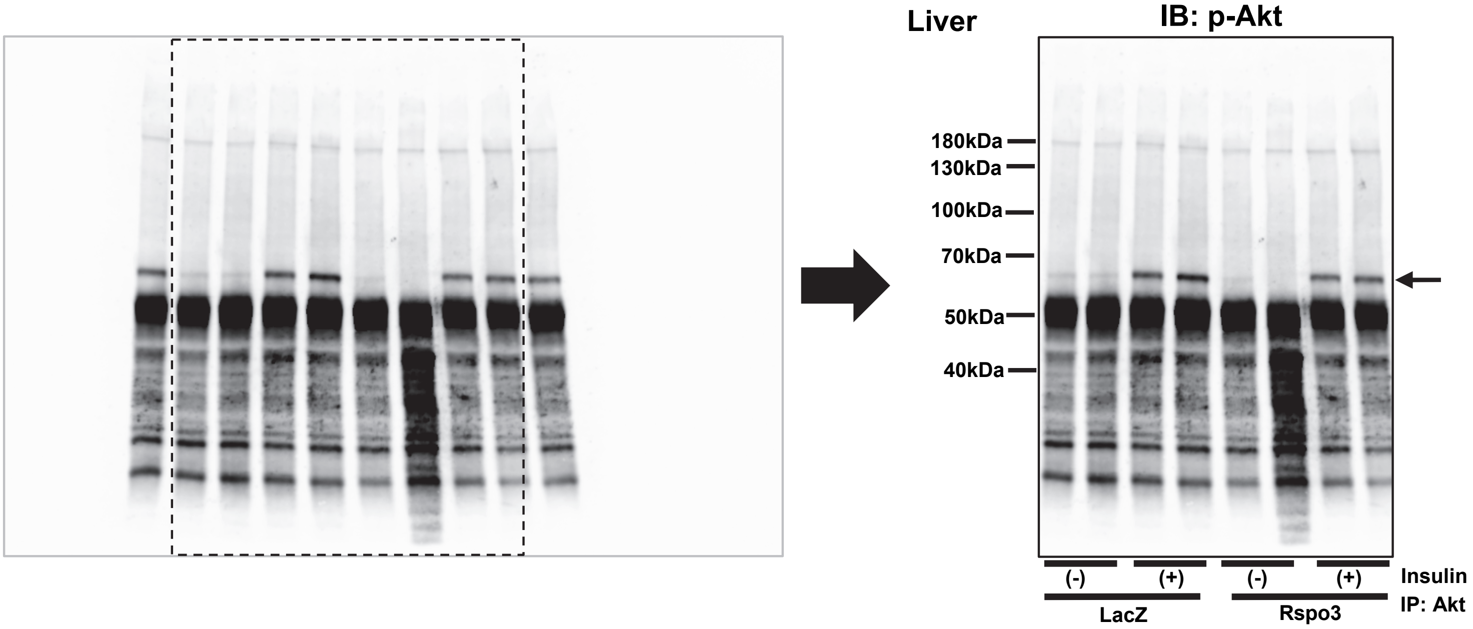

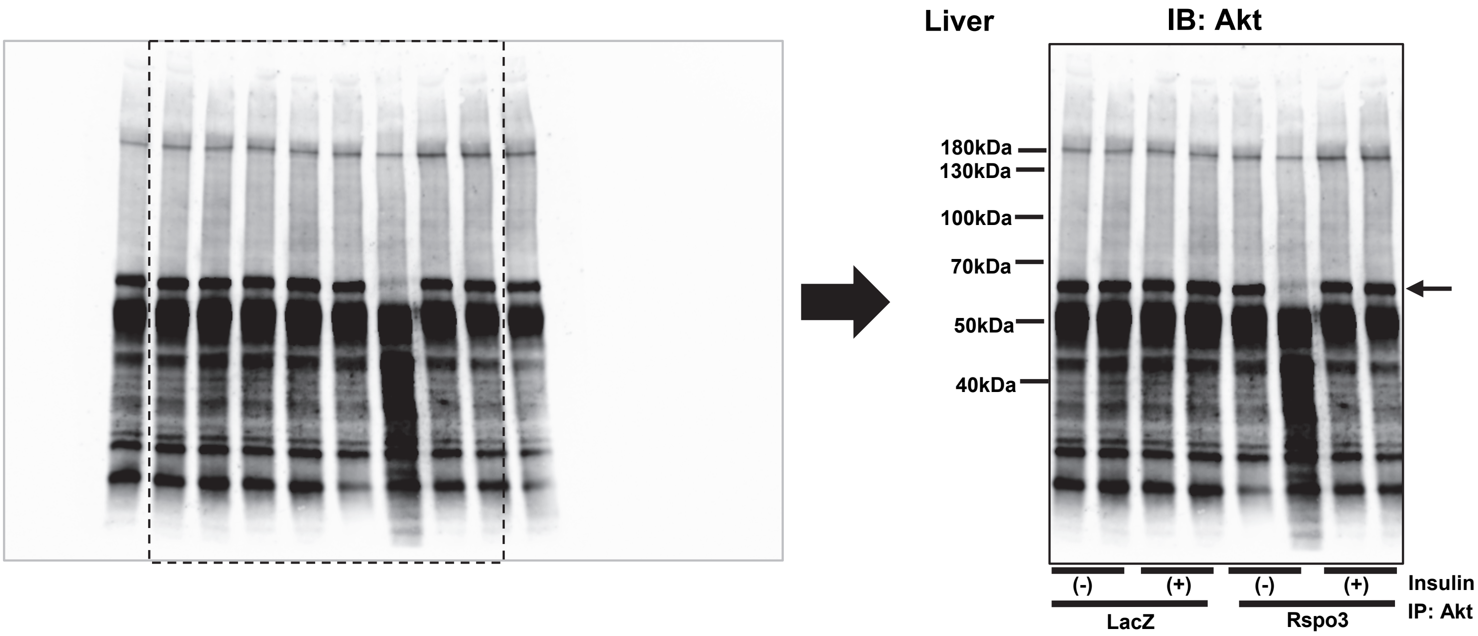

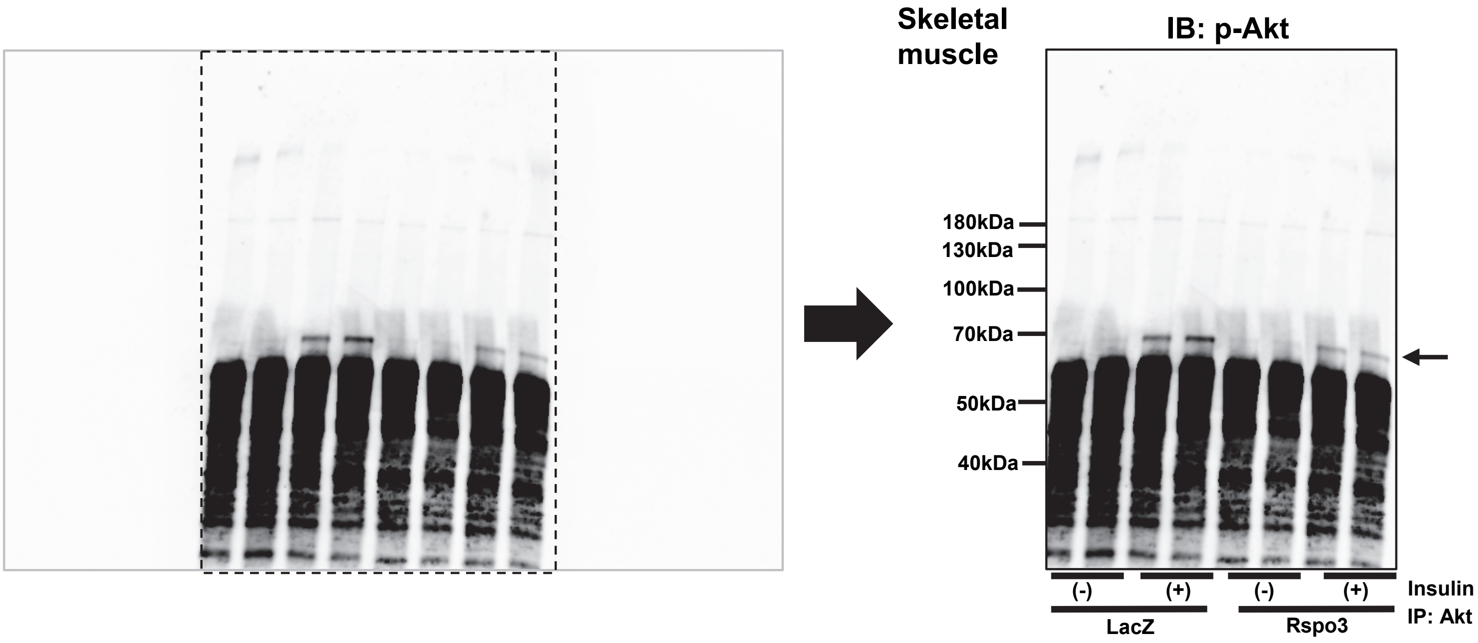

Supplemental Western Blot 11. Full western blots in Fig 6E

Fig 6E

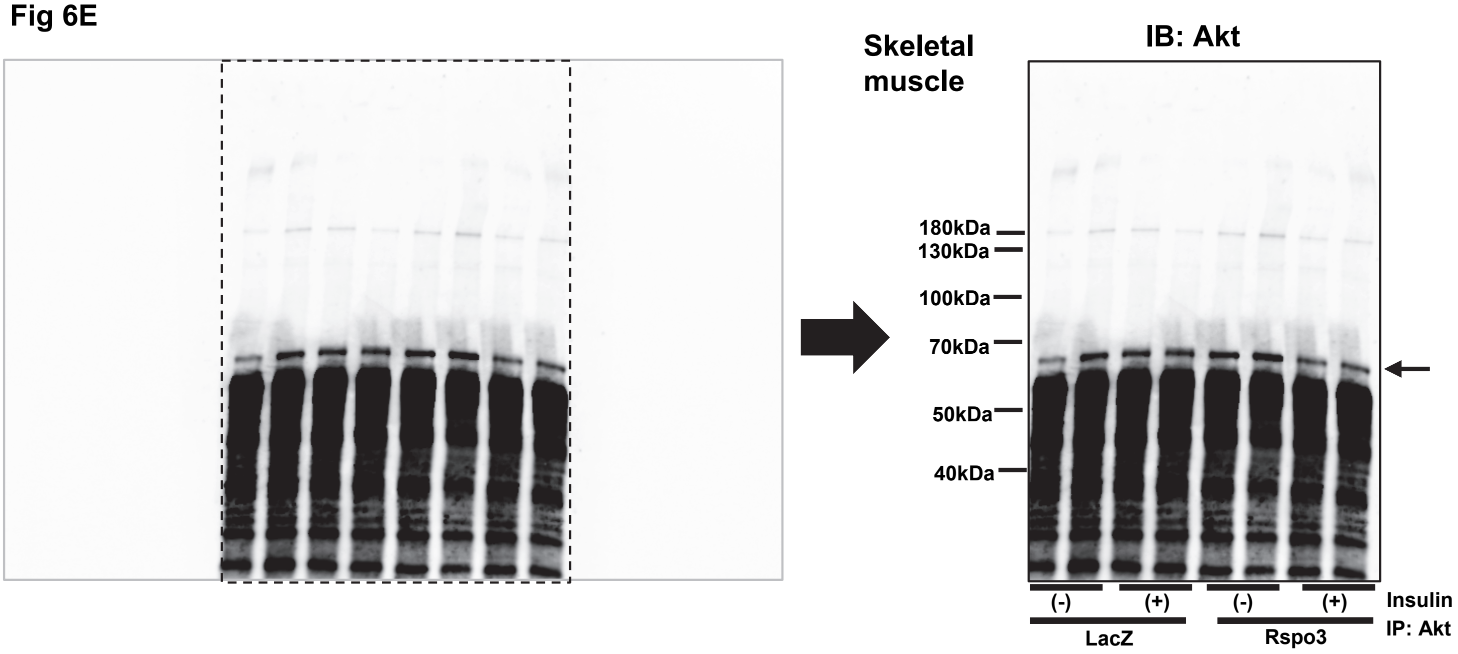

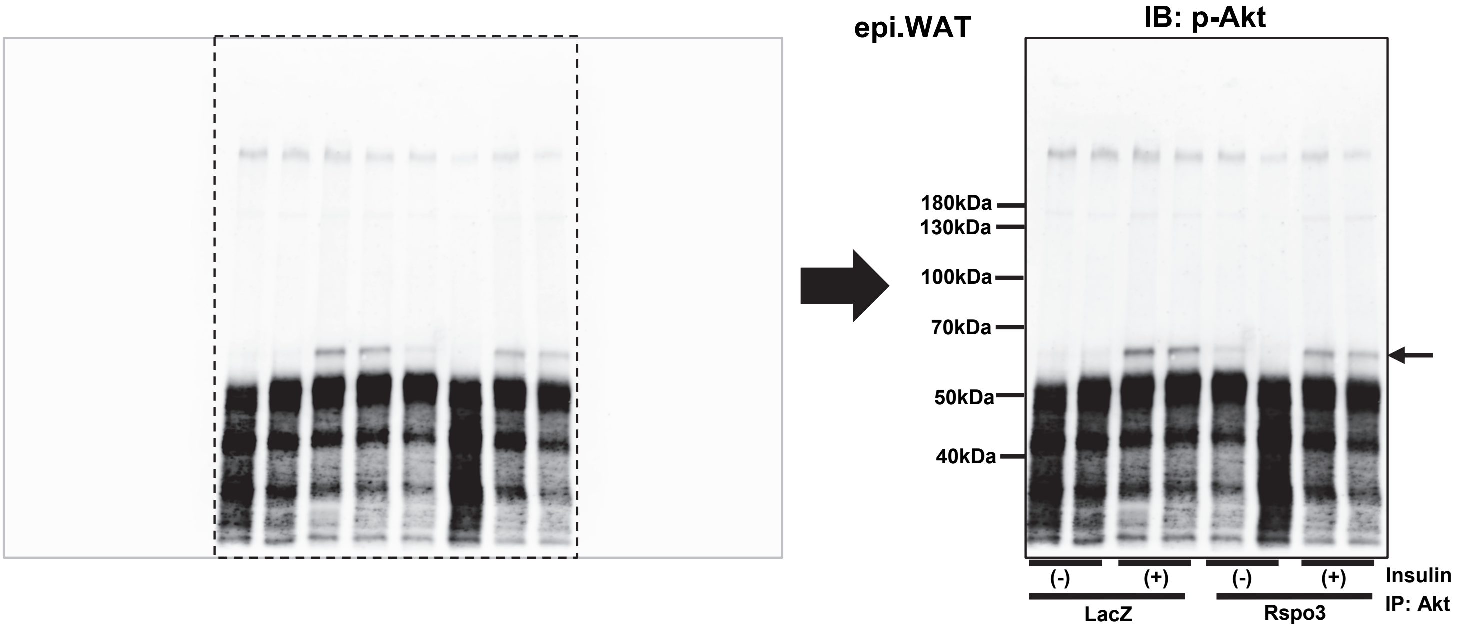

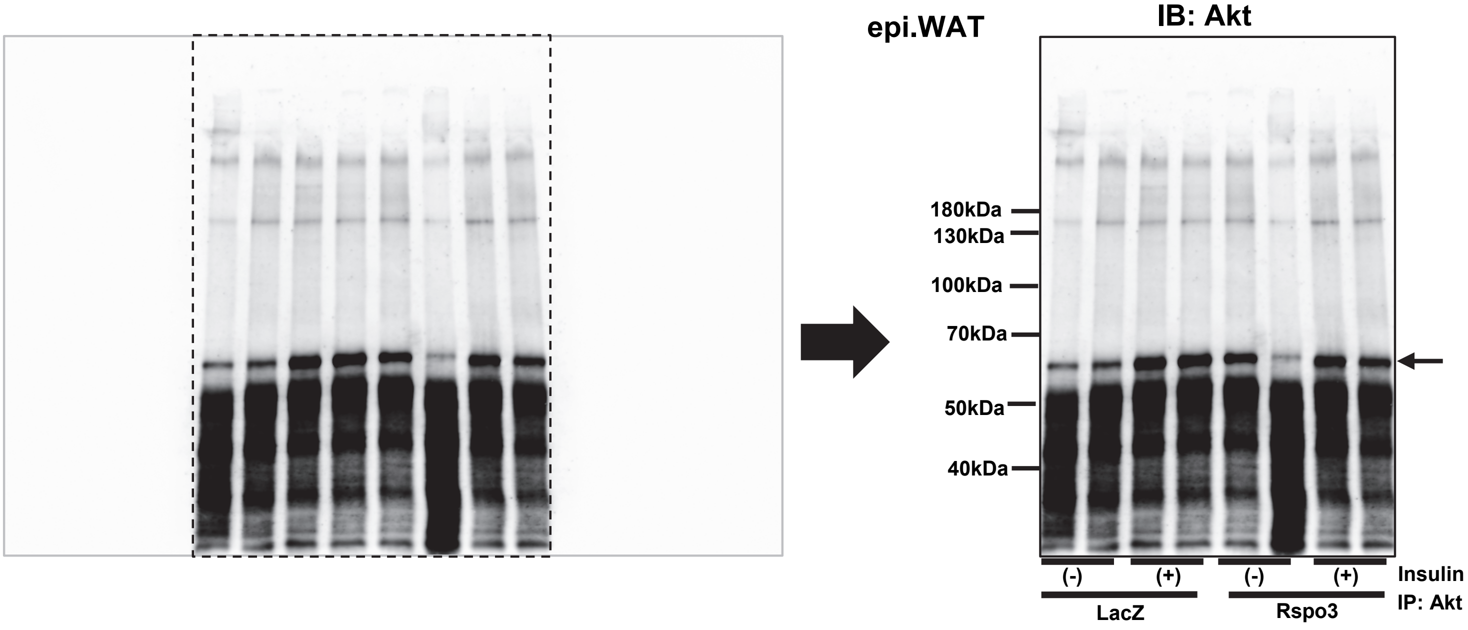

Supplemental Western Blot 12. Full western blots in Fig 6E

S12A Fig

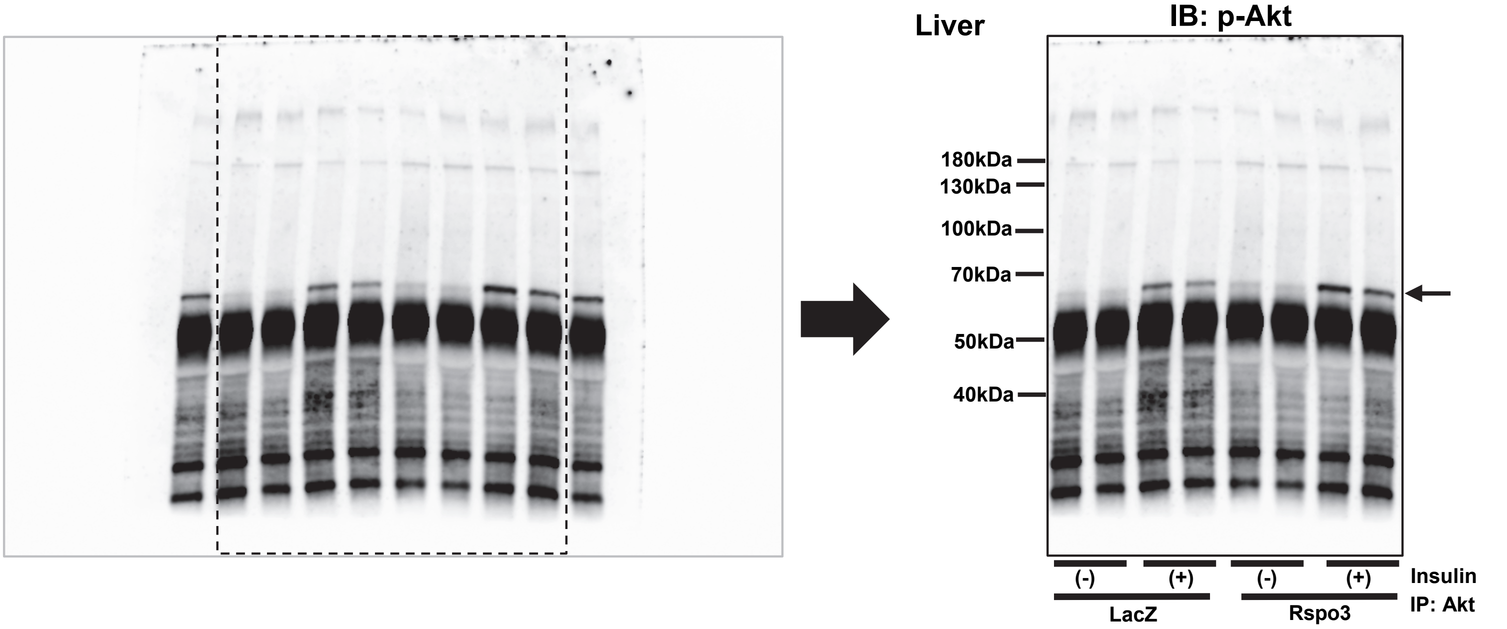

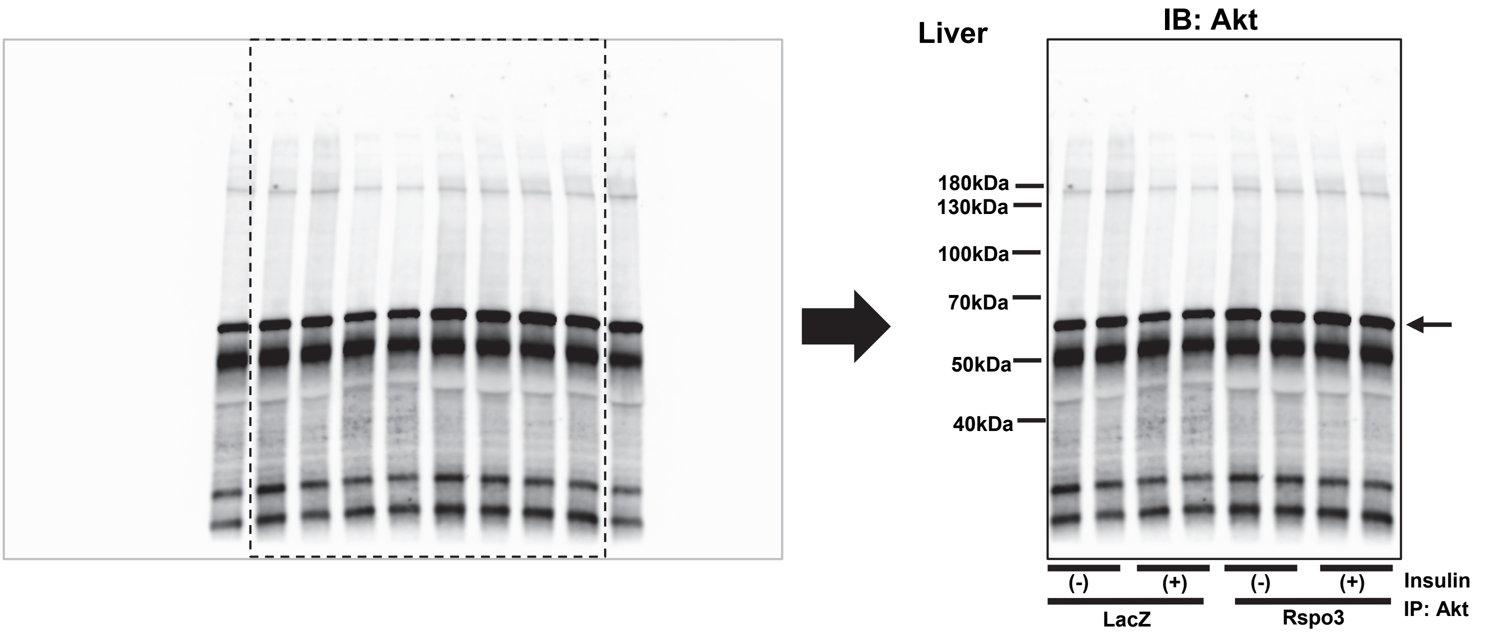

S13F Fig

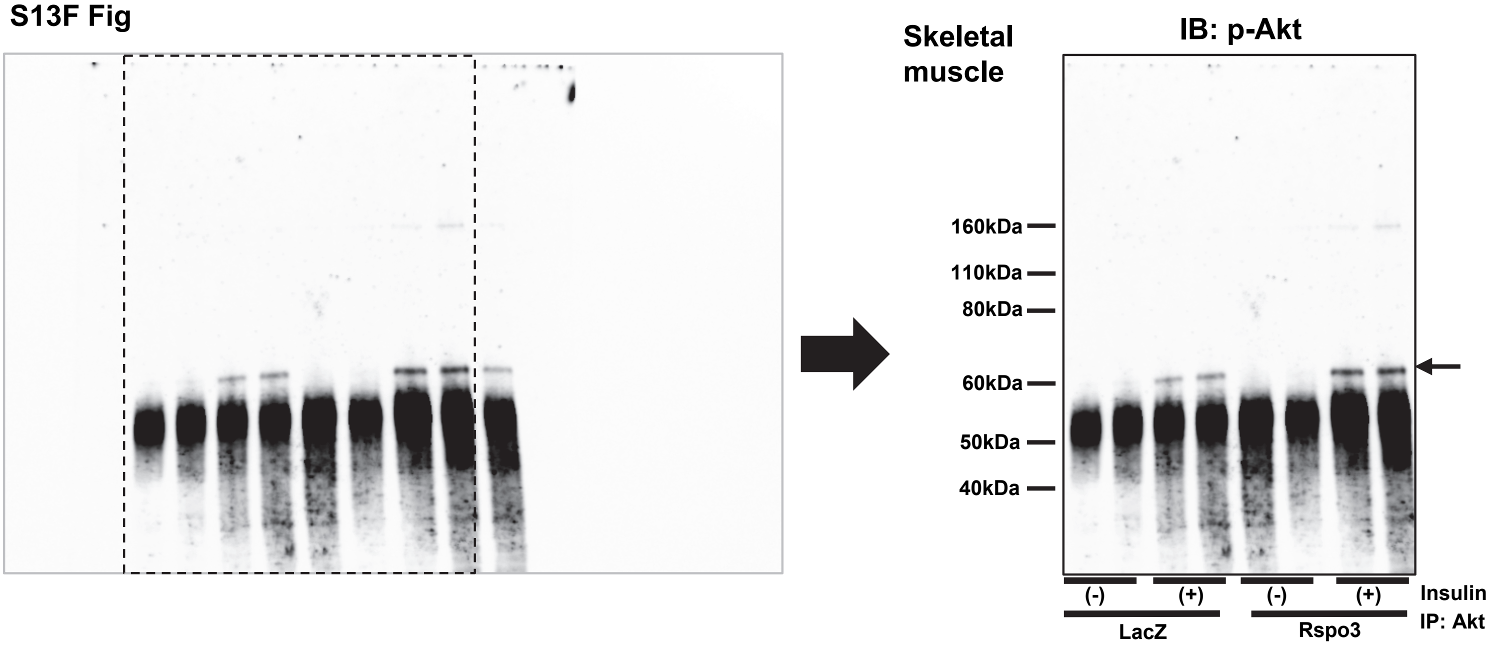

Supplemental Western Blot 13. Full western blots in S12A Fig and S13F Fig

SWB 14. (Kenji Uno)

S13F Fig

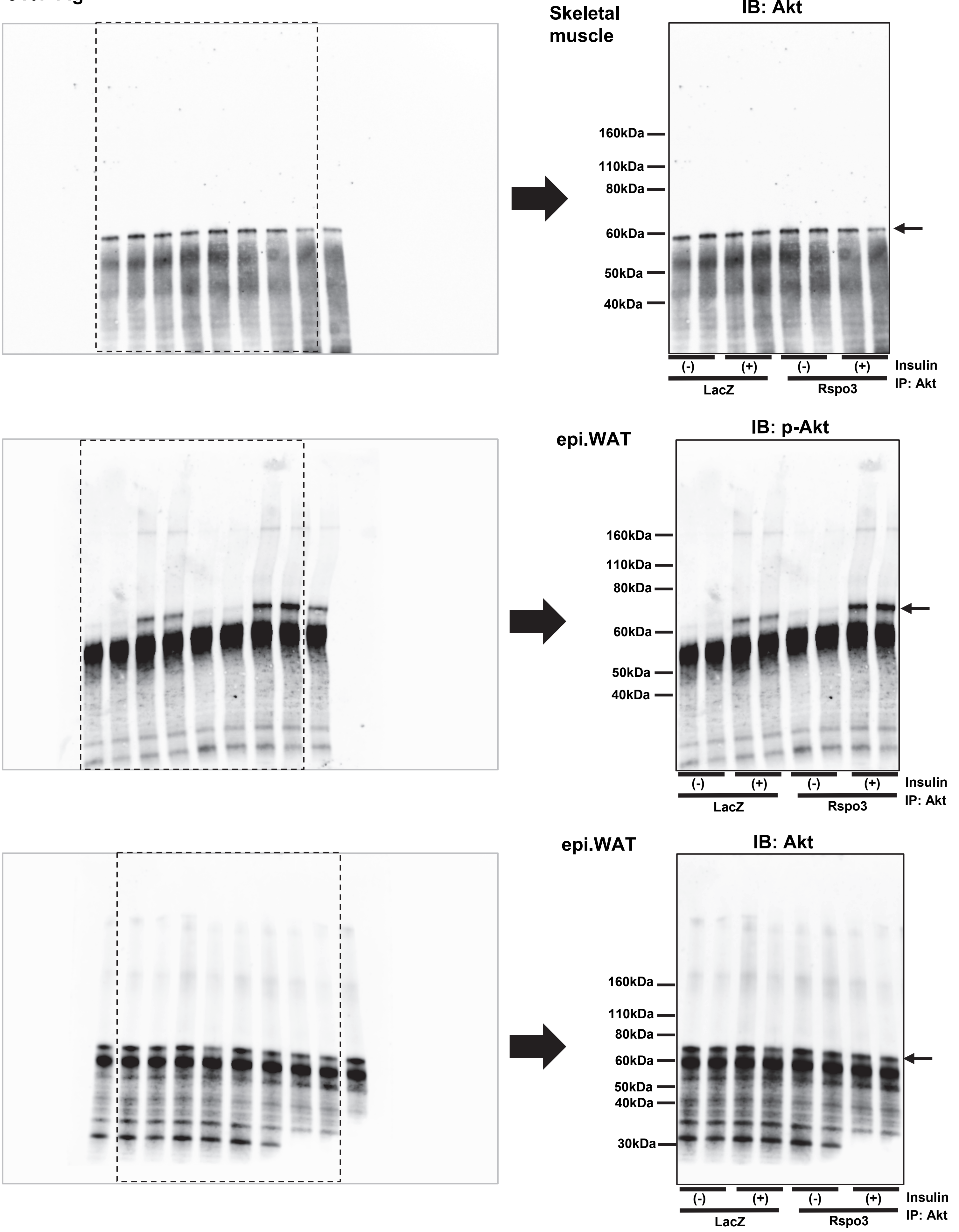

Supplemental Western Blot 14. Full western blots in S13F Fig
