# Supplementary material for: Rspo3-mediated metabolic liver zonation regulates systemic glucose metabolism and body mass in mice
Source: PLoS Biol. 2025 Jan 24;23(1):e3002955. doi: 10.1371/journal.pbio.3002955 (PMC11759367; doi:10.1371/journal.pbio.3002955)
Supplement: S1 Text — (DOCX) [file pbio.3002955.s024.docx]

**Supporting Text**

***qPCR Taqman probes***

Gapdh Assay ID: Mm99999915_g1

Rspo3 Assay ID: Mm01188251_m1

Pepck Assay ID: Mm01247058_m1

GK Assay ID: Mm00439129_m1

Srebp1c Assay ID: Mm00550338_m1

Fasn Assay ID: Mm00662319_m1

PPARγ Assay ID: Mm00440940_m1

PPARα Assay ID: Mm00440939_m1

PPARδ Assay ID: Mm00803184_m1

Cpt1a Assay ID: Mm01231183_m1

Ucp1 Assay ID: Mm01244861_m1

PGC1α Assay ID: Mm01208835_m1

β-catenin Assay ID: Mm00483039_m1

SCD1 Assay ID: Mm00772290_m1

Ki67 Assay ID : Mm01278617_m1

***qPCR primers***

Gapdh 5’-GACAAAATGGTGAAGGTCGGTGTG-3’

5’-CAATGAAGGGGTCGTTGATGGC-3’

Rspo3 5’-ATGCACTTGCGACTGATTTCT-3’

5’-GGCAGCCTTGACTGACATTAG-3’

MyoD 5’-TACAGTGGCGACTCAGATGC-3’

5’-TAGTAGGCGGTGTCGTAGCC-3’

Pepck 5’-CTGGCACCTCAGTGAAGACA-3’

5’-TCGATGCCTTCCCAGTAAAC-3’

GK 5’-CCAGAAGGCTCAGAAGTTGG-3’

5’-TCTGGTGTTTCGTCTTCACG-3’

Axin2 5’-GCAGGAGCCTCACCCTTC-3’

5’-TGCCAGTTTCTTTGGCTCTT-3’

LGR5 5’-CTTCACTCGGTGCAGTGCT-3’

5’-CAGCCAGCTACCAAATAGGTG-3’

Oat 5’-CAATTACCATCCTTTGCCTGTA-3’

5’-GTACTGCCTGCCTTCCACAT-3’

Cyp7a1 5’-CACCATTCCTGCAACCTTCT-3’

5’-TTGGCCAGCATCTGTAATG-3’

Rdh9 5’-GAGCGTGTTGGGAACAGAG-3’

5’-GTTTCTTCATCCACTCGTTGG-3’

Cyp2f2 5’-CCGGAACTTTGGAGGCATGAA-3’

5’-GGTCATCAGCAGGGTATCCAT-3’

Hsd17 5’-GATCCTGGAAAAAGGACCTGG-3’

5’-GTCTGAAGAGGGTGTCAAATCC-3’

BCAT2 5’-CTGATCGGGAATGAGCCCTC-3’

5’-TCTCCAGGGAAGTAGGAGCC-3’

BCKDH 5’-ATTGCCCAGGGATCAAGGTG-3’

5’-CACTGCTGCCCGGTAAAGTA-3’

β2AR 5’-TTCGAAAACCTATGGGAACG-3’

5’-GGGATCCTCACACAGCAGTT-3’

β3AR 5’-GGGAGGCAACCTGCTGGTA-3’

5’-GAAGTCACGAACACGTTGGTTATG-3’

***Rspo3 fragments for adenovirus***

GTAACTATAACGGTCATGCACTTGCGACTGATTTCTTGTTTTTTTATCATTTTGAACTTTATGGAATACATTGGCAGCCAAAACGCCTCCCGAGGAAGGCGCCAGCGAAGAATGCATCCTAATGTCAGTCAAGGCTGCCAAGGAGGCTGTGCAACGTGTTCAGATTACAATGGCTGTTTGTCATGTAAGCCCAGACTGTTTTTTGTTCTGGAAAGGATTGGCATGAAGCAGATAGGAGTGTGTCTCTCTTCGTGTCCAAGTGGATATTACGGAACTCGATATCCAGATATAAATAAATGTACAAAATGCAAAGTTGACTGTGATACCTGTTTCAACAAAAATTTCTGCACAAAGTGTAAAAGTGGATTTTACTTACACCTTGGAAAGTGCCTTGACAGTTGCCCAGAAGGGTTAGAAGCCAACAATCATACTATGGAATGTGTCAGTATTGTACACTGTGAGGCCAGTGAATGGAGTCCATGGAGTCCATGTATGAAGAAAGGAAAAACATGTGGCTTCAAAAGGGGGACTGAAACACGGGTCCGAGATATACTACAGCATCCTTCAGCCAAGGGTAACCTGTGCCCCCCAACCAGCGAGACAAGAACTTGTATAGTACAAAGAAAGAAGTGTTCAAAGGGAGAGCGAGGAAAAAAGGGAAGAGAGAGAAAACGAAAAAAACTGAATAAAGAAGAAAGAAAGGAAACAAGCTCCTCCTCTGACAGCAAAGGTTTGGAGTCCAGCATTGAGACCCCAGACCAGCAGGAAAACAAAGAGAGGCAGCAGCAGCAGAAGAGAAGAGCCCGAGACAAGCAACAGAAATCGGTATCAGTCAGCACTGTACACTAGGGAGAAAGAGGTAAT

coding region of Rspo3 (834 bases)

The underlined sequences indicate the recognition sequences of In-Fusion ligation in pAdenoX vector.

***CRISPR RNA (crRNA)***

5’ crRNA GGGACAATTTGAACCCGGCA

3’ crRNA CCACAGGAGATAGCAACTCG

***trans-activating CRISPR RNA (tracrRNA)***

aaacagcauagcaaguuaaaauaaggcuaguccguuaucaacuugaaaaaguggcaccgagucggugcu

***single strand oligo donor DNA (ssODN)***

GAAATTAGGAACAAGAGAGTCGAGTGGTGAACTTCCCAAGAGCAGTTGCTGGAGTGAGGCAGGGTCTCTGAAGAACTGAAGGACATGGCCACTACTATGTGGTTAGATCTGCCAGAGGCCAAGGACAACAGAAGCATTGGGTCTGGGGTATTTGGTACAGCTGGGACAGAAACCACCAAACCCCAGTGCGCCCACCTTGCATAACTTCGTATAGCATACATTATACGAAGTTATCGGGTTCAAATTGTCCCTCCCCCAACCCTGCTCCCGTTCTCCCGCCGTCTTTGCTGAGTCCAGAGATGCTCCGCCACAGACCCGGGACCCCTTAGGCGCCTCTTCCCGTCCATCTGCCAAATCGCAACGCCTGTACTTTTCCAGCTCCCTCTTTCCCTCTTGAAATTGAAAGTTATTGAGGTCGCTCCGGGTTGTTGCTCCTCCCCGTCCCCGAGCTCCCCCCGCCCCCCGCAGCCATCCCGTCTCCCCCTCCCTCCTCCTCCTTCCCTATGGGGTGCTCCTAGCCCGGCCCAACACTGGAGCGGCTCCTGCTCAGAACGCCAGAAGCAGCTCGGGTCTCTCCAGCGCCCCTTGACCATGGCTGCGGTACCCACGGCGTCCGCTTCCCTGCGCTCCCGGGGTCCCTGCCACAGCCGCAGCCGCTGCAGCCTCTGAGCCCCAGGGGCCACTGCTCGCCTGGATTCCGCCCGCAGCCGCCGCTGCTGTGCAACCGAGGCTAACCTGCGGCCAGCCAGGAGGCTCCTGCAACCTTCGCTCGCGGCGATGACAGCCACCCCAGAGCAGCCGGCTGTGTTCGGACAATTTGAGAATGCAATTGTTGGTTTCCCGGTCCACCCGTCCCGCTTCGCTTGCCATCACAGCACGCCTGTTGGATCTCAGTGGAGAAGTCCCGCTGCTCTGGTTTTTCTACTCTTCGTATAGACTCGCCTAACACCTACATACATATTTTTCTTTAAAAAAAAACATTAAATATAACTAACAGTGAAAAGAAAAAGGAGAGAAAAAAGGGAAACATTACAGGGTTACTATGCACTTGCGACTGATTTCTTGTTTTTTTATCATTTTGAACTTTATGGAATACATTGGCAGCCAAAACGCCTCCCGAGGAAGGCGCCAGCGAAGAAGTAAGTTTGGGGTTTTCTTTTTTTTTGGGGGGGGGAGTGGGAGAGGAGGGGGTATTGTTGTTGTTTGTTTTTGTTGTTGTTGTTTTGGTTTTGCTTTGCGTTATGTCTTTGCTCCCTTCCACCACATTCACCTGTCAGCTGGTTTTGCCTGCCCAGACCCAGCTGCTGGAGGAAACAGTCTTTTCAACCTACACCCTATGCCTCCCACAGGAGATAGCAACATAACTTCGTATAGCATACATTATACGAAGTTATTCGGGGCACTGGCTCTTCTACTCCAGTACACAAACCAGTACTGGAGTCGGTGCACCCGGTGCTAGTCTGCCCCCCCCCCCCCCCCCCCCCCCCTGTAAATCAGGATCTGGAAATCTTTCTTTCTTTCCTAGATTCGCTGTAGGGATTTGTGCTTTCCTTTGAGGCTCCGACGGAGGCCTAGAGCCCCAGGCAGTTGGGTC

LoxP site (34 bases)

Exon1 (636 bases)
