# Supplementary material for: Rspo3-mediated metabolic liver zonation regulates systemic glucose metabolism and body mass in mice
Source: PLoS Biol. 2025 Jan 24;23(1):e3002955. doi: 10.1371/journal.pbio.3002955 (PMC11759367; doi:10.1371/journal.pbio.3002955)
Supplement: Attachment — Submitted filename: Responses.docx [file pbio.3002955.s025.docx]

Responses to the Reviewers’ comments

Reviewer #1:

In their manuscript entitled "Rspo3, a mediator of hepatic metabolic zonation, ameliorates diabetes and obesity predisposition via inter-organ communication" Uno and colleagues highlight neuronal and non-neuronal inter-organ linkages to be involved in hepatic Rspo3-mediated maintenance of systemic glucose metabolism and body composition. Therefore, the authors artificially increase Rspo3 expression using an adenovirus-based delivery system in lean and obese mouse models. They show that increased Rspo3 expression ameliorates obesity-associated features, reduces adipose tissue enlargement and restores insulin sensitivity and skeletal muscle quality. By performing a hepatic vagotomy the authors provide some evidence that the effects of Rspo3 include a partial neuronal involvement (ie ameliorated glucose intolerance). Moreover, they identify the hepatic PPARδ-to-muscle PPARα-axis to be involved in the regulation of fatty liver development and reduced skeletal muscle quality in obese mice. In line, the authors find these phenotypes to be exacerbated upon reduced hepatic Rspo3 expression.

Overall, Uno et al. highlight an interesting concept linking hepatic WNT signaling which controls liver zonation to the body`s overall health status via inter-organ communication. These findings have the potential to help identifying new therapeutic targets for treating obesity phenotypes. However, several shortcomings of the manuscript should be addressed before publication.

Major comments:

1. The authors claim that Rspo3 expression induced by adenovirus delivery is selective to the liver. However, they never proof this selectivity, and many adenoviral systems are not exclusively targeting the liver. It is a must, to assess Rspo3 expression in other organs (especially adipose tissue and skeletal muscle) to make sure that all observed effects are truly mediated via the liver as claimed by the authors.

As the reviewer noted, if Rspo3 mRNA expressions in organs other than the liver are increased or decreased in our murine models, it may be difficult to clarify whether the expression changes are reflected by increased or decreased endogenous Rspo3 in each organ or would be exogenous Rspo3 expressions reflecting adenovirus administration. Therefore, by using quantitative RT-PCR, we examined Rspo3 mRNA expressions in other organs, such as white and brown adipose tissues and skeletal muscle, of obese Rspo3-mice, which are not significantly altered as compared to their expressions in control mice (S4A Fig).

In general,, the adenovirus delivery system used in this study is regarded as the established machinery, based upon previous studies including ours (Ishigaki et al, *Diabetes* 54, 322-32, 2006, Uno et al, *Science* 312, 1656-9, 2006 and Imai et al, *Science* 322, 1250-4, 2008) and those of others, in which administration of several types of adenovirus through tail veins of mice resulted in selective gene delivery into the liver.

Therefore, it is confirmed that all phenotypes described in the original manuscript are actually due to the distant effects mediated by an inter-organ communication system originating in the Rspo3-overexpressing liver.

These new data have been added to the revised manuscript (S4A Fig and page 13, line 22 to page 14, line 1).

2. The authors suggest RSPO3 is mainly expressed in zone3 hepatocytes, whereas many previous studies collectively suggest that WNT ligands, including RSPO3, are mostly expressed in central vein endothelial cells and neighboring sinusoidal endothelial cells. The authors should provide better staining evidence with co-stainings for hepatocyte markers and endothelial cell markers, as well as ISH stainings for RSPO3 with cell type co-stainings to clarify this point. The relatively low reduction in RSPO3 levels in the AlbCreER-induced RSPO3 KO further suggests that the majority of RSPO3 is generated elsewhere.

We appreciate the reviewer’s important comments. As the reviewer noted, many previous studies have indicated that Wnt ligands and Rspo3 are mostly expressed in central vein endothelial cells and neighboring sinusoidal endothelial cells. In the original manuscript describing this study, the immunofluorescence analysis of endogenous Rspo3 in the liver indicated that endogenous Rspo3 is also expressed in hepatocytes with a tendency for greater expression in the pericentral zones (zone3) rather than the periportal zones (zone1) (S1 Fig and S2 Fig). Therefore, to more clearly identify the location of Rspo3 expression in the liver, we performed additional experiments.

First, we conducted immunofluorescence analyses of the liver specimens using antibodies against Rspo3 and HNF-4α, as a hepatocyte marker, and CD31 (PECAM-1), as an endothelial marker, and CK19, as a sinusoidal endothelial marker. As for investigating the relationship between Rspo3-positive cells and central vein endothelial cells, we performed co-immunofluorescence staining of anti-Rspo3 and anti-CD31 antibodies. As for investigating the relationship between Rspo3-positive cells and hepatocytes or sinusoidal endothelial cells, we performed immunofluorescence staining of essentially identical specimens with each antibody, thereby clearly representing the associations of hepatic localizations between Rspo3-positive cells and these marker-positive cells to a degree equivalent to that of the co-staining methods. The results of HNF4α staining indicate that endogenous Rspo3 is actually expressed in HNF4α-positive hepatocytes much more in zone3 hepatocytes than in zone1 hepatocytes (Fig 1E). The results of CD31 co-staining and CK19 staining indicate that endogenous Rspo3 is also expressed in central vein endothelial cells, but not in neighboring sinusoidal endothelial cells. However, according to immunofluorescence analyses, Rspo3 expression in central vein endothelial cells seems to be very minor (Fig 1E). These new data have been added to the revised manuscript (Fig1E, page 9, line 21 to page 10, line 6).

Next, we performed the newly developed technique of RNA *in-situ* hybridization, i.e., RNA-Scope, which allows selective detection of endogenous and adenovirus-delivered Rspo3 mRNA expressions by using a specific probe targeting Rspo3 mRNA, and we thereby assessed the locations in the liver of Rspo3 mRNA expression and quantified the degree of Rspo3 mRNA expression by counting the numbers of Rspo3-positive signals (dots). These results indicated that endogenous Rspo3 mRNA is expressed much more in the pericentral than in the periportal zones and that it is detected in central vein endothelial cells as well as hepatocytes (Fig 1H). Furthermore, the expression of endogenous Rspo3 mRNA in the pericentral zones is increased in the postprandial state as compared to the fasting state in lean murine models, a tendency not observed in obese mice (Fig 1I). This so-called spatiotemporal regulation of endogenous Rspo3 mRNA expression is consistent with the concept of Rspo3 protein expression shown by the IF analyses described in the original manuscript (Fig 1F). These new data have been added to the revised manuscript (Fig 1H and Fig 1I, page 10, line 14 to page 11, line 3).

In addition, the RNA-Scope study confirmed that adenovirus-induced Rspo3 mRNA expression in the livers of obese Rspo3 mice is increased as compared to that of control mice (S4B Fig), and to a degree essentially the same as that shown by the original results obtained employing quantitative RT-PCR (Fig 3A). These new data have been added to the revised manuscript (S4B Fig, page 14, lines 1-3).

Again, the RNA-Scope study confirmed that endogenous Rspo3 mRNA expression in the livers of iLiRspo3KO mice is decreased as compared to that of control mice (S14A Fig) and that the degree is comparable to that shown by the original results obtained using quantitative RT-PCR (Fig 6A). However, by measuring the ratio of Rspo3 protein volumes to the internal loading standard in western blotting, it was shown that the hepatic Rspo3 protein decrease in iLiRspo3KO mice approaches almost 50% of that seen in control mice (Fig 6A). These new data have been added to the revised manuscript (S14A Fig and Fig 6A, page 24, lines 5-9).

Putting these newly identified data together indicated that endogenous Rspo3 mRNA and protein are actually expressed in hepatocytes of the pericentral zones in a manner that is spatiotemporally regulated and functions as a modulator in maintaining metabolism. Importantly, these physiological processes are disturbed in obesity.

3. On page 11, lines 7-10 the authors describe upregulation of zone3 and reduction of zone1 metabolic enzymes upon RSPO3 overexpression. This finding is not novel since the role of RSPO3 in metabolic zonation (Rocha et al, Cell Reports 2015) and reprogramming of zone1 into zone3 hepatocytes by RSPO treatment, or deletion of ZNRF3/RNF43, (Sun et al, Cell Stem Cell 2021) have been previously shown. The authors should end that section by referencing these previous findings. Although the authors find exciting new data suggesting RSPO3-related interesting extrahepatic phenotypes, impaired metabolic zonation in patients is major challenge for such a therapy and should be discussed in detail.

As the reviewer suggested, we ended this section by referencing these previous reports (Rocha et al, *Cell Reports* 2015; Sun et al, *Cell Stem Cell* 2021). Furthermore, in accordance with the reviewer’s helpful comments, we now discuss the possibility of clinical applications of targeting the Rspo3-mediated inter-organ communication system in patients with diabetes and/or obesity in the discussion (page 32, lines 4-17).

4. The authors write in the last sentence on page 11 that RSPO3 slightly increased liver weight, whereas Figure 2E show almost doubled liver weight. RSPO injections increased hepatocyte proliferation and liver weight (Planas-Paz et al, Nature Cell Biology 2016 and Sun et al, Cell Stem Cell 2021) and combined of ZNRF3/RNF43 resulted into uncontrolled hepatocyte proliferation and hepatic tumor formation (Sun et al, Cell Stem Cell 2021). The author have to assess proliferation in their model by Ki67 staining, or if possible by EdU incorporation. Please note the Adenoviral injections induce inflammation which can mask the proliferative effect, so later timepoints (min. 2 weeks post injection) should be assessed. Importantly, the authors have to discuss this caveat associated with potential RSPO3-related therapies.

We agree with the reviewer’s comment and speculation that Rspo3 induction increases hepatic proliferative capacity, which would also be compatible with these previous reports. As the reviewer suggested, to assess the proliferative capacity in the livers of obese Rspo3 mice, we performed Ki-67 staining using their livers obtained 23 days after adenovirus administration to exclude any side effects of hepatic inflammation induced by adenovirus infection. In addition, we examined hepatic Ki-67 mRNA expressions of lean and obese Rspo3 mice by quantitative RT-RCR on days 7 and 23 after adenovirus administration.

First, Ki-67 staining analysis indicated that hepatic Rspo3 induction slightly increases the intensity of Ki-67 immunofluorescence, leading to moderate up-regulation of the proliferation of hepatocytes by Rspo3 induction (S4H Fig).

Second, the data obtained by quantitative RT-PCR indicated that hepatic Ki-67 mRNA expression is increased in lean or obese Rspo3 mice on day 23 by almost 5- or 2-fold as compared to that of control mice, respectively (S3D Fig or S4I Fig, respectively). The results obtained from obese Rspo3 mice are considered to be compatible with the results of Ki-67 immunofluorescence staining analysis (S4H Fig).

These newly identified results indicate that hepatic Rspo3 induction apparently results in a slight up-regulation of the proliferative capacity of hepatocytes after prolonged adenovirus administration. However, the significance of this proliferative activity of hepatocytes needs to be clarified in a future study, which it is hoped would lead to a better understanding of Rspo3 applications for patients suffering from diabetes and/or obesity.

These new data have been added to the revised manuscript (S3D Fig, page 12, line 22 to page 13, line 3, S4H Fig and S4I Fig, page 15, lines 2-5, respectively).

5. The authors observed that many metabolic genes are deregulated upon increased Rspo3 expression. Instead of picking single candidate genes, they should use their RNA-seq data to get an overall picture (perform GSEA or GO term analysis) and support their hypotheses on the regulation of entire pathways. Complete lists of PC and PP metabolic genes should be shown as well in line with the previous comment.

As the reviewer suggested, we performed Gene ontology (GO) analysis of our original RNA-Seq data by using the web-accessible program DAVID (Database for Annotation, Visualization, and Integrated Discovery), which integrates functional genomic annotations. The results obtained by this GO analysis have now been added to the supporting information as two bubble plots of up-regulated GO terms (S7 Fig). These results confirm that hepatic Rspo3 induction in obese mice actually changes several pathways associated with the GO terms of biological processes, cellular components and molecular functions. Most notably, the results of down-regulated GO terms confirm that metabolic pathways such as glucose and lipid metabolism are mainly decreased in obese Rspo3 mice (S7 Fig, page 15, lines 9-11). In addition, according to careful review of our original RNA-Seq data, PC metabolic genes such as Rnase4, Cyp2a4, Cyp1a2, Rdh9, Cyp7a1, Gstm3, Avpr1, and Oat are increased. On the other hand, PP metabolic genes such as Arg1, Gls2, Pck1, Aldh1b1, and Sds are decreased (S1 Table). All of these results confirm that hepatic Rspo3 induction has the potential to change and reprogram hepatocytes from those rich in the periportal features to those rich in the pericentral ones (page 14, lines 10-15).

On the other hand, we compared the staining patterns between Rspo3 IF, as a marker of endogenous plus exogenous Rspo3, and adenovirus-derived ZsGreen1 fluorescence, as a marker of exogenous Rspo3, by means of the liver specimens from obese Rspo3 mice. Surprisingly, these comparisons indicate that adenovirus-derived Rspo3 is expressed much more locally in the pericentral zones rather than the periportal zones (S4G Fig), leading to the expansion of Rspo3-expressing hepatocytes toward the periportal zones. Therefore, these results support the concept that hepatic Rspo3 induction can change the characteristics of entire hepatocytes from those of periportal into pericentral hepatocytes (page 14, line 15 to page 15, line 2).

6. In Fig3A they claim that Rspo3 expression via adenoviral administration may be lower in obese than in lean mice. This comparison is not shown, thus the authors should either add a corresponding graph or remove this sentence.

As the reviewer noted, in the original manuscript we did not present the comparison between hepatic Rspo3 expression of lean Rspo3 mice and that of obese Rspo3 mice. In fact, we showed the difference in Rspo3 expression between obese Rspo3 mice and obese or lean control mice. Therefore, as suggested, we corrected this sentence to “Systemic infusion of Rspo3 adenovirus resulted in selective transgene expression in the liver (1.85-fold increase in protein levels as compared to obese control mice), to the same or a slightly lesser degree than endogenous Rspo3 expression in lean control mice (0.74-fold) (Fig 3A)” (page 13, lines 19-22).

7. Fig S4 shows a metabolomics analysis. Although the overview map may be helpful to establish the connections between metabolites for the reader, it is more important to convey the message which pathways are up/downregulated. Therefore, the authors should consider exchanging/adding a heatmap instead and group metabolites by pathway. Also here, metabolic processes related to PP hepatocytes should be shown to check whether RSPO3 expression impaired PP hepatocyte metabolism via reprogramming of PP hepatocytes into PC hepatocytes.

As the reviewer noted, the overview heatmap of our metabolomics analysis and the changes in group metabolites by pathway are considered to be helpful for understanding the connections between metabolites altered by Rspo3 induction in obese mice. Therefore, we added the original overall metabolomics analysis to the supplemental figures (S5 Fig and S1 Excel).

8. Fig3D should highlight Akt phosphorylation. However, loading controls and quantification are missing (same for Fig3E, as well as for many other WB analyses!). This must be added for all WB. Additionally, Foxo1 translocation can only be claimed when cytoplasmic levels are shown together with nuclear levels. Otherwise, it might be just increased expression. In addition, the authors conclusions must be toned down (decreased gluconeogenesis and glucose utilization in TCA are not shown by the authors).

As the reviewer noted, Fig 3D presented the results of insulin signaling tests, in which we performed the immunoprecipitation studies by using Akt antibody. With this method, we first immunoprecipitated the Akt proteins with the same amount of this antibody from insulin-acting organs such as the liver, adipose tissues and skeletal muscles after insulin stimulation, followed by the immunoblotting of these proteins with phospho-Akt-antibody and Akt-antibody itself. Therefore, in the insulin signaling tests, the results of immunoblot against Akt-antibody are regarded as loading controls. In addition, we measured the ratio of phospho-Akt volumes to Akt volumes in western blotting (Fig 3D), thereby confirming the insulin signaling increases in several organs of obese Rspo3 mice.

In Fig. 3E, we used HDAC1 protein as a loading control for nuclear extracts, while we used GAPDH protein as a loading control for cytoplasmic extracts.

As the reviewer suggested, we examined Foxo1 protein expression in the cytoplasm of hepatocyte extracts of obese Rspo3 mice, indicating that hepatic Rspo3 induction does not alter Foxo1 expression in the cytoplasm (Fig 3E). Therefore, it is suggested that Foxo1 expression in the nucleus decreases (Fig 3E) and that the degree of Foxo1 exclusion from the nucleus to the cytoplasm seems to be increased in these Rspo3 mice. On the other hand, as previously reported (*Cell metabolism* 6, 208-16, 2007, etc.), selective knockdown of hepatic Foxo1, meaning loss of both the nucleus and the cytoplasm, deceased hepatic gluconeogenesis and improved obesity-associated glucose intolerance and insulin resistance. Taking these reports and the newly obtained data into consideration, the interpretation in the original manuscript that hepatic Rspo3 induction decreased nuclear Foxo1 expression and resultantly decreased gluconeogenesis and increased glucose utilization in TCA would not necessarily be diminished.

9. In Fig S5 the authors make assumptions about minor changes of PPARδ and CPT1a (lower than 1.5-fold, and not even confirmed on protein levels). If this remains the only evidence, the authors must tone down their conclusions.

As the reviewer noted, the changes of PPARδ and Cpt1a did seem to be moderated in the RNA-Seq analysis in Fig S8. On the other hand, the quantitative RT-PCR examination described in the original manuscript indicated that hepatic Rspo3 induction in obese mice increased the expressions of PPARδ and Cpt1a by about 1.7- and 1.5-fold as compared to those of control mice, respectively. In addition to mRNA expressions, we examined the changes in PPARδ and Cpt1a protein levels in the liver by western blotting, and the results indicated the up-regulations of these PPARδ and Cpt1a proteins by about 2.0- or 2.4-fold as compared to that of control mice, respectively. According to these new data, the interpretation in the original manuscript that hepatic Rspo3 induction increased PPARδ and Cpt1a expressions, resulting in individual effects following their signaling pathways, would not appear to be weakened.

These new data have been added to the revised manuscript (Fig 3K, page 16, line 20 to page 17, line 2).

10. The sentence after citation 30" thereby leading to the restoration of adrenergic signaling, mitochondrial biogenesis and skeletal muscle mass [31], which had been impaired in the setting of obesity" is an overstatement considering that the authors only refer to increased β2AR expression and resultant PGC1α expression in the muscle. In general, the entire following sections contains a lot of discussion and correlative findings (i.e. referring to PC18:0/18:1). Either this should be moved into the discussion part, or alternatively, the authors should add some more supportive data here.

As the reviewer suggested, we moved this section mainly into the discussion portion (Page 29, lines 10-16). On the other hand, we examined circulating phosphatidylcholine (PC) levels in obese Rspo3 mice, indicating that hepatic Rspo3 induction increases the PC synthesis in the liver and significantly up-regulates circulating PC levels in obese Rspo3 mice, by 1.24-fold, as compared to control mice (Fig 4J).

In addition, re-examination of hepatic vagotomy (HV) experiments surprisingly indicated that HV almost completely blocks Rspo3-induced PGC1α and PPARα expression increases in skeletal muscles (Fig 5J). Therefore, a complicated mechanism exists whereby skeletal muscle PGC1α and PPARα expressions are under the control of the neuronal communication, while fatty acid utilization in skeletal muscle through PPARα is controlled by non-neuronal communication. Thus, we now present this supportive information in the results section (Fig 4J, page 21, lines 13-16, Fig 5J, page 23, lines 6-7, respectively) and also elaborate on the complicated mechanism in the discussion section (Page 29, lines 10-16).

11. The authors claim that HV also almost completely blocked Rspo3-induced BCAT2 and BCKDH expression. However, for FigS8D significances are not shown when comparing Rspo3 conditions between Sham and HV as done for literally every other gene. Given the error bars for BCAT, this raises doubts regarding the drawn conclusion. Can the authors show the graph displaying single samples at least to the reviewer?

We again conducted the HV experiments and re-analyzed BCAT2 and BCKHD mRNA expressions in BAT of SO- and HV-Rspo3 mice. The results obtained indicated that these expressions are decreased in SO-Rspo3 mice but not in HV-Rspo3 mice and the comparisons of these expressions between SO- and HV-Rspo3 mice revealed significant differences (P < 0.05). These new data have been added to the revised manuscript (S12F Fig, page 23, lines 1-3).

Minor comments:

1. Most histological staining pictures are too small, quality and size should be increased. CVs and PVs should be labelled.

As the reviewer suggested, we increased the size and quality of histological staining pictures and the CVs and PVs are now well labelled.

2. The word "ironically" must be removed from the abstract.

As the reviewer suggested, we removed the word “ironically” from the abstract.

3. What do the authors mean by the term "feeding behavior"? The exact timepoint of analysis after feeding should be stated in the text.

This term indicates the timepoint of analysis such as the fasted state and the fed state. The former indicates a 10-hour fasted state from 9:00 a.m. to 7:00 p.m. and the latter the 13-hour period after feeding from 8:00 p.m. to 9:00 a.m.

4. The authors use the term "Rspo3 supplementation" throughout the text. However, supplementation is rather used in a dietary context and should be exchanged for a more appropriate term (eg use increased Rspo3 expression).

As the reviewer suggested, we corrected the term “Rspo3 supplementation” to “Rspo3 induction” to denote the increase in hepatic Rspo3 expression through the adenovirus gene delivery system.

5. The term "as previous report indicating" used several times throughout the text is grammatically incorrect.

As the reviewer suggested, we corrected or eliminated this term throughout the manuscript.

6. Quantification for number of lipid droplets is missing (Fig.3H).

As the reviewer suggested, we examined TG contents in the liver, the amounts of which indicated that hepatic Rspo3 induction markedly decreases TG levels in the livers of obese mice, while hepatic suppression of Rspo3 increases TG levels in the livers of iLiRspo3KO mice (Fig 3I and Fig 6L).

7. FigS5 heatmap should be adjusted to color-blind standards.

As the reviewer suggested, we adjusted the RNA-Seq heatmap to color-blind standards and replaced S5 Fig of the original manuscript with a new figure, S8 Fig.

8. The graphical abstract in Fig7 is too complicated and should be reduced to the major findings. Alternatively, this extended version could be kept in supplement and a "reduced" version presented in the main figures.

As the reviewer suggested, we redesigned the graphical abstract which contains only the major findings and makes the significance of this study more understandable, as shown in the new Fig 7. We then placed this rearranged version in the main figure (Fig 7) and described the extended version in the supplemental figure (S15 Fig).

Reviewer #2, Costas Christodoulides (note, Reviewer 2 has signed this review):

In this paper, Uno and colleagues investigate the role of hepatic Rspo3 on systemic metabolism using gain- and loss-of-function approaches. Consistent with previous reports, they show that adenovirally mediated, hepatic over-expression of Rspo3 alters liver metabolic zonation (PMID: 26655896) and it is further associated with reduced weight gain and improvements in glucose tolerance and systemic insulin sensitivity in both lean and ob/ob mice. In ob/ob mice, hepatic Rspo3 over-expression also ameliorated hepatic steatosis. In another mouse model of genetic obesity (KK-Ay), hepatic vagotomy appeared to partially reverse the beneficial effects of hepatic Rspo3 over-expression on adiposity and glucose tolerance. Reciprocally, tamoxifen-induced, hepatic knockdown of Rspo3 led to impaired glucose tolerance and systemic insulin sensitivity, as well as, increased hepatic steatosis in chow-fed animals.

Based on these findings, the authors postulate that "targeting hepatic metabolic zonation by Rspo3 may provide new therapeutic strategies against diabetes and the metabolic syndrome".

Strengths of the manuscript include that many of the findings are novel and are supported by evidence from both gain- and loss-of-function experiments conducted in vivo and ex vivo. Nonetheless, I have several comments/criticisms as listed below:

Major criticisms:

1. The authors examine only the short-term effects (one week) of hepatic Rspo3 gain- and loss-of-function on adiposity and systemic metabolism (see also comment 6).

As the reviewer noted, we agree regarding the importance of the long-term effects of hepatic alteration of Rspo3 expression. Therefore, we examined the long-term effects of hepatic Rspo3 gain- and loss-of-function, in the former for 3 weeks after adenovirus administration and in the latter for one month or more after tamoxifen administration.

First, hepatic Rspo3 induction in lean mice maintains the elevated glucose tolerance and insulin sensitivity up to day 20 (almost 3 weeks) after adenovirus administration (S3E Fig and S3F Fig). In addition, hepatic Rspo3 induction in obese mice also improves obesity-associated glucose intolerance and insulin resistance on day 20 (3 weeks) after adenovirus administration (S10D Fig and S10E Fig), with less body weight gain (LacZ 9.446±1.818g, Rspo3 6.833±1.335g, *P* < 0.05, *n*=5) and decreased epidydimal WAT weight (LacZ 0.064±0.008g, Rspo3 0.055±0.005g, *P* < 0.05, *n*=5) during this relatively long period. Therefore, the beneficial effects on glucose metabolism and body composition persist for a relatively long duration.

Second, hepatic Rspo3 suppression under lean conditions also leads to deterioration of glucose tolerance and insulin sensitivity 2 months (8 weeks) after tamoxifen administration (S14F Fig). Of course, the impact of this is less than that observed in the short-term experiments (Fig 6C and 6D). These results indicate that suppression of hepatic Rspo3 expression exerts a permanent but gradually weakening non-beneficial influence on the homeostatic regulation of systemic glucose metabolism.

2. Based on gene expression analyses, the hepatic over-expression of Rspo3 achieved with adenoviral transductions was very high (10-20x) and was associated with a significant increase in liver size e.g., almost doubling in chow-fed animals (fig. 2e).

As the reviewer noted, in the original manuscript, hepatic over-expression of Rspo3 through the adenovirus delivery system reached nearly 10- to 20-fold mRNA levels as compared to that of control mice. However, we examined the ratio of Rspo3 protein levels as compared to that of control mice, revealing that hepatic Rspo3 induction in lean mice increases its protein level by 1.45-fold as compared to that of control mice (Fig 2A). In addition, hepatic Rspo3 induction in obese mice also increases its protein level by 1.85-fold as compared to that of control mice, reaching nearly the protein level observed in lean control mice (0.74-fold) (Fig 3A). These results indicate that although hepatic over-expression of Rspo3 via our method results in an approximately 10- to 20-fold increase of its mRNA levels, the protein level increase, especially in obese mice, is similar to that of lean control mice. This observation is considered to be reasonable in that hepatic Rspo3 induction in obese mice compensates for the pathophysiological decrease in Rspo3 expression due to obesity.

3. The authors show in figure 1, that Rspo3 circulates in plasma and furthermore, that it's circulating levels are up-regulated in the fed state and dysregulated in obesity. Yet, they fail to show any data on circulating Rspo3 levels in their gain- and loss-of-function animal models. Additionally, they fail to consider that any of the effects of hepatic Rspo3 on weight gain and systemic metabolism could be mediated by endocrine actions of Rspo3, which I find quite puzzling.

As the reviewer suggested, we examined the alteration of circulating Rspo3 levels in obese Rspo3 mice at several time points including the early stage (on day3) and the late stage (on day7) after adenovirus administration. The results indicated that hepatic Rspo3 induction increases the circulating Rspo3 levels only on day3, while there is no difference in the circulating Rspo3 levels between Rspo3 and control mice on day 7 after adenovirus administration (S4C Fig, page 14, lines 3-5). Again, we examined the alteration of circulating Rspo3 levels in lean Rspo3 mice at the middle stage (on day5) and the late stage (on day7) after adenovirus administration. There were no differences in the circulating Rspo3 levels between Rspo3 and control mice on days 5 and 7 after adenovirus administration (Rspo3 vs control mice: 6.071±2.235 vs 4.995±3.411 on day5, 4.044±0.339 vs 4.014±0.78 on day7). This phenomenon indicates that circulating Rspo3 protein is temporarily increased, i.e., in the short term, but that it promptly returns to a level similar to that observed in the control mice. Taking the long-term effects of gain-of function (described in Major criticism 1) and the results of hepatic vagotomy experiments into consideration, the remote effects derived from hepatic Rspo3 induction are not due to short-term alterations of circulating Rspo3 protein but rather to the action of Rspo3 as a mediator of inter-organ neuronal communication originating in the liver other than as an endocrine protein.

4. Another major limitation, is that the authors failed to measure food intake and energy expenditure in any of their mouse models. This makes it difficult to interpret and draw firm conclusions from their findings.

We appreciate the reviewer’s important comments. As the reviewer suggested, we examined the amounts of food intake employing gain-of-function or loss-of-function models for relatively long periods after adenovirus or tamoxifen administration, respectively. In gain-of-function models, i.e., obese Rspo3 mice, hepatic Rspo3 induction decreased the accumulated amounts of food intake for 7 or 14 days, by almost 10 g (27.0 g vs 37.7 g in control mice) or 18 g (56.8 g vs 74.4 g in the control mice), respectively (Fig 3N). On the other hand, in the loss-of-function model, i.e. iLiRspo3KO mice, suppression of hepatic Rspo3 did not alter the cumulative amounts of food intake for 7 or 19 days from 4 weeks after tamoxifen administration (25.9 vs 28.1 g in the control mice, or 75.7 g vs 72.1 g in the control mice, respectively) (S14B Fig). These results indicate that hepatic Rspo3 induction decreases the amount of food intake in obese and hyperphagic mice, but that suppression of hepatic Rspo3 does not alter that in lean and non-hyperphagic mice.

Furthermore, we examined energy expenditure changes, such as O_2_ consumption (VO_2_) and the respiratory quotient (RQ) ratio, for gain-of-function or loss-of-function models after adenovirus or tamoxifen administration, respectively. Hepatic Rspo3 induction in obese mice decreases VO_2_ and thereby decreases RQ (Fig 4G), raising the possibility that it restores overdrive of energy expenditure against obesity development and alters the balance of energy source use shifting toward utilization of lipids rather than that of carbohydrates. On the other hand, suppression of hepatic Rspo3 increases VO_2_ and thereby increases RQ (Fig 6H), forming a mirror image of the results just obtained with the Rspo3 overexpression models. Therefore, modulation of hepatic Rspo3 expression results in the regulation of energy expenditure with a balance between the utilization of energy sources such as carbohydrates, lipids, and amino acids.

These new data have been added to the revised manuscript (Fig 4G, page 19, lines 15-22, Fig 6H, page 25, lines 4-7, respectively).

5. The abstract and results sections should be factual. Yet, they contain a lot of speculation. For example, the authors claim that a remote, lipid mediated, liver-muscle communication system mediates some of the beneficial effects of hepatic Rspo3 on systemic metabolism and improves skeletal muscle quality. However, the data presented fail to directly demonstrate such an axis. Specifically, the authors do not demonstrate either increased circulating phosphatidylcholine levels in their Rspo3 gain-of-function animal models, or direct effects of phosphatidylcholine on skeletal muscle gene expression, or that their animals have e.g., increased exercise tolerance. Thus these comments are best included as speculation in the discussion. There are multiple other similar examples in the results section.

As the reviewer suggested, we moved the section pointed out above mainly into the discussion, where it is now presented as speculation (Page 29, lines 10-16). On the other hand, we examined circulating phosphatidylcholine (PC) levels in obese Rspo3 mice, which indicated that hepatic Rspo3 induction increases the PC synthesis in the liver and significantly up-regulates circulating PC levels in obese Rspo3 mice, by 1.24-fold, as compared to control mice (Fig 4J).

In addition, re-examination of the hepatic vagotomy (HV) experimental results surprisingly indicated that HV almost completely blocks Rspo3-induced PGC1α and PPARα expression increases in skeletal muscle (Fig 5J). Therefore, a complicated mechanism exists by which skeletal muscle PGC1α and PPARα expressions are under the control of neuronal communication, while fatty acid utilization in skeletal muscle through PPARα is under the control of non-neuronal communication. Thus, we now present this supportive information in the results section (Fig 4J, page 21, lines 13-16, Fig 5J, page 23, lines 6-7, respectively) and also include the existence of the complex underlying mechanism in the discussion (Page 29, lines 10-16).

6. The authors do not provide any direct evidence that the activity of the sympathetic nervous system in adipose tissue is altered in response to changes in hepatic Rspo3 expression, yet this is one of their main conclusions.

As the reviewer suggested, we examined the norepinephrine (NE) levels of adipose tissues in obese SO- and HV-Rspo3 murine models. Hepatic Rspo3 induction decreases the NE levels in BAT of SO-models but this down-regulation of NE levels in BAT was almost completely cancelled in the HV experiments (Fig 5I). In addition, hepatic Rspo3 induction increases the NE levels in epidydimal WAT of SO-models but this up-regulation of NE levels in WAT was almost completely blocked in the HV experiments (Fig 5I). These results are considered to address the reviewer’s important concerns about the need for direct evidence that the activity of the sympathetic nervous system in adipose tissues is altered.

These new data have been added to the revised manuscript (Fig 5I, page 23, lines 3-5).

7. Rspo over-activation has been linked with cancer e.g., RSPO3 gene fusions which are associated with increased RSPO3 expression have been identified in colon cancer patients (PMID: 34663878). Consistently, transgenic mice with increased circulating Rspo levels displayed massive intestinal enlargement in mice (PMID: 16109882, 16357527). On the other hand, Rspo are important for stem cell maintenance and proliferation (PMID: 22617424). Hence, it is highly likely that manipulation of hepatic Rspo3 expression for metabolic disease prevention/treatment purposes will have a very narrow therapeutic window (if at all possible).

We agree with the reviewer’s comment and speculation that application of hepatic Rspo3 modulation to clinical usage in metabolic disease patients may have a narrow therapeutic window according to previous reports. In carefully assessing these previous avenues of research, it is noteworthy that Rspo3 expression in each organ or circulating Rspo3 was found to act as the modulator of the proliferative capacity in each of the respective organs.

On the other hand, in the present study, we focused on the selective function of Rspo3 as regards liver morphology, leading to the identification of a new inter-organ neuronal communication system. Therefore, if we can develop a technical method selectively activating hepatic Rspo3, but not Rspo3 in other organs, and it might be possible to reconstitute the metabolic zonation disturbed in obesity. If this could be achieved, it might be helpful to apply such a new role of hepatic Rspo3 to patients suffering from metabolic diseases. Of course, to meet this goal, further study and a new knowledge based on the results are required, as now described in the discussion (page 32, lines 4-17).

Specific points:

1. Could energy repartitioning to the growing liver, and potentially growing intestines, contribute to the beneficial effects of hepatic Rspo3 over-expression on systemic metabolism?

We agree with the reviewer’s important insight that energy repartitioning to a wide range of growing organs may contribute to the effects of hepatic Rspo3 induction on systemic nutrient metabolism. This observation is considered to be connected to the future of innovative studies in metabolism biology. However, the precise mechanisms by which energy sources are properly redistributed to each organ as needed were not clarified in the present study. In addition, the relationship between the redistribution of energy sources like this and the effects of hepatic Rspo3 modulation on systemic metabolism were, likewise, not identified at the present stage. Therefore, to resolve this problem, further study and a new knowledge based on the results are required.

2. The authors claim that "Rspo3 supplementation restores the hyperactivated metabolic rates of BAT-derived adaptive thermogenesis against the development of obesity". However, they do not provide any evidence that BAT is overactive in their ob/ob mice. Secondly, they do not examine any direct effects of Rspo3 on brown adipocyte gene expression. Finally, it is difficult to believe that reduced BAT activity contributes to the reduced weight gain and improved systemic metabolism of mice with hepatic over-expression of Rspo3.

As the reviewer suggested, to investigate whether BAT as well as epidydimal WAT of ob/ob mice is overactive against obesity development, we examined the expression of UCP1 mRNA, one of the genes associated with adaptive thermogenesis, in adipose tissues of lean and several obese murine models, followed by a comparison of these gene expressions between these lean and obese mice. The results indicated that UCP1 mRNA expression in BAT of ob/ob mice is increased by 50% as compared to that of lean mice, while its expression in WAT of obese mice is surprisingly increased by 5.5-fold as compared to that of lean mice (S11A Fig). In addition, in another type of obese mouse, KK-Ay, UCP1 mRNA expressions in both BAT and WAT are increased by 6.6- and 1.9-fold as compared to those of control mice, respectively (S11B Fig). Furthermore, we compared the difference in energy consumption (VO_2_) between the lean control mice and ob/ob mice, which demonstrates the VO_2_ of obese mice to be higher (by 1.25-fold) than that of lean control mice (S11C Fig). Taking these data into consideration, the insight that energy expenditure in obese mice, as the sum of adaptive thermogenic signals from each of the adipose tissues, is activated against obesity development is considered to be reasonable (page 19, line 22 to page 20, line 2).

In the original manuscript, we did not examine the direct effects of Rspo3 on gene expressions in brown adipocytes, because the circulating Rspo3 protein did not increase in obese Rspo3 mice as compared to the level in control mice for a relatively long period. These data, not described in the original manuscript, have now been added to the revised manuscript (S4C Fig, page 14, lines 3-5).

Although it may be difficult to show that reduced BAT activity contributes to the reduction of weight gain and the improvement of systemic metabolism with hepatic Rspo3 induction in obese mice, the cumulative impact of the remote effects on several organs, derived from hepatic Rspo3 induction, improves metabolic homeostasis throughout the body.

3. I would like to see the effects of hepatic vagotomy on animal weights in figure 5. It is also curious that figure 5D shows data on the ratio of epiWAT/body weight in the vagotomised animals whereas figures 2I and 4D compare the effects of hepatic Rspo3 over-expression on epi WAT weight uncorrected for total body weight.

As the reviewer suggested, we examined the effects of hepatic vagotomy on body weight changes by comparing the difference between body weights of SO-LacZ mice and those of HV-LacZ mice. The difference in body weight gain for 7 days after LacZ adenovirus administration was not significant, when considering the results that the body weight gains of SO-LacZ mice or HV-LacZ mice were 4.3 g and 4.0 g, respectively (S12A Fig, page 22, lines 9-11). Therefore, HV itself does not influence body weight gain in mice after this treatment.

In addition, as the reviewer suggested, we also now present data on the ratio of epidydimal WAT weight/body weight in Fig 2I and Fig 4D.

4. Did hepatic vagotomy have any effects on WAT Ucp1 and Pgc1a gene expression or on systematic insulin sensitivity as assessed by insulin tolerance tests?

As the reviewer suggested, we examined whether HV has any effect on β3AR, UCP1 and PGC1α gene expressions in WAT by quantitative PCR. The results obtained indicate HV to almost completely cancel the Rspo3-induced up-regulations of β3AR, UCP1 and PGC1α in epidydimal WAT (Fig 5D).

Furthermore, we performed insulin tolerance tests on SO-Rspo3 mice and HV-Rspo3 mice. The results indicate that hepatic Rspo3 induction improves insulin resistance in SO-mice but it tends to slightly improve insulin resistance in HV-mice. However, the degree of the insulin resistance improvement differs between SO-Rspo3 and HV-Rspo3 mice, indicating that HV partially or almost completely blocks the Rspo3 induced improvement of insulin resistance in obese mice (Fig 5L). This phenomenon is reasonable given that HV completely blocks the remote effects mediated by inter-organ communication while having no influence on the local effects in the liver.

These new data have been added to the revised manuscript (Fig 5D, page 22, lines 18-21, Fig 5L, page 23, lines 11-20, respectively).

5. Did Rspo3 knockdown have any effects on liver size, weight gain, or epididymal WAT weight?

As the reviewer suggested, we now present the effects on body weight gain, liver weight and epidydimal WAT weight. Suppression of hepatic Rspo3 does not influence the weight of either the liver or epidydimal WAT, nor does it alter body weight gain, as compared to the values of the control mice (S14B Fig, S14C Fig, and S14G Fig).

6. Ideally, the authors should quantify the hepatitic lipid content in animals with Rspo3 knockdown and over-expression.

As the reviewer suggested, we examined the hepatic TG contents of gain-of-function and loss-of-function models after adenovirus and tamoxifen administrations, respectively. Hepatic Rspo3 induction in obese mice markedly decreased TG contents in the liver (Fig 3I), while suppression of hepatic Rspo3 increased TG contents in the livers of iLiRspo3KO mice (Fig 6L). These results are considered to correspond to the histological analyses in the original manuscript (Fig 3I and Fig 6L).

Other points:

1. In figure 1C, was Rspo3 expression reduced in BAT and skeletal muscle from obese mice?

Yes, endogenous Rspo3 expressions in BAT and skeletal muscle of obese mice were significantly decreased as compared to these expressions in lean control mice.

2. Is beta-catenin a WNT target gene?

Yes, beta-catenin is considered to function as one of the down-stream targets of the classical WNT pathway, as noted in previous reports (*Cell* 169, 985-999, 2017, *Nat Rev Gastroenterol Hepatol* 16, 121-136, 2019, etc.).

3. With reference to the data in figure 3E, does PDK4 primarily act in the nucleus?

PDK4 is known to act as an enzyme of the pyruvate dehydrogenase kinase pathway in the cytoplasm rather than in the nucleus. Thus, we examined the PDK4 protein expression in cytoplasm extracts of the liver from obese Rspo3 mice. The hepatic Rspo3 induction was found to decrease PDK4 expression in cytoplasm extracts of hepatocytes (Fig 3E), leading to up-regulated glucose utilization into the TCA cycle. We thus replaced the western blotting results in the original manuscript with new data obtained by western blotting of cytoplasm extracts of the liver.

Reviewer #3:

This manuscript by Uno et al reports the role of Rspo3 that is known to determine hepatic zonation in regulating glucose and energy homeostasis. They show that Rspo3 increases with feeding and this rise is attenuated with insulin resistance such as in ob/ob mice. Histologically, they show that Rspo3 is further concentrated in the periportal area and this does not occur in models of insulin resistance. Further, in metabolic tissues such as the liver or adipose tissue. Rspo gene expression progressively declines with insulin resistance such as with high fat diet feeing or in ob/ob mice. Next, they deliver Rspo3 via adenovirus and show increased expression in the liver in addition to improved metabolic parameters such as fasting insulin and glucose levels as well as gluconeogenic genes. This is done also in ob/ob mice showing similar metabolic benefit 7 days post gene delivery. They then examine the mice with deletion of Rspo3 in liver and show metabolic

exacerbation. Similar to the overexpression data vi adenovirus, deletion model is also examined after a short term of 7 and 13 days post deletion. Again, this is a short duration given the chronic nature of the diseases being examined and longer term data would be required. Overall, the data presented are interesting and they use multiple in vivo models as well as overexpression and deletion models to show the importance of Rspo3 in metabolic regulation. However, throughout the manuscript, the data interpretation is far-reaching and many of their statements are not at all supported by the data presented as follows. It is suggested that the authors more accurately describe the data without overreaching with conjecture. In particular, much of what is shown as a working model in Fig 7 is not at all shown in their data and would strongly suggest revising to only show what is well-supported by their data.

What is the difference between data presented in Fig 1B versus 1C, and why are the data different for the liver shown in B versus C?

Fig 1E needs to be shown in a lower magnification that demonstrate the zonation more clearly. The image shown in the figure does not capture the peri-central zonation convincingly.

As the reviewer noted, the quantitative RT-PCR data difference, especially for the liver, depends on the characteristics of the experimental methods and techniques employed. As shown in Fig 1B, we examined the Rspo3 mRNA levels only in the livers of lean mice and ob/ob mice. We also performed another cycle of quantitative RT-PCR experiments, in which we examined the Rspo3 mRNA levels in several insulin responsive organs of the lean mice, DIO mice and ob/ob mice, as shown in Fig 1C. Therefore, there is actually a difference in the cycle of quantitative RT-PCR experiments between Fig. 1B and Fig. 1C, accounting for the data variation in hepatic Rspo3 levels between lean and ob/ob mice, obtained in each experiment.

As the reviewer suggested, we now also present the immunofluorescence pictures at a lower magnification than that used in the original manuscript (S1 Fig and S2 Fig).

Fig 2A shows increased Rspo3 levels in gene and protein expression. The increase in protein is unclear from the western blot shown and should be quantified. The degree of increase in gene versus protein expression is not at all congruent and expression in other tissues need to be shown. Furthermore, histologic imaging needs to be shown to test whether the increased expression of Rspo3 is more enriched pericentrally as shown with endogenous Rspo3.

As the reviewer suggested, we analyzed the Rspo3 protein volumes obtained by western blotting and calculated the ratio of Rspo3 volumes to the loading control volumes. Hepatic Rspo3 induction in lean mice increased the Rspo3 volume ratio by almost 1.5-fold as compared to that of the control mice (Fig 2A). Therefore, it does not necessarily merit mention that the degree of the Rspo3 increase in gene versus protein expression lacks congruence. In addition, we examined Rspo3 mRNA expressions in other insulin responsive organs such as adipose tissues and skeletal muscle, and obtained results indicating that there is no Rspo3 expression difference in these organs between obese Rspo3 mice and control mice (S4A Fig).

Furthermore, we performed the immunofluorescence analysis of Rspo3 protein and the fluorescence analysis of ZsGreen1 protein integrated into the adenovirus vector, and visualized liver specimens into two colors, one being the red of Rspo3 IF, representing endogenous and exogenous Rspo3, and the other the green of ZsGreen1, representing exogenous Rspo3. According to this experiment, we were able to separately detect endogenous and exogenous Rspo3 and access the pattern of localization of adenovirus derived Rspo3 expression. These results indicated that, surprisingly, hepatic Rspo3 induction in obese mice increases adenovirus derived Rspo3 much more locally in the pericentral zones, rather than in the periportal zones (S4G Fig), resulting in the expansion of areas expressing Rspo3 protein toward the pericentral zones. These approaches provided a partial demonstration that hepatic Rspo3 induction enlarges the areas of hepatocytes with the characteristics of the pericentral zones, thereby reconstituting the hepatic morphology which had shown deterioration in obesity.

These new data have been added to the revised manuscript (S4G Fig, page 14, line 17 to page 15, line 2).

What is the evidence that 'hepatic Rspo3 induction exerted remote effects, i.e., the weight of epididymal WAT was significantly decreased (Fig 2I),' - they don't have data to support this statement. The effect in the adipose tissue may be from its expression in this tissue and not the distant effect of the liver, especially since they've already shown expression of Rspo3 in adipose tissue (Fig 1C). Also, in this same section, their postulation involving the sympathetic activation is completely not supported by their data and should be removed.

As the reviewer noted, in this section of the original manuscript we did not describe the precise mechanism by which, nor even whether or not, hepatic Rspo3 induction in lean mice resulted in decreased epidydimal WAT weight through inter-organ communication system. Of course, Rspo3 is detectably expressed in epidydimal WAT in murine models, but hepatic Rspo3 induction does not alter its expressions in adipose tissues (S4A Fig). On the other hand, we examined β3AR, UCP1 and PGC1α expressions in epidydimal WAT of lean Rspo3 mice, and obtained data indicating that hepatic Rspo3 induction increases these gene expressions in WAT (Fig 2J). However, in this section we do not strictly focus on the involvement of the inter-organ neuronal communication system in the regulation of WAT volume, and thus removed this speculative discussion about the organ communication mechanism from this section.

All the metabolic experiments are done 7 days post gene delivery. Given the chronic nature of the metabolic conditions such as diabetes and obesity that they are investigating, more longer term experiments need to be done as well as gene dosage effect. How did they come up with the dose of gene to be delivered. What are the expression levels of the gene delivery in other tissues such as the adipose tissue, muscle, brain or the immune cells? These are all tissues that can affect the metabolic outcome.

As the reviewer noted, we agree about the importance of the long-term effects of hepatic alteration of Rspo3 expression. Therefore, we examined the long-term effects (about 3 weeks) of hepatic Rspo3 gain-of-function after adenovirus administration. First, hepatic Rspo3 induction in obese mice also improved obesity-associated glucose intolerance and insulin resistance on day 20 (3 weeks) after adenovirus administration (S10D and S10E Fig), with the reduction of body weight gain (LacZ 9.446±1.818g, Rspo3 6.833±1.335g, *P* < 0.05, *n*=5) and decreased epidydimal WAT weight (LacZ 0.064±0.008g, Rspo3 0.055±0.005g, *P* < 0.05, *n*=5) during this relatively long period. Therefore, the beneficial effects on glucose metabolism and body composition persist for a relatively long duration.

In addition, we examined Rspo3 mRNA expressions in other insulin responsive organs such as adipose tissues and skeletal muscle, and found that there was no difference in the Rspo3 expressions in these organs between obese Rspo3 mice and control mice (S4A Fig).

In general, the adenovirus delivery system used in this study is regarded as the established machinery, based on previous studies including ours (Ishigaki et al, *Diabetes* 54, 322-32, 2006, Uno et al, *Science* 312, 1656-9, 2006 and Imai et al, *Science* 322, 1250-4, 2008) and those of others, in which administration of several types of adenovirus through the tail veins of mice resulted in selective gene delivery into the liver.

These approaches confirmed that all of the phenotypes described in the original manuscript are actually attributable to the distant effects mediated by an inter-organ communication system originating in the Rspo3-overexpressing liver.

Similar comments re duration after knockout apply as stated above. 7 and 13 days post knockout is not sufficient to gain insight into the chronic nature of the diseases being examined.

We agree with the reviewer regarding the importance of the long-term effects of hepatic alteration of Rspo3 expression. Therefore, we examined the long-term effects of hepatic Rspo3 loss-of-function for one month, or more, after tamoxifen administration. First, hepatic Rspo3 suppression under lean conditions also leads to deterioration of glucose tolerance and insulin sensitivity at 2 months (8 weeks) after tamoxifen administration (S14F Fig). Of course, the impact is less than that seen with the short-term experiments (Fig 6C and 6D). These results indicate that suppression of hepatic Rspo3 expression exerts a permanent but gradually weakening non-beneficial influence on the homeostatic regulation of systemic glucose metabolism.

For knockdown experiments, why only 20%? This needs to be explained. Is this because more knockdown would lead to severe hepatic derangements? This is an important issue that needs to be addressed and shown with more significant knockdown.

As the reviewer noted, our analysis of the degree of Rspo3 protein levels in the knockdown experiments indicated that suppression of Rspo3 expression in the present study reaches a level comparable to the decrease of its protein by almost 50 % as compared to that of control mice (Fig 6A). In general, there is known to be a difference as well as variety in the expressions between mRNA and protein levels. Thus, there appears to be a discrepancy in that the degree of Rspo3 suppression in iLiRspo3KO mice differs in mRNA levels versus protein levels. However, biologically, this is not a necessarily an incorrect phenomenon.

There are multiple inappropriate descriptions of their data or of the subject matter that they describe as follows:

Abstract - pg 4 - Rspo3 is not a 'bioactive substance' - they have manipulated gene expression using adenovirus to investigate its role - this needs to be accurately described in the abstract and throughout the manuscript.

As the reviewer suggested, we corrected the term “bioactive substance” to “bioactive protein” in the Abstract (Page 4, line 6).

When describing data in Fig 1D, they should be insulin 'responsive' tissue - , not insulin 'reactive'. Also, it's not feeding 'behaviour'. It's simply 'feeding' that would lead to metabolic abnormalities.

As the reviewer suggested, we corrected “insulin reactive” to “insulin responsive” (page 9, line 9), and “feeding behavior” to simply “feeding” (page 9, line 10).

'Hepatic Rspo3 supplementation' would not be the best way to describe their experiment. Supplementation has a nutrition connotation and should simply describe their systemic gene delivery rather than 'supplementation'. This is used throughout the section describing findings for Fig 3, which is advised to be removed.

As the reviewer suggested, we corrected the term “hepatic Rspo3 supplementation” to “hepatic Rspo3 induction” throughout the revised manuscript.

'hepatic PPARδ-to-muscle PPARα axis' - again is overstretch of data interpretation.

As the reviewer noted, the term “hepatic PPARδ-to-muscle PPARα axis” may be perceived as an extended interpretation of data in the original manuscript. However, in the revised manuscript, hepatic PPARδ protein levels in obese Rspo3 mice rose to almost double those of control mice (Fig 3K). In addition, circulating phosphatidylcholine levels as well as the synthesis of phosphatidylcholine in the liver were also increased in obese Rspo3 mice (Fig 4J). These new results suggest more strongly that hepatic Rspo3 induction up-regulates the PPARδ level and production of phosphatidylcholine in the circulation, thereby elevating the skeletal PPARα level and fatty acid utilization through this mechanism, i.e. non-neuronal inter-organ communication. Furthermore, re-examination of the HV experimental results surprisingly indicated that HV almost completely blocked Rspo3-induced PGC1α and PPARα expression increases in skeletal muscle (Fig 5J). Therefore, a complicated mechanism exists whereby skeletal muscle PGC1α and PPARα expressions are under the control of the neuronal communication, while fatty acid utilization in skeletal muscle through PPARα is under the control of the non-neuronal communication system. Therefore, we corrected the sentence “the non-neuronal inter-organ linkage operating under hepatic PPARδ-to-muscle PPARα axis” to “the non-neuronal inter-organ linkage up-regulating muscle lipid utilization” in the Abstract.
