# Supplementary material for: Rspo3-mediated metabolic liver zonation regulates systemic glucose metabolism and body mass in mice
Source: PLoS Biol. 2025 Jan 24;23(1):e3002955. doi: 10.1371/journal.pbio.3002955 (PMC11759367; doi:10.1371/journal.pbio.3002955)
Supplement: Attachment — Submitted filename: Responses.docx [file pbio.3002955.s026.docx]

Responses to the Reviewers’ comments

Reviewer #2, Costas Christodoulides (note, Reviewer 2 has signed this review):

The authors have adequately addressed most of my concerns. However, I do have a few additional comments.

Major Comments:

1.	The authors state that modulating hepatic RSPO3 expression may "restore metabolic zonation." Is there any evidence, such as transcriptomic data either generated by the authors or publicly available, that hepatic zonation is disrupted in mouse models of dietary and genetically induced obesity?

We appreciate the reviewer’s important comments and also consider it to be necessary to confirm whether metabolic zonation in the liver is actually altered or disrupted in murine models of obesity.

As the reviewer suggested, we first performed new RNA-Seq analyses of the liver samples, which allowed us to compare the basal differences in hepatic gene expressions between the lean wild and the obese mice. Next, we performed gene ontology (GO) analysis of this RNA-Seq data by using the web-accessible program DAVID (Database for Annotation, Visualization, and Integrated Discovery), which integrates functional genomic annotations. The results obtained by this GO analysis have now been added to the supporting information as two bubble plots of up- and down-regulated GO terms (S5 Fig) as well as two bubble plots of the up- and down-regulated KEGG pathway (S6 Fig). These results confirm that obese conditions in murine models actually change several pathways associated with the GO terms of biological processes, cellular components and molecular functions. Most notably, the up-regulated GO terms and KEGG pathway results confirm that metabolic pathways such as glucose and lipid metabolism are mainly activated in obese mice rather than lean mice (S5 Fig and S6 Fig).

On the other hand, the original GO analysis of RNA-Seq data revealed that hepatic Rspo3 induction in obese mice suppresses metabolic pathways including those governing glucose and lipid metabolism (S10 Fig). Carefully considering these data collectively, hepatic Rspo3 induction in obese mice, in which hepatic endogenous Rspo3 expression is decreased, dramatically changes metabolic pathways, suggesting the reconstitution of these metabolic pathways. In other words, there is suppression of the pathways activated by obese conditions and thus a return to their stable states as observed under lean conditions**.**

Next, we performed a careful review of our new RNA-Seq data, which indicated that the pericentral (PC) metabolic genes such as Rnase4, Cyp1a2, Rdh9, and Avpr1 are decreased, while the periportal (PP) metabolic gene, Aldh1b1, is increased in the obese as compared to lean mice (S2 Table). In addition, according to qPCR analyses, hepatic gene expressions of LGR5 and Axin2, downstream targets of the Rspo3/Wnt signaling pathway as well as those of Cyp7a1 and Rdh9, PC metabolic genes, are decreased in obese mice (S7 Fig).

Taking these all-new data into consideration, there is a possibility that obesity actually changes PC and PP metabolic gene expressions as well as those of downstream target genes of the Wnt signaling pathway, thereby contributing to the pathophysiological alterations of hepatic metabolic zonation.

In addition to our data, a recent report highlighted the importance of the spatiotemporal atlas of the mouse liver including metabolic gene expressions by means of a novel transcriptome analysis approach (*Nat Genet* 56, 953-69, 2024). Furthermore, the authors of a review article speculated that hepatic metabolic gene expressions under the control of zonated PP or PC areas are disturbed by obesity and metabolic syndrome (*Ann Rev Path Mech Dis* 18, 439-66, 2023). Although the essence of the story differs slightly, the following point is also regarded as being very important. Recently, another new study was the first to reveal by spatially mapping the hepatic ER and mitochondria architecture, that fasting alters hepatic organelle morphology in a zonation-dependent manner, the dynamic flexibility of which is disturbed in obesity (*Nat Commun* 15, 3982, 2024).

Therefore, as described in the present report, hepatic Rspo3 induction in obesity is considered to be the result of restoration of this metabolic zonation alteration associated obesity development.

2.	Effects of Vagotomy: The authors claim that a neural inter-organ communication system is involved in hepatic RSPO3-induced actions in adipose tissues and skeletal muscle. However, according to the data in Supplementary Figure 12A, vagotomy abrogates the weight loss associated with hepatic RSPO3 overexpression in genetically obese mice. Could this be the primary reason for the gene expression changes in adipose tissues and skeletal muscle, as well as the changes in glucose tolerance and insulin sensitivity in hepatic RSPO3-overexpressing mice post-vagotomy? Also, I would like to see the effects of hepatic vagotomy on fasting glucose levels. Is it possible that hepatic vagotomy increases hepatic glucose output, thereby potentially contributing to a switch from fat to glucose metabolism in peripheral tissues in vagotomised RSPO3-expressing mice?

We appreciate the reviewer’s helpful comments and also consider it to be important to distinguish whether hepatic vagotomy (HV) can primarily inhibit Rspo3-induced remote effects on the distant organs via an inter-organ neuronal communication system.

First, the comparison of body weight gain between sham- and HV-LacZ control mice revealed that HV alone does not alter body weight gain in these LacZ control mice (S16A Fig). Thus, HV is considered to play a role in selectively inhibiting the reduction of body weight gain caused by hepatic Rspo3 induction.

In general, restriction of food intake is considered to be one of the major and fundamental causes underlying the reduction of body weight. In the original manuscript, hepatic Rspo3 induction was noted to decrease food intake after adenovirus administration (Fig 2L and Fig 3N), and we next examined whether there is a food intake difference between these two types of post-operative Rspo3 mice models. Interestingly, hepatic Rspo3 induction decreases food intake of either sham-operated or HV mice as compared to those of the corresponding LacZ control mice (S16B Fig), indicating that although there is a difference in degree, hepatic Rspo3 induction results in food intake decreases for these post-operative mice, regardless of which surgery was undertaken.

However, as described in the original manuscript, HV almost completely blocked the reduction of body weight gain derived from hepatic Rspo3 induction, an observation by which we can confirm the significant difference in body weight gains when comparing these sham-Rspo3 and HV-Rspo3 mice with the corresponding LacZ control mice (S16A Fig). Taking these different effects of hepatic Rspo3 on the regulations of body weight and food intake into consideration, the primary mechanism other than regulation of food intake, by which HV inhibits body weight reduction in response to hepatic Rspo3 induction, is considered to actually exist. This would be precisely the inter-organ neuronal communication system described in the present report, i.e., a system by which neuronal signals from the liver regulate sympathetic activity toward adipose tissues and skeletal muscle, leading to the regulation of their metabolic functions. In other words, metabolic gene expressions and insulin signaling are regulated, with whole-body pathophysiological states associated with obesity improving as a result. Therefore, hepatic Rspo3 induction in the setting of obesity is considered to primarily lead to stimulation of sympathetic activity toward WAT and skeletal muscle via an inter-organ communication system, followed by dramatic changes in metabolism in these remote organs, which collectively, ultimately, result in body weight reduction.

Second, to explore whether HV has any effect on the levels of fasting blood glucose (FBG) in our murine models, we carefully reviewed and compared the levels of sham-LacZ mice and HV-LacZ mice. This comparison revealed that HV alone does not result in a difference in FBG levels between two types of these post-operative control mice (sham-LacZ mice, FBG 184.7±54.6 mg/dL, HV-LacZ mice, FBG 208.0±33.2 mg/dL, *n.s.*, *n*=6-7). Of course, there are previous reports which indicated that the hepatic vagus, especially the efferent nerve, can inhibit hepatic gluconeogenesis by monitoring hypothalamic levels of glucose, fatty acids, etc. However, this inhibitory system does not appear to function in our present models.

Third, surprisingly, according to a careful review of our original RNA-Seq data and the new quantitative RT-PCR study, hepatic Rspo3 induction increases the expression of glycerol kinase (Gyk), which functions as the key enzyme for gluconeogenesis from lipids (glycerol), in the livers of obese mice (below, additional Fig1 in Responses). On the other hand, as noted in the original manuscript, hepatic Rspo3 induction decreased the expression of PEPCK, which functions as the key enzyme for gluconeogenesis from amino acids, in the livers of obese mice. In general, circulating glycerol is considered to be derived from lipolysis of adipose tissues through sympathetic nervous stimulation, and serum glycerol levels are actually increased in obese-Rspo3 mice (below, additional Fig2 in Responses). This phenomenon means that hepatic Rspo3 induction down-regulates PEPCK-mediated gluconeogenesis from amino acids originating in skeletal muscle, while it up-regulates Gyk-mediated gluconeogenesis from glycerol originating in adipose tissues. We can thus speculate that hepatic Rspo3 induction regulates gluconeogenesis in the liver by determining, via redistribution, whether its substrates come from skeletal muscle or adipose tissues. Of course, the possible mechanism by which hepatic Rspo3 induction redistributes substrates for gluconeogenesis from amino acids toward glycerol needs to be clarified in a future study. This important topic is clearly worthy of further investigation.

3.	In Figure 5, panels D, E, G, and J, please also include data from control LacZ mice.

As the reviewer suggested, we now present the data including expressions from sham operated or HV control LacZ and Rspo3 mice. Therefore, we had to re-analyze UCP1, PGC1α, and β3AR mRNA expressions in WAT and BAT or PGC1α and PPARα mRNA expressions in skeletal muscle of sham- and HV-Rspo3 mice. Although there are modest variations in the data, our results suggest that HV almost completely blocked the remote effects obtained by hepatic Rspo3 induction in obese mice (Fig 5D, 5G and 5J). These new data have been added to the revised manuscript.

Other Comments:

1.	Hepatic RSPO3 expression in lean animals approximately leads to a doubling of liver weight. This is not a slight change as stated by the authors (page 12, second from bottom line).

As the reviewer suggested, we removed the word “slightly” from this sentence.

2.	Duration of Metabolic Benefits: Additionally, 20 days is not a "relatively long period" and should not be reported as such by the authors. They should simply state that metabolic benefits lasted up to 20 days.

As the reviewer suggested, we removed the term “relatively long period” and corrected the relevant parts to “the beneficial effects of which persist for 20 days” (page 13, lines 5-6), “Rspo3 localization in the liver on day 23” (page 15, lines 9-10), and “the beneficial effects persisting for 20 days” (page 19, lines 12-13).

3.	Page 13, bottom of the second paragraph. The authors state that increased beta3AR, UCP1, and PGC1a expression in epididymal WAT contributes to decreased body weight after 7 days of adenoviral treatment. This is speculation. We are not shown any calorimetry data to support this. In fact, based on the authors' data, the weight loss in these animals is driven by reduced appetite.

As the reviewer suggested, we examined energy expenditure changes, such as O_2_ consumption (VO_2_) and the respiratory quotient (RQ) ratio, serving as gain-of-function models after Rspo3 adenovirus administration into lean mice. Hepatic Rspo3 induction in lean mice does not change VO_2_, but decreases RQ (Fig 2I), raising the possibility that it changes the balance of energy source use shifting toward utilization of lipids rather than carbohydrates. This phenomenon is compatible with up-regulation of gene expressions of β3AR, UCP1 and PGC1α in WAT, leading to the observed impacts on WAT volume reduction and body weight decrease. On the other hand, we examined whether there is a difference in food intake between lean LacZ and Rspo3 mice, revealing that hepatic Rspo3 induction in lean mice also decreases food intake (Fig 2L).

In fact, it was stated in the original manuscript that hepatic Rspo3 induction in obese mice decreases food intake, as compared to that of control mice. As described in the above “Major Comment 2”, the studies employing HV experiments in obesity models indicated that hepatic Rspo3 induction primarily stimulates sympathetic activity toward other organs outside the liver, leading to the WAT volume reduction and decreased body weight due to sympathetic activation rather than the regulation of food intake. However, we did not perform HV experiments in lean murine models, such that the precise mechanism must be clarified in a future study focusing on whether modulation of food intake or that of sympathetic activity would mainly be involved in the regulation of body weight in lean states.

Therefore, we corrected this statement to “hepatic Rspo3 induction significantly decreased the weight of epididymal WAT (Fig 2J) due to increased β3-adrenergic receptor (β3AR), UCP1, and PGC1α expressions in epididymal WAT (Fig 2K) and/or decreased food intake (Fig 2L), contributing to the prevention of body weight gain for 7 days after adenoviral treatment (Fig 2M)”. We believe that this clarification tones down the original statement.

4.	Page 16, second paragraph. The authors mention that hepatic RSPO3 induction regulates hepatic lipogenesis, but the only support for this is changes in gene expression of lipogenic genes. Hence, this statement should be tempered.

As the reviewer suggested, we corrected this statement to “hepatic Rspo3 induction up-regulated the expressions of SREBP1c and FASN (Fig 3F) as well as those of FASN (1.51-fold, *P* < 0.01) and other lipogenic genes examined by RNA-sequence analysis (S11 Fig)”. This change contributes to toning down the prior claim regarding the relationship between Rspo3 and hepatic lipogenesis.

5.	Page 20, top paragraph: "This so-called energy-saving machinery in obese …. with regulating BAT thermogenic physiology." This is speculation, hence this statement should be tempered and ideally moved to the discussion.

As the reviewer suggested, we moved the statement “This so-called energy-saving machinery in obese Rspo3-mice is partly explained by hepatic GK up-regulation, in which hepatic GK mediates an inter-organ linkage associated with regulating BAT thermogenic physiology” and the next sentence mainly into the discussion portion (page 31, lines 7-10, and page 31, line 19 to page 32, line 1, respectively).

6.	Page 20, bottom paragraph: "Hepatic RSPO3 induction…. is impaired in the setting of obesity." Again, this is speculation as the authors have not shown that adrenergic signaling, mitochondrial biogenesis, skeletal muscle mass, and muscle remodeling are improved consequent to hepatic RSPO3 expression. They just show gene expression changes.

As the reviewer suggested, we changed these statements to avoid over-interpretation (page 21, lines 13-18).

7.	In Figure 5I, the effects of vagotomy on norepinephrine content in WAT seem to be driven by an increased content in the LacZ mice rather than a decrease in the mice with hepatic RSPO3 overexpression.

We agree with the reviewer’s thoughtful insight that the effects of HV on NE contents of WAT seem to be derived from an increase of those in sham-LacZ mice rather than a decrease of those in HV-Rspo3 mice. However, the effect of increasing NE contents of WAT by hepatic Rspo3 induction, as observed in the sham-operated group, was almost completely blocked in the HV group.

In order to determine whether sympathetic stimulation toward WAT is actually involved in this increase in the NE contents of WAT, we performed a new experiment, in which the rate of NE turnover in WAT was evaluated by comparing the difference in the NE decrease, between LacZ and Rspo3 mice, due to treatment with α-methyl-p-tyrosine (α-MT), an inhibitor of catecholamine biosynthesis. The results obtained indicated that hepatic Rspo3 induction increased the NE contents of WAT, while the NE decrease in WAT after treatment with α-MT was significantly larger in Rspo3-mice (Fig 4F). This means that hepatic Rspo3 induction up-regulates NE production and NE turnover, considered to reflect sympathetic activity toward WAT.

Therefore, in the original manuscript, variations in the data may have been observed in WAT NE contents of HV experiments, but these new results of the NE turnover experiment can reasonably be taken to support the mechanism by which the hepatic vagus mediates stimulation of sympathetic nerves to WAT, resulting in up-regulation of NE production and turnover in WAT.

8.	The immunofluorescence images are of poor quality, and I suggest using higher resolution figures in any publication.

As the reviewer suggested, we now present immunofluorescence images with higher resolution than the original figures and intend to use them in the actual publication, following acceptance of the manuscript.

9.	The authors claim that Figure 1C shows that RSPO3 expression is reduced in BAT and skeletal muscle of obese mice. However, the bar charts seem to suggest the opposite.

As the reviewer noted, we carefully reviewed the original data, confirming that endogenous Rspo3 expressions in both adipose tissue types (WAT and BAT) as well as skeletal muscle are lower than that in the liver. In addition, endogenous Rspo3 expressions in both the liver and WAT diminish according to the degree of obesity. In BAT, endogenous Rspo3 expression in DIO mice is increased as compared to that of lean mice, while endogenous Rspo3 expression in ob/ob mice does not differ from that of lean mice. In skeletal muscle, endogenous Rspo3 expressions in both DIO and ob/ob mice are increased as compared to that of lean mice. However, in the major organs producing Rspo3, i.e. the liver and WAT, endogenous Rspo3 expressions are under the regulation of metabolic, such as lean or obese, states.

Therefore, to avoid misunderstanding, we clarified this issue by correcting the sentence “Rspo3 expressions were also decreased in the insulin responsive organs according to the degree of obesity” to “endogenous Rspo3 expressions in the liver and WAT, the major organs producing Rspo3, were decreased, while those in BAT and skeletal muscle were not, according to the degree of obesity”.

10.	Beta-catenin is post-transcriptionally, rather than transcriptionally, regulated by WNT/beta-catenin signaling. Unlike Axin2 and LGR5, it is not a beta-catenin target gene, and the references quoted by the authors do not support such a premise.

We agree with the reviewer’ comment and the notion that β-catenin is post-transcriptionally, but not transcriptionally, regulated by the WNT/β-catenin signaling pathway. On the other hand, in the original manuscript, we described only our examination of the gene expression of β-catenin, not the degree of its post-transcriptional modification in the livers of Rspo3 mice. Therefore, in the present manuscript, to avoid misunderstanding, we deleted the data obtained by hepatic β-catenin expressions measured using quantitative RT-PCR.

Reviewer #3:

This revised manuscript shows some improvement and the authors have attended to some concerns raised by the reviewers. They improved on systematically showing expression patterns of Rspo3 in the liver under various metabolic conditions and in response to feeling and fasting. They have used several models of obesity to show the importance of RSPO3 with overexpression and knockout. However, the study continues to have major concerns around their interpretation of data and overreaching of conclusions that are often stated even before data description in the results section. Major issue is around the acute short-term analysis for a disease model that is long term that limits the significance of the study. They have expanded only some models to 21 days which is still insufficient and also, would be important to show in a longitudinal fashion so that we can get some sense of continual/plateau versus temporary effect.

We appreciate the reviewer’s important and insightful comment that our present study seems to be a short-term analysis, even though it targets chronic diseases, i.e. obesity and diabetes, which may limit the practical applications of this study. However, the editors suggested that the generation of additional long-term analyses of hepatic Rspo3 expression would not be required, because there is still value in the present results. In fact, they also suggested that the absence of this long-term analysis should be mentioned in the discussion as a limitation.

Therefore, in last portion of the discussion section, we acknowledge this weakness, i.e., that long-term evaluations of the impacts of hepatic Rspo3 expression on whole-body metabolism were not performed, as a limitation of the present study.

The following are some specific comments:

Abstract -

The metabolic zonation in the liver is thereby broadly defined as 'spatiotemporal'

variability… it is not spaciotemporal - rather it's a 'spatial' phenomenon - but not temporal - thus the term zonation. This elaboration is not needed in the abstract in particular.

'Herein, we demonstrate that altered hepatic metabolic zonation due to Rspo3 contributes to maintaining systemic glucose metabolism and body composition via a new mechanism involving neuronal and non-neuronal inter-organ linkages' - this also is not a statement that should appear in the abstract before data description - pls remove and consider adding at the end if supported by overall data.

Similarly - the following sentence are not data description but rather conclusions. 'Hepatic Rspo3 induction in obesity improves obesity-associated features, e.g., fatty liver, insulin resistance and diabetes, by reversing adipose tissue enlargement and restoring attenuated organ insulin sensitivities, overstimulated adaptive thermogenesis and decreased skeletal muscle quality.' - It would be best to describe data.

The entire abstract is unacceptably composed of interpretation/conjecture rather than data description - would be best to describe/summarize data followed by overall conclusion.

We appreciate the reviewer’s important and helpful comments on our original Abstract. As the reviewer suggested, we have rewritten the entire of Abstract, making it easier to read and to understand the essence of our study.

Pg 7 - high-fat diet(s) - should be high fat diet

As the reviewer suggested, we corrected the term “high-fat diet(s)” to “high fat diet”.

Fig 1 - Nice work showing systematic data beginning with expression pattern in models of obesity and in lean conditions both fasted and fed. However, to conclude this section with "Therefore, targeting hepatic Rspo3 may allow new mechanisms involved in regulating metabolic homeostasis in the liver to be identified, which could in turn lead to the detection of remote effects on systemic metabolism with physiological (against obesity) or pathophysiological (promoting obesity) significance. Pg 11' is not at all appropriate and would be better to summarize their data.

We appreciate the reviewer’s positive comment on our precise identification of endogenous Rspo3 expression patterns in the livers of several murine models, as described in Fig 1. As the reviewer suggested, we removed the following statement: “Therefore, targeting hepatic Rspo3 may allow new mechanisms involved in regulating metabolic homeostasis in the liver to be identified, which could in turn lead to the detection of remote effects on systemic metabolism with physiological (against obesity) or pathophysiological (promoting obesity) significance.” Deletion of this sentences avoids over-interpretation at this stage (page 11, line 9).

'we expressed Rspo3' on pg 11 should be 'overexpressed'

As the reviewer suggested, we corrected the term “we expressed Rspo3” to “we overexpressed Rspo3”.

For fig 2A 2nd panel Y-axis label - should be 'relative intensity'?

As the reviewer pointed out, we originally indicated the ratio of western blotting band volumes from Rspo3 mice vs those from LacZ mice. To obtain western blotting band volumes from these mice, we measured them according to the manufacturer’s manual, ImageQuant TL. Then, as the reviewer suggested, we corrected the term “The ratio of volumes” to “Relative intensity” in the Y-axis labels of Fig 2A 2nd panel and the relevant panels presented thereafter.

For fig 2 - they show effects after 7 days and day 20 is only shown in S3 without corresponding weight data and also no description of this data in the results section. Body weight data needs to be shown in a longitudinal fashion with absolute weights rather than relative change. Metabolic expression data shown in fig 2 after 7 days also needs to be shown after 20 days - (genes shown in fig 2D).

As the reviewer suggested, we presented the body weight gain data corresponding to day 20 and showed the temporal changes in absolute body weight after adenovirus administration from day 0 to day 20 (Fig 2M). In addition, we examined hepatic metabolic gene expressions, as described in Fig 2C and 2D, of lean mice by quantitative RT-RCR on day 23 after adenovirus administration (S3F Fig).

Fig 2A does not show 'selective transgene expression in the liver' pg 11. For this statement to be supported, they need to show expression in other tissues.

As the reviewer noted, employing quantitative RT-PCR, we examined Rspo3 mRNA expressions in other organs, such as white and brown adipose tissues and skeletal muscle, of lean Rspo3-mice, which did not show significant alterations as compared to their expressions in control mice (S3A Fig).

'These results indicated hepatic Rspo3 induction to up-regulate the Rspo3/Wnt signaling pathway in the liver and thereby shifted metabolic gene expressions, a process by which hepatic features of metabolism are considered to move from the periportal to the pericentral zone. These phenomena, as described above, are compatible with those outlined in previous reports which indicated the roles of Rspo3 in regulating metabolic zonation [13] or reprograming of zonation in the liver [18]. (pg 12) is not supported by their data preceding this conclusion.

We appreciate the reviewer’s important comments and also consider it to be necessary to confirm whether hepatic Rspo3 induction shifts metabolic features from the periportal toward the pericentral zone.

First, we performed new RNA-Seq analyses of the liver samples, in which the basal differences in hepatic gene expressions were compared between the lean wild and the obese mice. Next, we performed gene ontology (GO) analysis of this RNA-Seq data by using the web-accessible program DAVID (Database for Annotation, Visualization, and Integrated Discovery), which integrates functional genomic annotations. The results obtained by this GO analysis have now been added to the supporting information, as two bubble plots of up- and down-regulated GO terms (S5 Fig) as well as two bubble plots of the up- and down-regulated KEGG pathway (S6 Fig). These results confirm that obese conditions in murine models actually change several pathways associated with the GO terms of biological processes, cellular components and molecular functions. Most notably, the results of up-regulated GO terms and the KEGG pathway confirm that metabolic pathways such as glucose and lipid metabolism are mainly increased in obese, while being only modestly altered in lean mice (S5 Fig and S6 Fig).

On the other hand, original GO analysis of RNA-Seq data revealed that hepatic Rspo3 induction in obese mice decreased these metabolic pathways, including those for glucose and lipid metabolism (S10 Fig). Carefully considering these data together, hepatic Rspo3 induction in obese mice, in which hepatic endogenous Rspo3 expression is decreased, dramatically changes metabolic pathways, suggesting the reconstitution of these metabolic pathways, namely suppressing those activated in response to obese conditions and returning them to a stable state as observed under lean conditions.

Next, we performed a careful review of our new RNA-Seq data, indicating that the pericentral (PC) metabolic genes such as Rnase4, Cyp1a2, Rdh9, and Avpr1 are decreased, while the periportal (PP) metabolic gene, Aldh1b1, is increased in obese as compared to lean mice (S2 Table). In addition, according to qPCR analyses, hepatic gene expressions of LGR5 and Axin2, downstream targets of the Rspo3/Wnt signaling pathway as well as those of Cyp7a1 and Rdh9, PC metabolic genes, are decreased in obese mice (S7 Fig). Furthermore, alterations of these PP and PC metabolic gene expressions associated with obesity were normalized by hepatic Rspo3 induction, suggesting that regulation of Rspo3 can shift metabolic features from the periportal toward the pericentral zone.

However, since the above information is not necessarily backed up by accurate data in lean Rspo3 mice, we corrected the relevant statements and removed “These phenomena, as described above, are compatible with those outlined in previous reports which indicated the roles of Rspo3 in regulating metabolic zonation [13] or reprograming of zonation in the liver [18]”, thereby toning down this revised version (page 12, line 6).

To examine proliferative capacity of the liver in response to Rspo3 overexpression, Ki67 should be shown histologically. It is not clear what the proliferative impact of Rspo3 overexpression is, particularly given the known impact in malignancy. This might explain the increase in liver weight.

As the reviewer suggested, we performed Ki-67 immunofluorescence analysis of the liver (S4H Fig) as well as quantitative RT-PCR experiments on hepatic Ki-67 expression (S4I Fig). These results indicated hepatic Rspo3 induction to increase both Ki-67 mRNA and protein levels, but the significance of the proliferative impact of Rspo3 induction is not yet fully understood and warrants further detailed examination in a future study.

Fig 3 - using ob/ob - again - only examined after 7 days with minimal difference in body weight - absolute body weight in a longitudinal fashion need to be shown for the two groups rather than a relative change as shown in Fig 3M.

As the reviewer suggested, in addition to the body weight gain data, we now present the temporal changes in absolute body weight after adenovirus administration from day 0 to day 23 (Fig 3M).

'Rspo3 induction increased Ki67 IF activity (S4H and S4I Fig)' (pg 15) is not true-. IF does not measure activity.

As the reviewer suggested, we corrected the term “Rspo3 induction increased Ki-67 IF activity” to “Rspo3 induction increased Ki-67 IF intensity”.

p-Akt in the livers of ob/ob mice (Fig 3D) needs to be quantified and need more n. n=2 with intensity shown above that ranges widely and is not sufficient.

As the reviewer suggested, we re-examined the immunoblotting results against phospho-Akt- and Akt-antibodies in the livers of ob/ob mice (Fig 3D) by using 3 samples from each group. The results showed more clearly that hepatic Rspo3 induction up-regulates insulin-stimulated phosphorylation of Akt in obese mice. Accordingly, the originally presented data were replaced with these new results.

These improvements of glucose 'metabolism' (pg 18) - should be 'tolerance'

As the reviewer suggested, we corrected the term “These improvements of glucose metabolism” to “These improvements of glucose tolerance”.

murine models (S11A and S11B Fig), resulting increased resting VO2 as compared to that of lean mice (S11C Fig) - should also include once overexpressing Rspo3. Otherwise these data are not helpful for their manuscript.

As the reviewer noted, we carefully reviewed the data on energy expenditure presented in the original manuscript (S15C Fig and Fig 4H). The comparison of these data indicate that obese conditions up-regulate basal O_2_ consumption (VO_2_) and down-regulate the respiratory quotient (RQ), the underlying phenomena of which are almost completely reversed by hepatic Rspo3 mice induction in these obese mice. Therefore, these data are considered to support the conclusions of the present study.

'This so-called energy-saving machinery in obese Rspo3-mice is partly explained by hepatic GK up-regulation, in which hepatic GK mediates an inter-organ linkage associated with regulating BAT thermogenic physiology [30]. These results raise the possibility that the reconstitution of hepatic metabolic zonation by Rspo3 induction in obesity, with up-regulation of WAT-derived thermogenesis and restoration of overstimulated BAT-derived thermogenesis, leads to balancing and redistribution of thermogenesis derived from different adipose tissues such as WAT and BAT.' (pg 20) - would advise removing from results section. These are all expected findings with protection from weight gain. To implicate adrenergic nerve involvement etc is a complete overstretch. Would advise involving a scientist in metabolism to properly interpret these results.

As the reviewer suggested, we moved these statements from the results section to the discussion.

Although the reviewer noted that to implicate adrenergic nerve involvement is a complete overstretch, taking all of the results of the present study together, neuronal signals via the hepatic vagus obtained after Rspo3 induction in the liver seem to be involved in the novel inter-organ communication system. In addition, some of the reasons for this belief are explained in the next two sections.

Implication of vagal nerve in RSPO3 action is not at all clear nor with good rationale. Furthermore, the data presented are with large error bars and not meaningful. HV is not at al helpful in the results and adds to the confusion and distracts the main story. Would suggest removing. It reflects the naïve understanding of their data.

As the reviewer noted, we presented all of the data by using the average ± standard deviation (SD), but not standard error (SE), which on occasion appeared to show that the data have rather large error bars. However, taking all of the results of the present study together, neuronal signals via the hepatic vagus obtained after Rspo3 induction in the liver are involved in the new inter-organ communication system. In addition, some of the reasons for this belief are explained in the following section.

The scheme shown in S15 is not at all supported by their data and reflects very poorly on their understanding of their data. Would advise removing any neuronal involvement and keeping purely the data in the different tissues that are known effects of improvement in hepatic insulin sensitivity.

We appreciate the reviewer’s important suggestions. First of all, we previously clarified that new inter-organ communication mechanisms, in which neuronal signals from the liver regulate metabolic homeostasis of remote organs, are inherent in mammals. These communication systems have many important functions in regulating basal energy expenditure at the whole-body level (*Science* 312, 1656-9, 2006, *Cell Metab* 16, 825-32, 2012, etc.), insulin secretion from pancreatic β cells (*Science* 322, 1250-4, 2008, *Nat Commun* 8, 1930, 2017), lipid metabolism (*Nat Commun* 6, 7940, 2015), etc. Furthermore, more recently, another laboratory collaborating with ours demonstrated that optogenetic modulation of efferent vagal nerves to the pancreas improved diabetes, leading to speculation that there would be medical applications of the inter-organ communication system (*Nat Biomed Eng* 8, 808-22, 2024).

Second, to reinforce our results regarding the core of neuronal inter-organ communication in this study, we examined whether hepatic Rspo3 induction has any effect on the intensity of *c-fos* immunofluorescence in the brain, especially in the rostral raphe pallidus nucleus (rRPa), which contains the putative sympathetic premotor neurons responsible for thermogenesis in BAT. The results obtained indicated that hepatic Rspo3 induction significantly decreases the number of *c-fos* positive neurons in rRPa as compared to that of control mice (S14 Fig).

Third, in order to determine whether sympathetic stimulation of WAT is actually involved in this increase in the NE contents of WAT, we performed a new experiment, in which the rate of NE turnover in WAT was evaluated by comparing the difference in the NE decline, due to treatment with α-methyl-p-tyrosine (α-MT), an inhibitor of catecholamine biosynthesis, between LacZ and Rspo3 mice. These results indicated that hepatic Rspo3 induction increased NE contents of WAT, while, notably, the NE decline in WAT after treatment with α-MT was significantly larger in Rspo3-mice (Fig 4F). This means that hepatic Rspo3 induction up-regulates NE production and NE turnover, which are considered to reflect sympathetic activity toward WAT.

Fourth, we examined whether there are NE-induced influences on VO_2_ in obese Rspo3 mice, and obtained results indicating that hepatic Rspo3 induction blunts the elevation of VO_2_ after NE treatment (Fig 4I). This is considered to reflect down-regulation of sympathetic activity toward BAT in obese Rspo3 mice. On the other hand, examination of NE-induced influences on VO_2_ in Rspo3-suppressed mice, indicated that suppression of hepatic Rspo3 increases the elevation of VO_2_ after NE treatment (Fig 6I). This is considered to reflect up-regulation of sympathetic activity toward BAT in Rspo3-suppressed mice.

Therefore, taking all of the additional results included in the present revision together, the novel notion arises that neuronal signals, transmitted via the hepatic vagus, obtained after Rspo3 induction in the liver are involved in the newly-identified inter-organ communication system.

For the KO data, decrease of less than 20% expression with such phenotype is highly questionable and hard to explain. Also, their IF data (S14I) does not show deletion to any appreciable degree. Also, short term data bring limited clinical relevance with no body weight data.

As the reviewer noted, our analysis of the degree changes in the Rspo3 protein levels in the knockdown experiments indicated that suppression of Rspo3 expression in the present study reaches a level comparable to the decrease in its protein, by almost 50 %, as compared to that of control mice (Fig 6A). It is generally known that there is a difference as well as variety in expressions between mRNA and protein levels. Thus, there appears to be a discrepancy in that the degree of Rspo3 suppression in iLiRspo3KO mice differs in mRNA levels versus protein levels. However, biologically, this is not necessarily an invalid observation, and we agree that this phenomenon exists.
